# Bioinformatic and phylogenetic analysis of the *CLAVATA3/EMBRYO-SURROUNDING REGION (CLE)* and the *CLE-LIKE* signal peptide genes in the Pinophyta

**DOI:** 10.1186/1471-2229-14-47

**Published:** 2014-02-14

**Authors:** Timothy J Strabala, Lorelle Phillips, Mark West, Lisa Stanbra

**Affiliations:** 1 Scion, 49 Sala St, PO Box 3020, Rotorua 3010, New Zealand

**Keywords:** CLE peptide ligands, CLEL peptide ligands, Pinophyta, Conifers, Phylogenetic analysis, Pine tracheary element system

## Abstract

**Background:**

There is a rapidly growing awareness that plant peptide signalling molecules are numerous and varied and they are known to play fundamental roles in angiosperm plant growth and development. Two closely related peptide signalling molecule families are the *CLAVATA3-EMBRYO-SURROUNDING REGION* (*CLE*) and *CLE*-*LIKE* (*CLEL*) genes, which encode precursors of secreted peptide ligands that have roles in meristem maintenance and root gravitropism. Progress in peptide signalling molecule research in gymnosperms has lagged behind that of angiosperms. We therefore sought to identify *CLE* and *CLEL* genes in gymnosperms and conduct a comparative analysis of these gene families with angiosperms.

**Results:**

We undertook a meta-analysis of the GenBank/EMBL/DDBJ gymnosperm EST database and the *Picea abies* and *P. glauca* genomes and identified 93 putative *CLE* genes and 11 *CLEL* genes among eight Pinophyta species, in the genera *Cryptomeria*, *Pinus* and *Picea*. The predicted conifer CLE and CLEL protein sequences had close phylogenetic relationships with their homologues in *Arabidopsis.* Notably, perfect conservation of the active CLE dodecapeptide in presumed orthologues of the *Arabidopsis* CLE41/44-TRACHEARY ELEMENT DIFFERENTIATION (TDIF) protein, an inhibitor of tracheary element (xylem) differentiation, was seen in all eight conifer species. We cloned the *Pinus radiata CLE41/44-TDIF* orthologues. These genes were preferentially expressed in phloem *in planta* as expected, but unexpectedly, also in differentiating tracheary element (TE) cultures. Surprisingly, transcript abundances of these TE differentiation-inhibitors sharply increased during early TE differentiation, suggesting that some cells differentiate into phloem cells in addition to TEs in these cultures. Applied CLE13 and CLE41/44 peptides inhibited root elongation in *Pinus radiata* seedlings. We show evidence that two *CLEL* genes are alternatively spliced via 3′-terminal acceptor exons encoding separate CLEL peptides.

**Conclusions:**

The *CLE* and *CLEL* genes are found in conifers and they exhibit at least as much sequence diversity in these species as they do in other plant species. Only one CLE peptide sequence has been 100% conserved between gymnosperms and angiosperms over 300 million years of evolutionary history, the CLE41/44-TDIF peptide and its likely conifer orthologues. The preferential expression of these vascular development-regulating genes in phloem in conifers, as they are in dicot species, suggests close parallels in the regulation of secondary growth and wood formation in gymnosperm and dicot plants. Based on our bioinformatic analysis, we predict a novel mechanism of regulation of the expression of several conifer CLEL peptides, via alternative splicing resulting in the selection of alternative C-terminal exons encoding separate CLEL peptides.

## Background

Since the identification of *CLAVATA3* (*CLV3*) in the dicot *Arabidopsis thaliana*[1], homologues and/or orthologues of this gene, known as the *CLV3-EMBRYO-SURROUNDING REGION* (*CLE*) gene family [2-4], have been identified in nearly every major plant phylogenetic clade from which large-scale genomic or EST sequence data are available, including monocots (rice, wheat) and a bryophyte moss (*Physcomitrella patens*). The functional roles for most *CLE* genes are still unknown. However, the roles for all *CLE* genes that have been established, including *CLV3*, are in the regulation of seed development [5] or the homeostasis of meristematic tissues reviewed in [6], including the shoot apical meristem (SAM) (*CLV3*) [1,7], root apical meristem (RAM) (*CLE*40) [8], vascular cambium (*CLE41/44-TRACHEARY ELEMENT DIFFERENTIATION FACTOR* (*CLE41/44-TDIF)*) [9], and root nodule meristems in several legume species (LjCLE-RS1/2; MtCLE12/13; GmRIC1/2) [10-12]. As such, CLE peptides play critical roles in the establishment, regulation and maintenance of plant architecture from the earliest stages of development.

**Figure 1:**
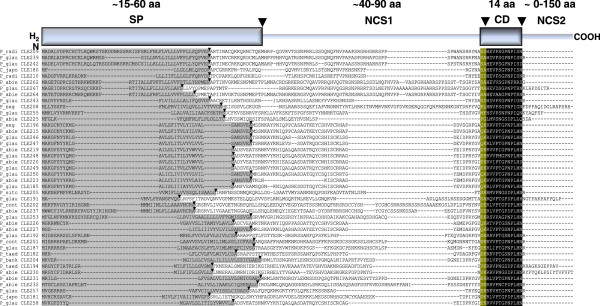
**Multiple alignment of representative predicted Pinophyta CLE protein amino acid sequences.** A schematic diagram of a generic CLE protein representing the main features of CLE proteins is shown above the alignment. The amino (H_2_N) terminus of the schematic protein is followed by the signal peptide (SP), the first non-conserved sequence (NCS1), the CLE domain (CD) and the second non-conserved sequence (NCS2) found at the COOH terminus of some CLE proteins. Presumed cleavage sites of the SP and the mature CLE peptide sequence are indicated by *large arrowheads*. The multiple alignment depicts the individual SPs of each putative full-length protein sequence with *grey highlighting.* The SignalP 4.1-predicted cleavage sites are indicated by the *small arrowheads.* The CLE motif, comprising the CLE peptide sequences and the two semi-conserved amino acids at the amino termini of the predicted CLE peptides, is indicated by *white lettering*. The predicted CLE peptides are indicated by *black highlighting* and the remaining sequence of the CLE domain is indicated by *dark yellow highlighting.*

Although putative *CLE* genes have been identified in monocot species that appear to encode multiple CLE peptides that are presumably post-translationally processed [3], most plant *CLE* genes are readily identified by several common structural motifs (Figure 1). Generally, the precursor protein coding sequence is approximately 240-300 nt (80-100 aa) in length. Within these sequences are found signal peptide motifs ranging in length from 45-90 nt (15-30 aa), followed by highly degenerate non-conserved sequences (NCS1) ranging from ~120-240 nt (40-80 aa) followed by the CLE motif [2-4], a 42 nt (14 aa) segment that contains the mature CLE peptide sequence, which is reported to be a 12-13 aa hydroxyprolinated, triarabinosylated peptide in *Arabidopsis*[13-15]. In most cases, the two amino-terminal amino acids of the 14 aa CLE motif are not found in the mature peptides, despite their conservation across species. There is evidence that these amino acids (and perhaps others nearer to the N-terminus of the precursor protein) constitute a protease recognition site involved in the post-translational processing of the precursor protein into mature CLE peptides [16,17]. Generally, the *CLE* gene protein-coding sequences terminate with the C-terminal amino acid of the mature CLE peptide. However, not all *CLE* genes conform to this paradigm, and C-terminal non-conserved sequences (NCS2) ranging from 3 to 450 nt (1-150 aa) have been observed in *CLE* genes from various species (Figure 1). These sequences are apparently trimmed from the precursor protein by a carboxypeptidase activity [16-18].

**Figure 2:**
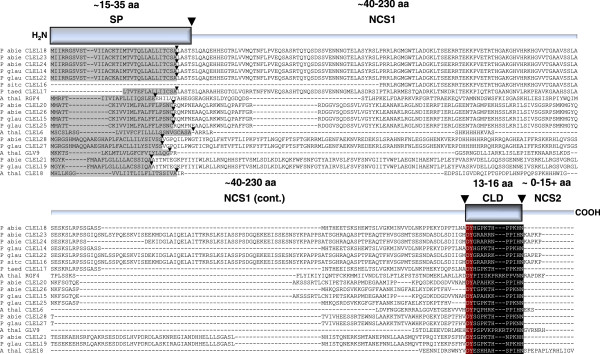
**Multiple alignment of *Arabidopsis *and predicted Pinophyta CLEL protein amino acid sequences.** A schematic diagram of a generic CLEL protein representing the main features of CLEL proteins is shown above the alignment. The amino (H_2_N) terminus of the schematic protein is followed by the signal peptide (SP), the first non-conserved sequence (NCS1), the CLEL domain (CLD) and the second non-conserved sequence (NCS2) found at the COOH terminus of some CLEL proteins. Presumed cleavage sites of the SP and the mature CLEL peptide sequence are indicated by *large arrowheads*. The multiple alignment depicts the individual SPs of each putative full-length protein sequence with *grey highlighting.* The SignalP 4.1-predicted cleavage sites are indicated by the *small arrowheads.* The CLEL motif, including the two conserved asp-tyr amino acids at the amino termini of the predicted CLEL peptides, is indicated by *white lettering*. The predicted CLEL peptides are indicated by *black highlighting* except for the asp-tyr sequence, which is indicated by *brick red highlighting.*

In contrast to the *CLE* family, the *ROOT GROWTH FACTOR*/*CLE-LIKE*/*GOLVEN* (*RGF*/*CLEL*/*GLV*) gene family has only recently been identified and described [19-21]. Like the *CLE* genes, they encode short, secreted peptides that affect aspects of plant development. Structurally, the *RGF/CLEL/GLV* genes are similar to the *CLE* genes in that they encode precursor proteins with a signal peptide, followed by an NCS1 region with a C-terminally oriented 12-15 aa peptide that is post-translationally processed to the active form (Figure 2). Also like the *CLE* genes, some *CLEL* genes encode proteins with C-terminal NCS2 regions of varying lengths (Figure 2). The CLEL peptides, as their name suggests, have very similar sequences to the CLE peptides. A key difference between the CLE and CLEL peptides is that the CLEL peptides are variable in length at 13-16 amino acids, as compared to the 12 amino acids of the CLE peptides. Perhaps the most salient distinguishing feature between CLE and CLEL peptides is the aspartic acid-tyrosine pair at the N-termini of all but one the RGF/CLEL/GLV active peptides. The sole exception to this rule is found in the GLV9 peptide, which contains a functionally conserved glutamic acid residue at its N-terminus in place of aspartic acid [21]. At least some of the CLEL peptides are post-translationally tyrosine sulphated, which is essential for aspects of their activity *in vivo*, including RAM homeostasis [19]. Interestingly, the conserved amino-terminal asp-tyr pair of the CLEL peptides is a characteristic shared with the sulphotyrosine peptide ligands PLANT PEPTIDE CONTAINING SULFATED TYROSINE 1 (PSY1) and PHYTOSULFOKINE (PSK) [22,23]. However, PSK and PSY1 are not otherwise similar to the CLEL peptides.

Meng et al. [20] pointed out that the RGF/CLEL/GLV peptides possess sequences that are similar to the CLE peptides. In fact, this group noted that the *CLE18* gene also possessed a *CLEL* motif near its C-terminus in addition to its *CLE18* motif, which is located in the middle of the protein. They went on to demonstrate that this CLEL motif conferred long roots to *Arabidopsis* plants when exogenously applied to roots in the form of a synthetic peptide. For this reason and the fact that not all of the so-called *RGF* genes had expression patterns restricted to roots, they named the gene family *CLE-LIKE* (*CLEL*). We use this nomenclature throughout the remainder of this paper.

Based on the combined analyses of Matsuzaki et al. [19], Meng et al. [20] and Whitford et al. [21], there are at least twelve *CLEL* genes in the *Arabidopsis* genome, including *CLE18,* which contains both *CLE* and *CLEL* domains [20]. Whitford et al. [21] also identified 13 *CLEL (GLV)* genes in rice (*Oryza sativa*) and eleven *CLEL* genes in quaking aspen (*Populus tremuloides*). As more recently identified genes/peptides, less is understood about the *CLEL* family in terms of their posttranslational processing and mode(s) of action. Matsuzaki et al. [19] demonstrated that a tyrosine-sulphated form of CLEL8 (RGF1) restored RAM maintenance of a tyrosine sulphotransferase mutant in conjunction with PSK and PSY1. Root waving has been reported to result from the application of the CLE18 CLEL peptide [20] and agravitropism has been reported in *clel* (*glv*) mutants [21].

Although the vast majority of extant land plant species are angiosperms, the gymnosperms, primarily the conifers, constitute approximately one-third of earth’s forest biomes [24], covering approximately 15% of global land area, primarily in the boreal forest [24]. A substantial fraction of the world’s wood and wood products are derived from conifer species. Therefore, understanding the molecular basis for conifer growth and development, particularly wood formation, is critical for improvement of commercial forest productivity, necessary to meet increasing global demands for wood and wood products without increasing the rate of global deforestation [24].

Despite the economic importance of conifers, relatively little is known about growth regulation in these species. For example, no peptide ligand has been described in any gymnosperm species to date. As the CLE and CLEL peptide ligands are broadly conserved families of regulatory molecules of fundamental importance to the maintenance of meristematic tissues as well as other developmental processes, we sought to identify expressed gymnosperm homologues of these genes as a first step toward understanding the roles of peptide ligands and meristem regulation in this major phylogenetic clade.

## Results

### Identification of conifer *CLE* and *CLEL* genes from public EST and genome sequence data

TBLASTN searches for *CLE* and *CLEL* genes in public gymnosperm EST databases initially yielded 81 candidate *CLE* gene ESTs only in eight different Pinophyta species. Contig analysis yielded 31 unique contigs. Manual validation of the putative *CLE* gene sequences resulted in the elimination of one contig from *Chamaecyparis obtusa*, due to weak sequence conservation, a truncated open reading frame for the presumed *CLE* gene and a clear open reading frame on the opposite strand. Thus, a total of 79 Pinophyta EST sequences in 30 contigs from seven different species were identified as predicted *CLE* genes (Additional file 1: Figure S1).

We also conducted a TBLASTN search in the NCBI/EMBL/DDBJ gymnosperm EST databases for *CLEL* family members using the *A. thaliana* CLEL motif sequences. This search yielded nine ESTs, again only from conifer EST databases, from five different species. Six unique contigs were constructed from these ESTs. After manual validation and a second query with the identified conifer *CLEL* genes, 10 ESTs in five contigs were identified as predicted *CLEL* genes (Additional file 2: Figure S2).

The CLE peptide motif sequences from the 30 predicted *CLE* genes identified in the EST searches were used to query the recently published genome sequences of two spruce species, *Picea abies* and *P. glauca*[25,26] for the genomic copies of the *P. glauca* EST sequences, as well as to identify other members of the *CLE* gene family not previously detected in EST sequencing projects. This search resulted in the identification of 93 apparent *CLE* genes (including presumed orthologous and paralogous genes) containing 36 different *CLE* sequences among the eight conifer species (Additional file 3: Table S1). Only three of the predicted *CLE* genes had introns (Additional file 3: Table S1). Subsequent queries of the spruce genomes with *Arabidopsis* CLE motifs did not identify any additional *CLE* genes.

**Figure 3:**
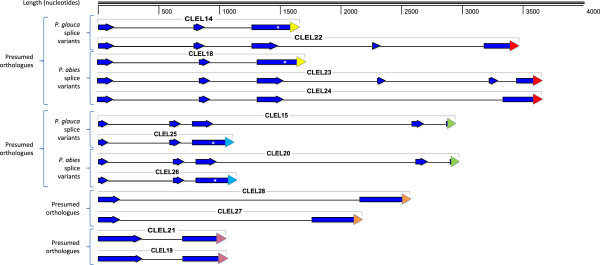
**Comparison of predicted *Picea abies *and *P. glauca CLEL *transcript exon/intron structures and splice variants.** The diagram depicts the portions of the predicted transcripts including and between the predicted initiator and terminator codons of the genes, omitting the 5′-untranslated regions and 3′-untranslated regions of the predicted transcripts. The transcripts are drawn to scale, with the scale bar at the top of the figure. The predicted transcripts are grouped by predicted splice variants and presumed orthologues designated by *labelled brackets* at the left of the figure and with the *P. abies* members of each set as the upper transcript(s) of the set. Predicted exons are depicted as *blue arrows* and predicted introns are depicted as *black lines*. Predicted alternative splice sites that result in selection of alternative CLEL peptide sequences are depicted as *white asterisks.* The CLE peptide domains are depicted as *coloured triangles.* Triangles with the same colour represent presumed orthologous peptides.

As with the *CLE* genes, the five CLEL peptide motifs were used to query the *P. abies* and *P. glauca* genome sequences. Unlike the *CLE* genes, although many putative CLEL peptide domains were identified, we could not identify any *CLEL* sequences that were directly downstream of a putative signal peptide domain within a continuous open reading frame (data not shown). Thus, it seemed likely that there were no *CLEL* genes that lacked introns within the genomes of these two organisms. Therefore, the full *CLEL* EST contig sequences were used in TBLASTN searches of the *P. abies* and *P. glauca* genome sequences. The results of these searches revealed extensive regions of alignment in non-contiguous segments within several genomic scaffolds of both of these species. These alignments provided approximate guides to intron/exon splice junctions to enable the construction of gene models based on the genome sequence (Figure 3). From this analysis, the predicted genomic sequences and gene structures of the two full-length *P. glauca* EST contigs *CLEL14* and *CLEL15* were readily identified (Figure 2, Figure 3, Additional file 3: Table S2), and these sequences were used to identify their presumed *P. abies* orthologues, *CLEL18* and *CLEL20*, respectively, due to the high degree of sequence conservation between these two species (Figure 2, Figure 3, Additional file 3: Table S2).

Using the *P. engelmannii* × *glauca* CLEL13 partial protein sequence as a query yielded the *P. glauca* gene *CLEL19* and its presumed *P. abies* orthologue, *CLEL21* (Figure 2, Figure 3, Additional file 3: Table S2). Both of these genes had only one intron (Figure 3). The identification of a putative *CLEL* sequence in a *P. glauca* genome sequence scaffold led us to search for a presumed 5′ exon containing a putative signal peptide, resulting in the identification of the predicted genes *CLEL27* and *CLEL28* (Figure 3, Additional file 3: Table S2). These are the only predicted *CLEL* genes we identified that are not validated by at least some EST evidence for expression or structure. Like *CLEL19* and *CLEL21*, these genes are predicted to have only one intron each and the predicted *CLEL28* intron is the longest of any of the introns we identified in these genes.

### Predicted *CLEL* genes encode alternatively spliced transcripts with different CLEL peptide domains

Interestingly, the TBLASTN search using the *P. sitchensis CLEL16* partial protein sequence (Additional file 3: Table S2), although not full-length, revealed that this gene also aligned with the same genomic scaffold as *CLEL14*, but the alignment included putative protein coding segments not found in *CLEL14* mapping to a long segment in NCS1 that is not shared by CLEL14 and CLEL17 (Figure 2). This prompted us to investigate possible alternative splicing in this gene, using the *CLEL16* alignment as a guide. This resulted in the identification of an excellent alternative splice donor sequence (exon…AG^GTA…intron) in the middle of the terminal coding exon of *CLEL14* (Figure 3, depicted by the white asterisk in the CLE14 schematic) and from this we identified the alternative transcript encoding the putative *P. glauca* protein CLEL22 (Figure 3, Additional file 3: Table S2), which is 98% identical to the *P. sitchensis* partial predicted protein sequence (data not shown) and encodes a CLEL peptide sequence from a different exon than that encoding the CLEL14 peptide (Figure 2, Figure 3, Additional file 3: Table S2). Examination of the presumed *CLEL14* orthologue *CLEL18* in *P. abies* for a similar splice variant yielded two genes, *CLEL23* and *CLEL24*, which encode nearly identical protein sequences encoded by two different sets of exons due to apparent exon duplication within the locus (data not shown), with the protein sequences differing only by a 16 aa indel toward their C-termini (Figure 2, Figure 3, Additional file 3: Table S2). This alternative splicing structure encoding nearly identical proteins was not found in *CLEL22* in *P. glauca*.

The discovery of alternative splicing in the *CLEL14/CLEL22* and *CLEL18/CLEL23/CLEL24* genes led us to search for splice variants in *CLEL15* and *CLEL20* in *P. glauca* and *P. abies* respectively, as multiple CLEL domains were also identified in these scaffolds. These searches revealed the alternatively spliced *CLEL25* and *CLEL26* in the *P. glauca* and *P. abies* genomes, respectively (Figure 2, Figure 3). All the predicted alternatively spliced *CLEL* gene pairs (with *CLEL23* and *24* considered as one half of a “pair” with *CLEL18*) encode transcripts that have distinct CLEL peptide sequences.

### Meta-analysis of conifer *CLE* and *CLEL* gene expression

Meta-analysis of the public EST sequence data showed that most of the *CLE* genes were identified in bark (phloem; *CLE180, 183, 190, 191, 196, 197, 198, 199, 201, 202, 203, 208*), xylem (*CLE186, 187, 192, 193, 200, 201, 204*) root (*CLE182, 184, 186, 188, 189, 194*) or mixed tissue (*CLE186, 190, 191, 193*) libraries (Additional file 3: Table S1). The genes that were not observed in bark, xylem or root libraries were mostly cloned from shoot/foliage (*CLE185, 186, 192, 195, 200, 205, 206, 207*) libraries (Additional file 3: Table S1), with the exception of *CLE180*, which was also identified in a male strobilus library in addition to bark (Additional file 3: Table S1). *CLE182* was the only conifer *CLE* gene identified from a developing embryo library and this gene was also found in both untreated and paraquat-treated root tissues of germinated plants (Additional file 3: Table S1). There was almost no overlap in the *CLE* genes identified between xylem and phloem tissues. The sole exception to this observation was *CLE201*, which was found in *Pinus contorta* xylem and bark libraries from wounded trees (Additional file 3: Table S1).

In contrast to the *CLE* genes, no *CLEL* gene was identified from xylem in our EST sequence meta-analysis. *CLEL* genes were primarily identified in root (*CLEL14, 17*) and shoot (*CLEL15, 16*) libraries, with only *CLEL13* identified in a bark library (Additional file 3: Table S2). Among the *CLEL* genes, only *CLEL14* and *CLEL15* were identified in more than one library, although these were not from different tissue types (Additional file 3: Table S2).

Contig analysis of the *CLE* and *CLEL* ESTs showed very good agreement among individual reads, even among sequencing projects of different laboratory groups, which presumably used different genotypes. As expected, most of the sequence differences between contiguous transcripts were found in the predicted 5′- and 3′-UTR regions of these contigs. Only one indel that could not be attributed to a potential sequencing artefact was observed, a 20 bp insertion in the predicted 5′-UTR of one *CLE182* transcript, which appears to be a direct repeat of the immediately following 20 bp segment (Additional file 1: Figure S1C). Nucleotide sequence differences resulting in differences in amino acid sequence were found in *CLE195*, *198*, *199* and *200* (Additional file 1: Figure S1P, S, T, U, respectively). Predicted silent mutations were also observed in *CLE195* and *CLE200* (Additional file 1: Figure S1P, U). Among the *CLEL* ESTs, only *CLEL15* showed differences, with seven amino acid differences between presumed alleles, as well as two apparent silent differences in sequence (Additional file 2: Figure S2C). There was an apparent frameshift between two *CLEL17* sequences (Additional file 2: Figure S2E), but this appears likely due to an error in one of the sequences. The longer open reading frame was chosen to represent the CLEL17 protein (Additional file 2: Figure S2E, Figure 2), as this sequence was predicted to encode a signal peptide (Figure 2, Additional file 3: Table S2). This open reading frame was confirmed by the genomic sequences of putative orthologues of this gene from *P. glauca* and *P. abies* (Figure 2, Additional file 3: Table S2).

### Bioinformatic and phylogenetic analysis of the conifer *CLE* and *CLEL* gene contigs

Predicted amino acid sequences of the *CLE* and *CLEL* EST consensus contigs were further analysed to determine the presence of putative signal peptides in their sequences. SignalP 4.1 [27] analysis of all CLE and CLEL amino acid sequences shows that all the predicted full-length proteins possess predicted signal peptides, as expected of functional CLE and CLEL proteins (Figures 1 and 2, Additional file 3: Tables S1, S2).

Multiple alignment of the predicted conifer CLE and CLEL amino acid sequences revealed that several *CLE* genes apparently have multiple highly conserved copies in the genomic sequences, with several scaffolds harbouring identical, or nearly identical sequences (Additional file 3: Table S1). Intriguingly, the genes encoding the identical CLE232 and CLE233 proteins are found on the same scaffold (Additional file 3: Table S1), suggesting that many of these duplicated *CLE* and *CLEL* scaffolds could indeed be duplicate genes within these large genomes.

Strong sequence conservation among presumed orthologous genes across species (and genera) was also observed. For example, among the predicted proteins CLE186 (*Picea glauca*), CLE206 (*Picea sitchensis*) and CLE201 (*Pinus contorta*) (Figure 1), CLE186 and CLE206 show 100% sequence conservation between these two spruce species, and these are 84.4% identical to the *Pinus* protein sequence.

**Figure 4:**
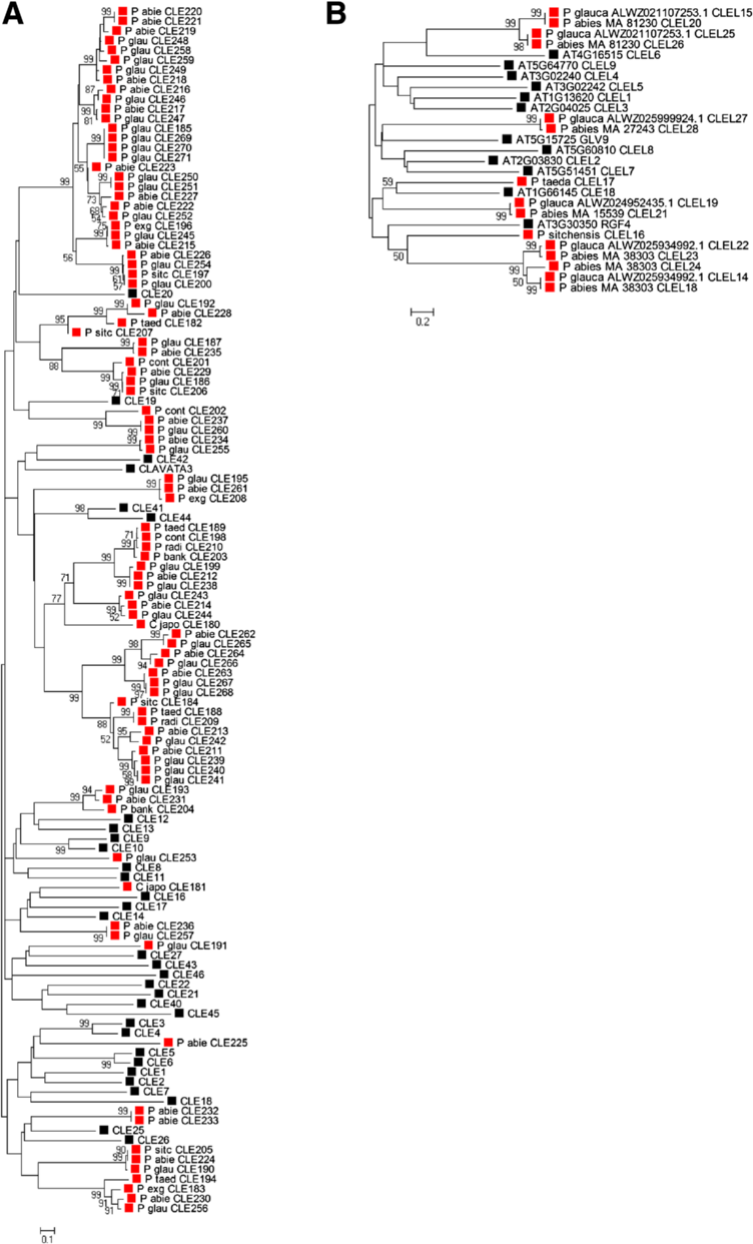
**Phylogenetic analysis of *Arabidopsis *and Pinophyta CLE and CLEL proteins.** A 1000-iteration Neighbour-Joining analysis using the Poisson correction method with alignment gaps and missing data eliminated only in pairwise sequence comparisons was used to create bootstrap consensus trees representing the putative phylogenetic relationships among the CLE and CLEL proteins between *Arabidopsis thaliana* and the Pinophyta species. The trees are drawn to scale, with branch lengths in the units of the number of amino acid substitutions per site. *Arabidopsis* proteins are represented by *black squares* and Pinophyta proteins are represented by *red squares*. **A**. CLE proteins; 194 positions in the final dataset. **B**. CLEL proteins; 277 positions in the final dataset.

**Figure 5:**
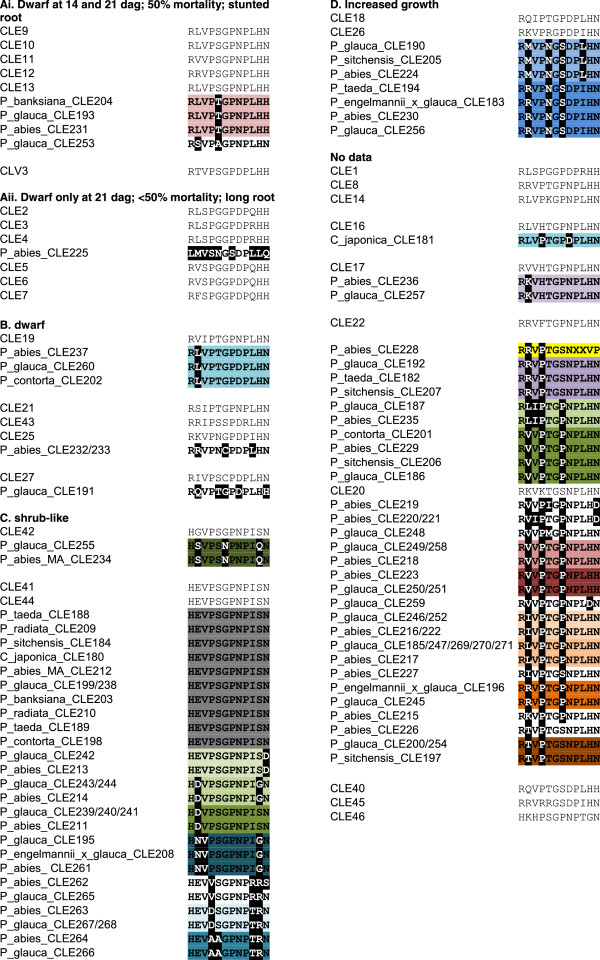
**Multiple alignment of predicted Pinophyta CLE dodecapeptide amino acid sequences.** Sequences are arranged by the phenotypic classifications assigned to *Arabidopsis* CLE proteins in [4], with conifer sequences grouped with their closest *Arabidopsis* homologue, as depicted in Figure 4A. Closest matching *Arabidopsis*-Pinophyta homologues are indicated by *boxes* and *light grey highlighting* over the protein names*.* The perfectly matching predicted CLE peptides between *Arabidopsis* and the Pinophyta are indicated by *dark grey highlighting* over the Pinophyta sequences. Mismatches in the Pinophyta sequences from their closest *Arabidopsis* homologue are indicated by *black highlighting* and *inverse lettering.* Perfectly matching CLE peptide sequences amongst the Pinophyta species are indicated by highlighting of *various colours*.

We examined the phylogenetic relationships to *Arabidopsis* among the conifer CLE and CLEL precursor protein sequences as a first attempt to assess potential protein role(s) and/or function(s). A 1000-iteration Neighbour-Joining analysis grouped the conifer protein sequences with varying degrees of phylogenetic distance from the *Arabidopsis* CLE and CLEL clades (Figure 4). In particular, a large clade of 30 protein sequences was grouped with *Arabidopsis* CLE41 and CLE42 proteins and 39 other conifer proteins were grouped with *Arabidopsis* CLE20 (Figures 4A and 5). Among the CLEL proteins, the closest *Arabidopsis* - Pinophyta evolutionary relationship was seen with *P. glauca* CLEL16 and *Arabidopsis* RGF4 (Figure 4B). CLEL17 grouped with CLE18, but this relationship may be spurious, as CLEL17 is only a partial protein sequence.

To assess potential functionality of and/or roles in plant growth and development by the conifer CLE proteins, we directly compared the CLE peptide sequences to the 32 *Arabidopsis* CLE peptide sequences, sorted by the *Arabidopsis* gene overexpression phenotypes as described by Strabala et al. [4]. We grouped the conifer peptide sequences with their most closely related protein(s) as inferred from the Neighbour-Joining analysis in Figure 4A. This comparison shows that the conifer CLE peptides are in general quite closely related to their predicted *Arabidopsis* counterparts. Moreover, there are many examples of perfect sequence conservation of CLE peptide sequences amongst the conifer species, even across genera (Figure 5) such that the conifer CLE gene contigs can be further grouped to 36 unique predicted CLE peptide sequences (Figure 5). Interestingly, with one exception, none of the known conifer CLE peptides is perfectly conserved with an *Arabidopsis* peptide (Figure 5). The sole exception is the finding that ESTs encoding perfectly conserved CLE41/44-TDIF peptide sequences were found in every conifer species examined (Figure 5).

### Synthetic CLE peptides exert developmental effects on pine seedlings

**Figure 6:**
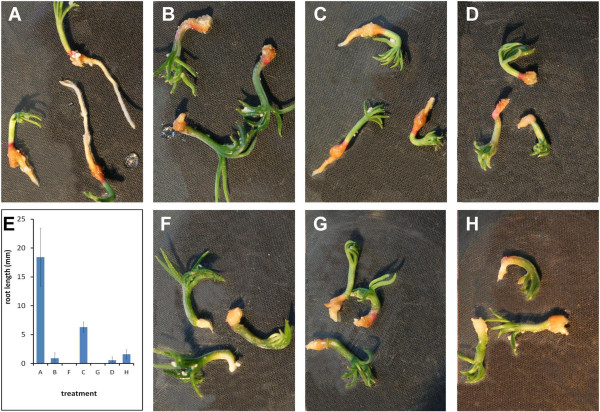
**Synthetic CLE peptide treatments of *Pinus radiata *seedlings.** Seedlings were either mock-inoculated (water, no peptide) **(A)**, CLE13 **(B,F)**, CLE41 **(C,G)**, or both CLE13 and CLE41 combined **(D,H)**. Peptide inoculations were with 10 μM (1X) peptide(s) **(B,C,D)**, or 100 μM (10X) **(F,G,H)** peptide(s). **E**. Plot of average root lengths observed in **A**-**D**, **F**-**H**, with corresponding lettering. Error bars represent the standard deviations.

To begin to assess whether the close sequence conservation of the predicted CLE peptides between *Arabidopsis* and the Pinophyta conferred similar phenotypic effects on pine seedlings to those observed in *Arabidopsis*, we applied two synthetic CLE peptides, CLE13 and CLE41/44-TDIF to *in vitro-*germinated *Pinus radiata* zygotic embryos. These two peptides were chosen since they were either identical (CLE41/44), or differing by only one amino acid (CLE13) from predicted conifer CLE peptides (Figure 5). Additionally, these peptides belong to important CLE subfamilies that exert opposite effects on root growth, yet have been demonstrated to exert synergistic effects on the development of vascular tissue in *Arabidopsis*[28]. As in *Arabidopsis* seedlings, the CLE13 peptide inhibited root elongation at concentrations as low as 10 μM (Figure 6B,E,F). CLE41/44-TDIF also inhibited root elongation in germinated pine zygotic embryos, and its effect was indistinguishable from CLE13 at 100 μM concentration (Figure 6C,E,G). Combining the CLE13 and CLE41 peptides resulted in essentially the same effect as application of CLE13 alone, although some root elongation was observed in the 100 μM dual application (Figure 6D,E,H). No reproducible effect on vascular tissue either in the root or the shoot was observed in these plants (data not shown).

### Molecular cloning and sequence analysis of *Pinus radiata* orthologues of *CLE188* and *CLE189*

We utilised the high degree of sequence conservation amongst pine species to design PCR primers based on the *P. taeda CLE41/44-TDIF* gene (*CLE188* and *CLE189*) sequences for amplification and molecular cloning of presumed orthologous coding sequences from *Pinus radiata* genomic DNA. As expected, these primers readily amplified the putative *CLE188* and *CLE189* orthologues from *Pinus radiata* (which we named *CLE209* and *CLE210*), which were 100% and 99.3% identical to the *P. taeda* sequences at the nucleotide level, respectively (data not shown), and 100% (CLE209) and 98.96% identical (CLE210) at the amino acid sequence level (Figure 1). This analysis revealed that, as expected, *CLE209* and *CLE210* contain no introns, at least not in their protein-coding segments (data not shown).

### Expression of the native *CLE209/210* genes *in planta* and in cultured cells

**Figure 7:**
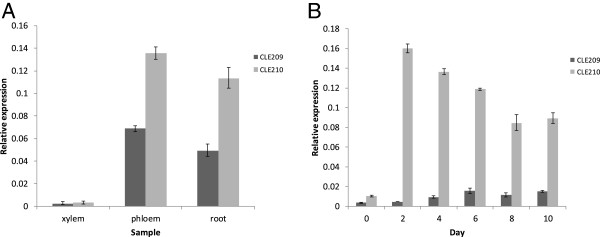
**Expression of CLE209 and CLE210 *in planta *in xylem, phloem, root and differentiating pine tracheary element cultured cells. A**. and **B**. Quantitative PCR analysis of *CLE209* (*dark grey bars*) and *CLE210* (*light grey bars*). **A**. Expression levels of *CLE209* and *CLE210* in xylem, phloem and root cells. **B**. Timecourse of expression levels of *CLE209* and *CLE210* in the *in vitro*-cultured *P.radiata* tracheary element differentiation system. Error bars represent standard deviations among three biological replicates. Student’s *t*-test analysis of the data revealed that all expression differences were significant to a 95% confidence interval except for the *CLE209*/*CLE210* expression (xylem samples), for Day 4/Day 8 and Day 6/Day 10 (*CLE209* expression in the tracheary element differentiation experiments) and for Day 8/Day 10 (*CLE210* expression in the tracheary element differentiation experiments).

Due to the perfect sequence conservation between the CLE41/44 and CLE209/210 peptides, we sought to verify experimentally whether the phloem-specific expression localisation of the CLE41/44 genes [9] was also conserved in *P. radiata.* To test this hypothesis, we isolated total RNA from developing xylem, developing phloem and whole roots and performed qPCR experiments with primers specific for *CLE209* and *CLE210*. As expected, expression of *CLE209/210* in stems was specific to developing phloem, with very low, if any, expression in xylem cells (Figure 7A). Similar to the relative expression of *CLE41* to *CLE44* in *Arabidopsis* inflorescence stems [4], expression of *CLE210* was approximately twice that of *CLE209* in both phloem and root (Figure 7A). In the pine tracheary element (TE) system [29], *CLE210* was at its highest abundance (~16-fold over basal expression level) in the early part (day 2) of the differentiation process, and its expression levels gradually declined to about 8-fold over basal expression levels as the number of differentiated TEs increased (Figure 7B). In contrast, CLE209 was only induced about 5-fold over a much lower basal expression level relative to CLE210 such that CLE210 mRNA was ~37-fold more abundant than CLE209 at day 2 but only ~6-fold more abundant at day 10. In contrast to CLE210, the expression level of CLE209 peaked only at day 6 and apparently remained steady thereafter (Figure 7B).

## Discussion

The *CLE* genes and peptides in plants are ancient and with a *CLE* gene found in the genome of the bryophyte moss *Physcomitrella patens*, the *CLE* genes are distributed throughout the plant kingdom and date back more than 450 million years in plant evolutionary history [30]. This sequence conservation is likely consistent with the multiple fundamental roles that CLE peptides play in plant development. Despite such sequence conservation, *CLE* genes had not been described to date in any conifer species. This is also the case for the *CLEL* genes, although this appears to be a much smaller gene family and these genes were much more recently identified and described [19-21]. Our meta-analysis of publicly available gymnosperm EST and genome sequence data revealed many *CLE* and *CLEL* genes in a variety of conifer species. With the exception of the presumed CLE41/44-TDIF orthologues, no predicted conifer CLE or CLEL peptide exhibited complete sequence conservation with any *Arabidopsis* CLE peptide (Figure 5). However, many predicted conifer CLE peptides are closely conserved with *Arabidopsis* CLE peptides (Figures 4 and 5) and this may suggest potential roles in conifer tree development.

Unlike most other species, analysis of the *P. glauca* and *P. abies* genome sequences revealed a large number of apparent paralogous genes, presumably arising from gene duplication events, encoding essentially perfectly conserved CLE proteins. Given the draft status of these genome sequences, it is unclear whether these genes are true paralogues, or simply genome assembly artefacts. However, one *P. abies* scaffold encoding the identical proteins CLE232 and CLE233, suggests that at least some of these identical or nearly identical genes may in fact also be paralogous. Closure of scaffold gaps will be required to verify these genes.

When intraspecies and interspecies conservation of predicted CLE peptides is taken into account, 36 unique CLE dodecamer sequences are observed (Figure 5). These 36 unique sequences are comparable with the 32 *CLE* genes and 30 unique CLE dodecamer peptide sequences found in *Arabidopsis*. It is interesting to note that despite the high degree of sequence similarity found between the *P. abies* and *P. glauca* genomes, there is currently not complete overlap among their *CLE* gene sequences. *CLE* gene structure in spruce appears to parallel that of *Arabidopsis*, with all but two genes (CLE261 and CLE272 and its presumed *P. glauca* orthologue CLE191) lacking introns. Due to the draft status of the two spruce genomes, it is currently unclear whether these are the only *CLE* genes with introns. As with the *CLEL* genes, other strong matches to the CLE domains were identified in the *P. glauca* and *P. abies* genomes, but the sequences were generally near to the ends of scaffolds, so gene structure predictions could not be made. Future builds of these draft genomes with additional sequence data will likely result in the identification of additional *CLE* and *CLEL* genes and reveal potential orthologous genes that cannot currently be unambiguously identified.

As in *Arabidopsis*, it appears that the *CLEL* gene family comprises fewer genes than the *CLE* gene family. Like most *Arabidopsis CLEL* genes, all the predicted Pinophyta *CLEL* genes contain introns (Figure 3). Although alternative splicing of *Arabidopsis CLEL* genes has been observed, these splice events do not affect the sequence of the active peptides encoded by these genes (data not shown). Unlike the known *CLEL* genes, it appears that at least some of the *CLEL* genes in the Pinophyta are alternatively spliced to transcripts that encode proteins with different putative CLEL peptide active sequences from exons separated from each other by ~1 kilobase pair (Figure 3, Additional file 4: Figure S3, Additional file 3: Table S2). While alternative splicing events leading to slightly different isoforms of peptide ligands such as ghrelin [31] or systemin [32] have been reported, to our knowledge, this splicing of distinct, widely separated alternative exons is a unique phenomenon with regard to peptide ligands. This phenomenon in turn suggests the potential for novel mechanisms of the regulation of *CLEL* expression in the Pinophyta that are not known to exist in other plant species. One such mechanism might be that the alternative *CLEL* transcripts are mutually exclusively produced in different tissue and/or cell types. Another mechanism might be the dynamic alterations of the ratio of the alternative transcripts within a cell, tissue or organ to “fine tune” a physiological or developmental process. It is conceivable that both such mechanisms could be occurring simultaneously. Regardless, these alternatively spliced *CLEL* forms suggest a previously unsuspected degree of dynamism in conifer signal transduction pathways.

Despite the ancient date of divergence of angiosperms from gymnosperms, estimated at 270-300 million years ago [33], conifers and dicotyledonous angiosperms still share certain characteristics not shared between the more recently diverged dicotyledon and monocotyledon angiosperm lineages. A particularly salient characteristic is the shared capacity for secondary growth between conifers and dicotyledons, which is the basis for wood formation [34,35]. Monocot species lack this capacity and achieve thickening of the stem via other mechanisms [36]. Although significant inroads have been made in understanding the regulation of secondary growth at the molecular level in dicots (particularly *Arabidopsis*), far less is understood about these processes in conifers. Therefore, the discovery of genes encoding perfectly conserved CLE41/44-TDIF peptide orthologues in all the Pinophyta species that are known to have *CLE* genes is strongly suggestive of a conserved role between conifers and dicots for these peptides in the regulation of vascular cambium homeostasis. This hypothesis is all the more compelling considering that there is essentially no other sequence conservation between these *Arabidopsis* and conifer gene sequences (data not shown), suggesting very strong selective pressure for the conservation of the CLE41/44-TDIF peptide sequence among species with a vascular cambium. Consistent with such a hypothesis, all the conifer CLE41/44-TDIF ESTs we identified in our EST database meta-analysis were sourced from RNA isolated from inner bark and/or phloem or root tissues (Additional file 3: Table S1). The bioinformatic meta-analysis was confirmed by our *P. radiata* qPCR results that showed phloem-specific expression in the stem, as well as expression in root (Figure 7A). This expression pattern is also consistent with that of the presumed *Arabidopsis* orthologues, *CLE41* and *CLE44*[9]. Therefore, it seems possible that the CLE41/44-TDIF genes in conifers are playing similar roles in the regulation of secondary growth to those in dicot species. This apparent conservation of a key component of the mechanism of vascular cambium homeostasis between dicot angiosperms and gymnosperms may be an indicator of the inherent capacity of these clades to make wood. Indeed, the natural variation in lignin content, neutral monosaccharide content, microfibril angle and biomechanical properties in *Arabidopsis* inflorescence stems showed correlations that were consistent with correlations in many of these traits in woody species [37]. Strikingly, the CLE41/44-TDIF peptide motif is only known to be conserved in only one monocot species, the date palm, *Phoenix dactylifera*[35], which undergoes stem thickening, although via a different mechanism that is less well understood than that of woody plants.

It is well-established that CLE41/44-TDIF is an inhibitor of *in vitro* TE differentiation [13] as well as xylem differentiation [9]. Therefore, the observation that the expression of the likely pine orthologues of the CLE41/44 genes are in fact apparently upregulated upon induction of pine TE differentiation (Figure 7B), seems initially counterintuitive. However, in *Arabidopsis* plants the *CLE41/44* genes are only expressed in differentiated phloem cells [9]. Thus, in the *P. radiata* TE differentiation system [38], which presumably initially comprises dedifferentiated and/or undifferentiated cells, the expression of *CLE41/44-TDIF* would not be expected prior to initiation of differentiation (Figure 7). Since it is now clear that differentiated phloem cells provide developmental cues to the vascular cambium in the form of CLE41/44-TDIF to suppress xylem differentiation [9], expression of CLE41/44-TDIF is thus a specific marker for phloem cells. Therefore, the strong induction of the CLE41/44-TDIF orthologues *CLE209/210* in the *P. radiata* TE system (Figure 7) indicates that the TE differentiation is accompanied by the differentiation and development of phloem or phloem-like cells and thus this *in vitro* system very closely parallels vascular development *in planta*. Hirakawa et al. [39] demonstrated a role for CLE41/44-TDIF in stimulating the proliferation of procambial cells, the cell type in which the CLE41/44-TDIF receptor, PXY, is found [9]. Thus, only upon induction of differentiation of tracheary elements is the presence of the CLE41/44-TDIF peptide required, as some non-TE cells must exist to provide signals to the cells that eventually differentiate into TEs [40] and CLE41/44-TDIF is required to maintain this undifferentiated state. Thus, the so-called tracheary element differentiation system may also be thought of as a phloem/procambium differentiation system as well.

*CLE* gene overexpression and synthetic peptide application have been used extensively to characterise CLE functions *in planta.* We wished to examine the effects of CLE family members that have synergistic effects on vascular development in *Arabidopsis* to determine if such relationships hold in conifers. We were unable to observe any effects on vascular development in freshly germinated *P.radiata* seedlings because, unlike *Arabidopsis*, extended periods in liquid medium are not tolerated well by this species (M. West and T. Strabala, unpublished observations), leading to artefacts that obscured any effects on vascular development. However, we did find that both CLE13 and CLE41/44-TDIF peptides inhibited root development when applied to pine seedlings with some solid support, to prevent the submergence of the seedlings. CLE13, a potent inhibitor of root elongation in both *Arabidopsis* and rice [41] was more effective than CLE41/44-TDIF in this regard. Interestingly, with application of both peptides, inhibition of root elongation appeared not to be as strong as CLE13 alone, either at 10 or 100 μM, or CLE41 alone at 100 μM (Figure 6), so there may be some synergistic interactions between these peptides in pine as well.

It was somewhat unexpected that CLE41 peptide inhibited root development in pine at all, since experiments in *Arabidopsis* have shown that has no effect on root elongation either when overexpressed [4], or when exogenously applied [13,41]. However, Kinoshita et al. [41] demonstrated that CLE41/44 had a mild inhibitory effect on root elongation in rice when applied to roots at a 1 μM concentration. Presumably, this inhibition would have been greater at a 10 μM concentration (which was the lowest concentration we used on the pine seedlings), so it appears that this root response to exogenous CLE41/44 is shared between pine and rice. The basis for this shared response is not yet clear. CLE41/44 is not an endogenous peptide in rice as it is in conifers and dicots. Despite the conservation of the CLE41/44 peptide in conifers, *P. radiata* is substantially evolutionarily diverged from *A. thaliana*. Additionally, such experiments provide CLE peptides at significantly higher concentrations than is found *in vivo* (and likely above the dissociation constants of many non-cognate receptors). This situation likely causes CLE peptides to bind to receptors that they would not normally bind, resulting in neomorphic or antimorphic phenotypes [42]. Although it appears that CLE41/44-TDIF phloem-specific expression in *Arabidopsis* is conserved [9], which implies a putative PXY receptor orthologue in pine, the ectopic responses of other receptors resulting from interaction with CLE41/44-TDIF may not be the same as *Arabidopsis* in all cases.

## Conclusions

The CLE and CLEL peptide ligand families are well known to play many important roles in angiosperm plant growth and development. Conifer and dicot angiosperm taxa share certain growth characteristics, most notably a vascular cambium, not shared by monocot angiosperms, yet they differ fundamentally in many other aspects of their growth and development. We show that *CLE* and *CLEL* genes are found in the Pinophyta with gene numbers and sequence diversity similar to angiosperms, yet their active peptide sequences are not perfectly conserved, with one exception, the conserved CLE41/44-TDIF peptide. Our experiments involving this peptide and *P. radiata* orthologues of the genes encoding are suggestive that they play orthologous roles in vascular development among conifer and dicot species. Conversely, we provide evidence that at least some *CLEL* genes appear to be regulated in completely different ways than their angiosperm counterparts, via splicing of alternative exons that encode different CLEL peptides. The substantial sequence differences between these alternate peptides suggest that they either bind different receptors, or if they interact with the same receptor, they do so with different affinities and/or binding sites. Although alternative transcript splicing is a thoroughly studied phenomenon, to our knowledge, this is a completely novel means to regulate the expression of peptide signalling ligands. Further comparative analysis of these signalling ligand gene families in conifers and dicot angiosperms will surely lead to deeper understanding of growth and developmental processes in both of these major phylogenetic clades and our ability to manipulate these processes for more sustainable wood and wood product production.

## Methods

### Bioinformatic analysis

TBLASTN searches, using the NCBI-hosted BLAST search tool (http://blast.ncbi.nlm.nih.gov/Blast.cgi) were conducted. Each known *Arabidopsis thaliana CLE* and *CLEL* gene was used as a query sequence against the NCBI EST DNA Spermatophyta (seed plants) database (NCBI taxid 58024), excluding the Magnoliophyta (NCBI taxid 3398) to allow searching of all gymnosperm species, were performed. Sequence hits were then further analysed using GAP4 [43] to sort the hits into contigs, combined with manual editing with particular attention to species of origin due to high levels of sequence conservation across species with several of the *CLE* genes. The consensus sequences from all validated contigs were used as query sequences in a second round of TBLASTN analysis of the Pinophyta database subset to identify sequences that were not initially identified in the original TBLASTN searches, both to extend the contigs and to ensure that no *CLE* or *CLEL* sequences were overlooked due to truncated sequences lacking a *CLE* or *CLEL* domain. These sequences were also used to query the *Picea abies* and *Picea glauca* genome sequence V1.0 assemblies [25,26], to search for genes that might not have been detected in EST databases.

In the case of EST contigs, protein sequences were assumed to be full-length if the use of the 5′-most predicted in-frame Met residue of the CLE or CLEL contig predicted amino acid sequence yielded a signal peptide. If a signal peptide was not identified at this stage, the contig was assumed not to be full-length. If the contig was of a *P. glauca* sequence*,* then a full-length genomic sequence was sought. Putative CLE genes were selected from genomic sequences on the basis of having a hit to the CLE motif query sequence, plus an open reading frame with at least one met residue as an initiator codon and a downstream predicted signal peptide. Protein sequences were analysed for signal peptide sequences using the SignalP 4.1 server [27]. In the case of genomic sequences, the lack of a signal peptide was interpreted to mean that the initial CLE motif hit was likely to be spurious and the sequence was not examined further. Multiple sequence alignments and phylogenetic trees were generated using MEGA, version 4.0.2 (http://www.megasoftware.net/mega4/mega.html) [44].

### CLE peptide treatments of pine embryos

Synthetic peptides, obtained from Auspep (Parkville, Australia), were dissolved in 50 mM sodium phosphate buffer (pH 6.0) and stored at ^-^80°C. *Pinus radiata* zygotic embryos were grown under sterile conditions for 13d in 50 ml Falcon tubes containing 1 g of perlite and 4 ml of KNV medium [45]. Plants were grown 16 h/25°C day (80 μE m^-2^ s^-1^ light intensity) and 8 h/18°C night. CLE peptide-treated embryos were grown as for the negative controls with either added CLE13 (H-Arg-Leu-Val-Hyp-Ser-Gly-Hyp-Asn-Pro-Leu-His-His-OH) or CLE41 (H-His-Glu-Val-Hyp-Ser-Gly-Hyp-Asn-Pro-Ile-Ser-Asn-OH) (Hyp = hydroxyproline) at either 10 μM (1X) or 100 μM (10X) final concentration.

### Nucleic acid extractions from pine tissues

Genomic DNA was extracted from *P. radiata* embryogenic callus tissue essentially as described [46]. Total RNA was extracted from *P. radiata* induced xylogenic callus material at 0, 2, 4, 6, 8 and 10 days post-induction [29] or from uninduced callus material at equivalent time points using Purelink® Plant RNA reagent (Ambion, Life Technologies) as per the manufacturer’s instructions.

For extraction of total RNA from *P. radiata* xylem, phloem and root, tissue samples constituting early season (spring) vascular cambium formation were collected from a two-year-old glasshouse-grown tree. Bark was peeled from most of the stem and developing xylem scrapings were taken along the length of the stem, avoiding tissue near branch whorls. Phloem tissue was carefully cut into sections from the inner surface of the bark peelings. Root samples were excised and quickly washed in phosphate buffer to rinse off potting mix. All tissue samples were snap-frozen in liquid nitrogen and stored at -80°C prior to RNA extraction. Approximately 0.5 g – 1.0 g of frozen tissue was ground to a fine powder with a mortar and pestle under liquid nitrogen and quickly transferred to a 50 ml tube containing 10 ml of CTAB extraction buffer (2% CTAB, 2% PVP-40, 2.0 M NaCl, 100 mM Tris-HCl pH8.0, 25 mM EDTA pH8.0) to which 2% β-mercaptoethanol had been freshly added, preheated to 65°C. Samples were incubated for 30 min at 65°C, with occasional mixing by inversion, then extracted twice with 10 ml of chloroform/isoamyl alcohol (24:1 v/v), mixed by careful inversion for at least 5 min, then centrifuged at 9500 × g, 4°C for 10 min. Aqueous supernatants were transferred to clean 15 ml tubes and ¼ volumes of 10 M LiCl were added to each tube, mixed by careful inversion and precipitated overnight at 4°C. Precipitates were pelleted by centrifugation at 13000 × g, 4°C for 30 min. Supernatants were decanted and the RNA pellets were redissolved in 1 ml STE buffer (1 M NaCl, 10 mM Tris-HCl pH8.0, 1 mM EDTA pH8.0), maintaining a temperature of 0°C throughout. RNA was reprecipitated by addition of 2× volumes of absolute ethanol (-20°C). Precipitates were pelleted by centrifugation as before, washed with 1× volume of 70% ethanol and pelleted again as before. RNA pellets were air-dried and resuspended in 200–300 μl 10 mM Tris-HCl pH8.0, depending on apparent yield, and stored at -80°C.

### Quantitative RT-PCR of *P. radiata* total RNA

Messenger RNA was isolated from total RNA using Dynabeads® oligo (dT)_25_ (Ambion, Life Technologies) following the manufacturer’s instructions. First-strand cDNA was synthesised from the mRNA using a qScript™ Flex cDNA synthesis kit (Quanta Biosciences) and priming with the oligo-dT included. The cDNA was quantified on a fluorometer using Quant-iT™ Oligreen ssDNA reagent (Molecular Probes, Life Technologies). Real-time PCR was performed on a LightCycler® 1.5 (Roche) using a LightCycler® FastStart DNA Master^PLUS^ SYBR Green I kit as previously described [47], with the PCR reaction volume scaled down to 10 μl. Statistical significance of differences in expression levels between samples was determined using Student’s *t-*test [48].

### Molecular cloning of *CLE209* and *CLE210*

EST sequences from *Pinus taeda*, GenBank/EMBL/DDBJ accession numbers CO365940 and DR744109 were used to design primer sequences for the amplification of the orthologous genes from *Pinus radiata* using the following primer pairs:

5′-GCTCTAGAATGGCAGATGCTTTAGTGGAT-3′ and

5′-GCGCGGCCGCTCAATTTGATATTGGATTTGGACCG-3′ (*CLE188*);

5′-GCTCTAGAATGGCGGATGGTTTTGTT-3′ and

5′-GCGCGGCCGCTTACCTATTACTAATTGGATTTGGAC-3′ (*CLE189*)

Xba I and Not I restriction enzyme recognition sequences were incorporated in the 5′ ends of forward and reverse primers respectively to facilitate directional cloning. Gene sequences were amplified in a total volume of 50 μL using 50 ng of genomic DNA, 2.5 U of Roche Expand High Fidelity^PLUS^ DNA polymerase, 2.5 mM MgCl_2_, 300 μM dNTPs and 300 nM of each primer. PCR parameters were: initial denaturation at 94°C for 2 mins, followed by 30 cycles of 94°C for 30s, 50°C for 30s and 72°C for 1 min, a final extension of 72°C for 5 min. Completed reactions were held at 10°C.

## Abbreviations

BLAST: Basic local alignment search tool; CLE: Clavata/embryo-surrounding region (gene or protein); CLE41/44-TDIF: CLE41/44-tracheary element differentiation factor; CLEL: CLE – Like (gene or protein); EST: Expressed sequence tag; NCS: Non-conserved sequence; PSK: Phytosulphokine (gene or protein); PSY1: Plant peptide containing sulphated tyrosine 1 (gene or protein); RAM: Root apical meristem; RLK: Receptor-like kinase; SAM: Shoot apical meristem.

## Competing interests

The authors declare that they have no competing interests.

## Authors’ contributions

TJS conceived the study, conducted the bioinformatic analysis and drafted the manuscript. LP did the RNA purifications, the qPCR analyses, part of the cloning and participated in the drafting of the manuscript. MW did the CLE peptide application experiments and participated in the drafting of the manuscript and LS did part of the cloning and participated in the drafting of the manuscript. All authors read and approved the final manuscript.

## Supplementary Material

Additional file 1: Figure S1 Contig analysis of putative conifer CLE gene ESTs. Putative CLE gene ESTs were identified, and contig alignments and assignments were performed as described in Methods. Putative signal peptide analysis was conducted using the SignalP 4.1 server (Technical University of Denmark), respectively. Predicted open reading frames are highlighted in *turquoise*, except for the putative CLE peptide sequences, which are highlighted in *yellow*. Putative signal peptide cleavage sites are denoted by *arrowheads*. Potential in-frame ribosome initiation codons consistent with a signal peptide are highlighted in *teal*. Click here for file

Additional file 2: Figure S2 Contig analysis of putative conifer *CLEL* gene ESTs. Putative CLEL gene ESTs were identified, and contig alignments and assignments were performed as described in Methods. Putative signal peptide analysis was conducted as described in **Figure S1** Predicted open reading frames are highlighted in *turquoise*, except for the putative CLE peptide sequences, which are highlighted in *yellow*. Putative signal peptide cleavage sites are denoted by *arrowheads*. Potential in-frame ribosome initiation codons consistent with a signal peptide are highlighted in *teal*. Click here for file

Additional file 3: Table S1 CLE genes and proteins in the Pinophyta. Excel file, “Strabala et al **Tables S1** + **S2**”; tab labelled “**Table S1** – CLE genes”. **Table S2**. CLEL genes and proteins in the Pinophyta. Excel file, “Strabala et al **Tables S1 + S2**”; tab labelled “**Table S2** – CLEL genes”. Click here for file

Additional file 4: Figure S3 Multiple alignment of predicted Pinophyta CLEL peptide amino acid sequences. Sequences are arranged as depicted in Figure 4B with conifer sequences grouped with their closest *Arabidopsis* homologue. Closest matching *Arabidopsis*-Pinophyta homologues are positioned directly beneath their putative closest *Arabidopsis* homologue *Arabidopsis* gene names are signified with *grey highlighting.* Mismatches in the Pinophyta sequences from their closest *Arabidopsis* homologues are indicated by *black highlighting* and *inverse lettering.* Click here for file
