# Supplementary material for: Bioinformatic and phylogenetic analysis of the CLAVATA3/EMBRYO-SURROUNDING REGION (CLE) and the CLE-LIKE signal peptide genes in the Pinophyta
Source: BMC Plant Biol. 2014 Feb 14;14:47. doi: 10.1186/1471-2229-14-47 (PMC4016512; doi:10.1186/1471-2229-14-47)
Supplement: Additional file 1: Figure S1 — Contig analysis of putative conifer CLE gene ESTs. Putative CLE gene ESTs were identified, and contig alignments and assignments were performed as described in Methods. Putative signal peptide analysis was conducted using the SignalP 4.1 server (Technical University of Denmark), respectively. Predicted open reading frames are highlighted in turquoise, except for the putative CLE peptide sequences, which are highlighted in yellow. Putative signal peptide cleavage sites are denoted by arrowheads. Potential in-frame ribosome initiation codons consistent with a signal peptide are highlighted in teal. [file 1471-2229-14-47-S1.docx]

**Fig. S1. Contig analysis of putative conifer *CLE* gene ESTs.** Putative CLE gene ESTs were identified, and contig alignments and assignments were performed as described in *Methods*. Putative signal peptide analysis was conducted using the SignalP 4.1 server (Technical University of Denmark), respectively. Predicted open reading frames are highlighted in *turquoise*, except for the putative CLE peptide sequences, which are highlighted in *yellow*. Putative signal peptide cleavage sites are denoted by *arrowheads*. Potential in-frame ribosome initiation codons consistent with a signal peptide are highlighted in *teal*.

1. ***Cryptomeria japonica CLE180***

10 20 30 40 50

+4 BY881512-1_Cjaponica ..................................................

+1 AU084295-1_Cjaponica ...................................

CONSENSUS CGGGGTTCTAATTATGTTTTTGTTGGGCTTATTAATGCTTCAGCCCTCAG

+ Frame 2 G V L I M F L L G L L M L Q P S

60 70 80 90 100

+4 BY881512-1_Cjaponica ..................................................

+1 AU084295-1_Cjaponica ..................................................

-2 BP174244-1_Cjaponica .........

CONSENSUS ATCCTGCACCATGTGGAAGGAGAAAACCAAACACACCAGCTTTGCCACAA

+ Frame 2 D P A P C G R R K P N T P A L P Q

110 120 130 140 150

+4 BY881512-1_Cjaponica ..................................................

+1 AU084295-1_Cjaponica ..................................................

-2 BP174244-1_Cjaponica ..................................................

CONSENSUS CAGCAACAGGTACATGTTATTTCAAGGTATCTGTTGTCAACCTCCAATCA

+ Frame 2 Q Q Q V H V I S R Y L L S T S N Q

160 170 180 190 200

+4 BY881512-1_Cjaponica ..................................................

+1 AU084295-1_Cjaponica ..................................................

-2 BP174244-1_Cjaponica ..................................................

CONSENSUS GTTTAATGTTCCCAAGCATTCTAATAAGCCTTTCAGAGGTTATAATGCTA

+ Frame 2 F N V P K H S N K P F R G Y N A

210 220 230 240 250

+4 BY881512-1_Cjaponica ..................................................

+1 AU084295-1_Cjaponica ..................................................

-2 BP174244-1_Cjaponica ..................................................

CONSENSUS GTGCCCATGAAGTTCCAAGTGGTCCAAATCCAATATCTAATTAGAAATAT

+ Frame 2 S A H E V P S G P N P I S N * K Y

260 270 280 290 300

+4 BY881512-1_Cjaponica ..................................................

+1 AU084295-1_Cjaponica ..................................................

-2 BP174244-1_Cjaponica ..................................................

CONSENSUS CACAGTTATACTATTCATTATGATCTAATATGTTAATGATGAGAATGAGA

+ Frame 2 H S Y T I H Y D L I C * * * E * E

310 320 330 340 350

+4 BY881512-1_Cjaponica ..................................................

+1 AU084295-1_Cjaponica ..................................................

-2 BP174244-1_Cjaponica ..................................................

CONSENSUS GGATAGGGATGAAGATGATTGAAGAGAGGGACAATCATGATCCATCTTTC

+ Frame 2 D R D E D D * R E G Q S * S I F

360 370 380 390 400

+4 BY881512-1_Cjaponica ..................................................

+1 AU084295-1_Cjaponica ..................................................

-2 BP174244-1_Cjaponica ..................................................

CONSENSUS AATTTTGAATTCAGAATTGGAAATGGGTATCTGTTCAAATCAATGGCAGA

+ Frame 2 Q F * I Q N W K W V S V Q I N G R

410 420 430 440 450

+4 BY881512-1_Cjaponica ......................................

+1 AU084295-1_Cjaponica ..................................................

-2 BP174244-1_Cjaponica ..............................................S...

CONSENSUS AGCATCAGCAGCTTTTTTATGTTCACTTTAGCTGGTTTCGGATAAAGGGT

+ Frame 2 S I S S F F M F T L A G F G * R V

460 470 480 490 500

+1 AU084295-1_Cjaponica ..................................................

-2 BP174244-1_Cjaponica ......................Y...........................

CONSENSUS TCTATATTAGGGATCTTGGGTATAGTAAACTATTATTAAGGCTTATCAAA

+ Frame 2 L Y * G S W V * * T I I K A Y Q

510 520 530 540 550

+1 AU084295-1_Cjaponica .......

-2 BP174244-1_Cjaponica .......................................Y........Y.

CONSENSUS AGTCTGGCCATGGCAGTTTGCTTTCGGCACCGGCTATGA-ACGGTATA-A

+ Frame 2 K S G H G S L L S A P A M - R Y -

560 570 580

-2 BP174244-1_Cjaponica ...Y.................................

CONSENSUS TCT-TTCTTAACGATGTGTTTTGTTTACGGAGAAATA

+ Frame 2 S - L N D V F C L R R N

1. ***Cryptomeria japonica CLE181***

10 20 30 40 50

+5 BY892398-1_Cjaponica ..................................................

+6 BY909937-1_Cjaponica ..................................................

CONSENSUS GCTCAGGCAGTAGTGAAGCATGGTGAAATGCAAAAGTGCAGTTTGTAGGA

+ Frame 2 L R Q * * S M V K C K S A V C R

60 70 80 90 100

+5 BY892398-1_Cjaponica ..................................................

+6 BY909937-1_Cjaponica ..................................................

CONSENSUS TGATAGTTTTGTTTATTGTAGCCATCATAACTTGGGTGGGATTCTCATCA

+ Frame 2 M I V L F I V A I I T W V G F S S

110 120 130 140 150

+5 BY892398-1_Cjaponica ..................................................

+6 BY909937-1_Cjaponica ..................................................

CONSENSUS AGGCTATTGATAGAAACAAATGGAAAGCAACTTAGCAATTATTACCACTT

+ Frame 2 R L L I E T N G K Q L S N Y Y H F

160 170 180 190 200

+5 BY892398-1_Cjaponica ..................................................

+6 BY909937-1_Cjaponica ..................................................

CONSENSUS TGCAGGAGATAGACTTAGAGTTGTGGATGAGTTGAGTGAAAAGAAGAAAT

+ Frame 2 A G D R L R V V D E L S E K K K

210 220 230 240 250

+5 BY892398-1_Cjaponica ..................................................

+6 BY909937-1_Cjaponica ..................................................

CONSENSUS TAATGGAGGATTCTGATAATCATCATGGATGCAACTGTTATAAGGCAGCA

+ Frame 2 L M E D S D N H H G C N C Y K A A

260 270 280 290 300

+5 BY892398-1_Cjaponica ..................................................

+6 BY909937-1_Cjaponica ..................................................

CONSENSUS GAGGAAGAGAAGACAGATCCCATTTATGGAAATGATAAAAGGCTTGTACC

+ Frame 2 E E E K T D P I Y G N D K R L V P

310 320 330 340 350

+5 BY892398-1_Cjaponica ..................................................

+6 BY909937-1_Cjaponica ..................................................

CONSENSUS CACAGGTCCAGACCCTTTGCATAATAGGTAAGGCCTAGGATCTACAAGAA

+ Frame 2 T G P D P L H N R * G L G S T R

360 370 380 390 400

+5 BY892398-1_Cjaponica ..................................................

+6 BY909937-1_Cjaponica ..................................................

CONSENSUS TATGTATCAGTGATGAGCAGAGGGCTGCCTTCAAGAGAAATAGTCTTGTA

+ Frame 2 I C I S D E Q R A A F K R N S L V

410 420 430 440 450

+5 BY892398-1_Cjaponica ..................................................

+6 BY909937-1_Cjaponica ..................................................

CONSENSUS AGTAGGCATATACAAATGTTTGCTAACAGTTTCTATATAATTTTTGTATT

+ Frame 2 S R H I Q M F A N S F Y I I F V F

460 470 480 490 500

+5 BY892398-1_Cjaponica ..............

+6 BY909937-1_Cjaponica ..................................................

CONSENSUS CTTTTAAGTTATGCAAAACAAAACAAAACAAAATAAAGATTTGTCTATTT

+ Frame 2 F * V M Q N K T K Q N K D L S I

510

+6 BY909937-1_Cjaponica ................

CONSENSUS ATAGTTTGTAAACCAC

+ Frame 2 Y S L * T T

1. ***Pinus taeda CLE182***

10 20 30 40 50

+8 CF477586_Ptaeda ...................T..........G...................

+24 CV137762_Ptaeda ...............................

+41 DT626269_Ptaeda ...............................

+25 CX715848_Ptaeda ........*.............*....

CONSENSUS CGTTTTGACAATATTTGTTCCCCTCTTCTTCAGCCCCACGATTGCAAAAT

+ Frame 1 R F D N I C S P L L Q P H D C K I

60 70 80 90 100

+8 CF477586_Ptaeda ..................................................

+24 CV137762_Ptaeda ..................................................

+41 DT626269_Ptaeda ..................................................

+25 CX715848_Ptaeda ..................................................

CONSENSUS CTGGACTTTCGGATTCAGCTCGCATATCTGGTACGCCCCGCCGCGCCTCG

+ Frame 1 W T F G F S S H I W Y A P P R L

110 120 130 140 150

+8 CF477586_Ptaeda .....CCCCGCCCCGCCTCACCCCA.........................

+24 CV137762_Ptaeda ..................................................

+41 DT626269_Ptaeda ..................................................

+25 CX715848_Ptaeda ..................................................

CONSENSUS CCTCG********************CCCCGCCCCGCCTCACCCCACCCCG

+ Frame 1 A S P R P A S P H P

160 170 180 190 200

+8 CF477586_Ptaeda ..................................................

+24 CV137762_Ptaeda ..................................................

+41 DT626269_Ptaeda ..................................................

+25 CX715848_Ptaeda ..................................................

CONSENSUS CCTCTTGCTCGTAATTTCATAGGAGAATTGTTCATATCCAAGAGAATTGT

+ Frame 1 A S C S * F H R R I V H I Q E N C

210 220 230 240 250

+8 CF477586_Ptaeda ..................................................

+24 CV137762_Ptaeda ..................................................

+41 DT626269_Ptaeda ..................................................

+25 CX715848_Ptaeda ..................................................

CONSENSUS TGGTCGGGGTTTTGGACGGATTTGTTCGGATTCTTGTACAATTACGGTAG

+ Frame 1 W S G F W T D L F G F L Y N Y G R

260 270 280 290 300

+8 CF477586_Ptaeda .........................A........................

+24 CV137762_Ptaeda ..................................................

+41 DT626269_Ptaeda ..................................................

+25 CX715848_Ptaeda ..................................................

CONSENSUS GGGAATATATATTCTACAAGCCCAGTTTGTGTTCTGGTCCGTTTCAGAGA

+ Frame 1 G I Y I L Q A Q F V F W S V S E

310 320 330 340 350

+8 CF477586_Ptaeda ..................................................

+24 CV137762_Ptaeda ..................................................

+41 DT626269_Ptaeda ..................................................

+25 CX715848_Ptaeda ..................................................

CONSENSUS TTTCTGTGATGATCGATCGCTGGAGCAGATCAAGATTGAGCAACATGAAG

+ Frame 1 I S V M I D R W S R S R L S N M K

360 370 380 390 400

+8 CF477586_Ptaeda ..................................................

+24 CV137762_Ptaeda ..................................................

+41 DT626269_Ptaeda ..................................................

+25 CX715848_Ptaeda ..................................................

CONSENSUS CGTTTGTTCCTTTGTGTTTTGTTCGTGCTGTGGATTGTACTTTGCTCTGC

+ Frame 1 R L F L C V L F V L W I V L C S A

410 420 430 440 450

+8 CF477586_Ptaeda ..................................................

+24 CV137762_Ptaeda ..................................................

+41 DT626269_Ptaeda ..................................................

+25 CX715848_Ptaeda ..................................................

CONSENSUS CTCAAGGGCACCGCCTTCGACATTTTTCAATACACACGAGTCTACGGAGA

+ Frame 1 S R A P P S T F F N T H E S T E

460 470 480 490 500

+8 CF477586_Ptaeda ..................................................

+24 CV137762_Ptaeda ..................................................

+41 DT626269_Ptaeda ..................................................

+25 CX715848_Ptaeda ..................................................

CONSENSUS GCCCGAGCAGGTCGTCCGCTAATTTTCAGCCCTCTTTCAAACTGCCCCAT

+ Frame 1 S P S R S S A N F Q P S F K L P H

510 520 530 540 550

+8 CF477586_Ptaeda ....................C.............................

+24 CV137762_Ptaeda ..................................................

+41 DT626269_Ptaeda ..................................................

+25 CX715848_Ptaeda ..................................................

+7 CF477528_Ptaeda ...........C.............................

CONSENSUS CCTCCAACTGGACAGGTAGATGCAGGCAGATTCGGAGTGGATAAACGACG

+ Frame 1 P P T G Q V D A G R F G V D K R R

560 570 580 590 600

+8 CF477586_Ptaeda ..................................................

+24 CV137762_Ptaeda ..................................................

+41 DT626269_Ptaeda ..................................................

+25 CX715848_Ptaeda ..................................................

+7 CF477528_Ptaeda ..................................................

CONSENSUS AGTCCCCACGGGCTCCAACCCCTTGCACAACAGGTAGCCGGGACGCCCTA

+ Frame 1 V P T G S N P L H N R * P G R P

610 620 630 640 650

+8 CF477586_Ptaeda ..................................................

+24 CV137762_Ptaeda ..................................................

+41 DT626269_Ptaeda ..................................................

+25 CX715848_Ptaeda ............................................

+7 CF477528_Ptaeda ..................................................

CONSENSUS ATTGCCATATTAGTATGCCTACTTGTGCCTGCAACTTCAATCGCAAAGTT

+ Frame 1 N C H I S M P T C A C N F N R K V

660 670 680 690 700

+8 CF477586_Ptaeda ...........................................G......

+24 CV137762_Ptaeda ..................................................

+41 DT626269_Ptaeda ..................................................

+7 CF477528_Ptaeda ..................................................

CONSENSUS TCACAGCAAGAGAATTATAGATGTTTTTAATTCCTGATCTGCTAAATTAA

+ Frame 1 S Q Q E N Y R C F * F L I C * I N

710 720 730 740 750

+8 CF477586_Ptaeda ............

+24 CV137762_Ptaeda ..................................................

+41 DT626269_Ptaeda ..................................................

+7 CF477528_Ptaeda ..................................................

CONSENSUS TACTTTGGATATAGACAACTTAGGCAGAAATTAGAGTTGAATGAGAAGAA

+ Frame 1 T L D I D N L G R N * S * M R R

760 770 780 790 800

+24 CV137762_Ptaeda ..................................................

+41 DT626269_Ptaeda ..................................................

+7 CF477528_Ptaeda ..................................................

CONSENSUS AAAATTGCGCACAGACGGCAATAGAAAAATGTAAGCATATAGAGGATGTA

+ Frame 1 K N C A Q T A I E K C K H I E D V

810 820 830 840 850

+24 CV137762_Ptaeda ..................................................

+41 DT626269_Ptaeda ..................................................

+7 CF477528_Ptaeda ..................................................

CONSENSUS AGTAATACATGGTATTGTACGATGTGTGCTTGCATTTGACTACACAACGT

+ Frame 1 S N T W Y C T M C A C I * L H N V

860 870 880 890 900

+24 CV137762_Ptaeda ..................................................

+41 DT626269_Ptaeda ..................................................

+7 CF477528_Ptaeda ..................................................

CONSENSUS TACGCCGTCCGGCAGATCCATGTTTTATTTGCAGCATCATATTTTCATAT

+ Frame 1 T P S G R S M F Y L Q H H I F I

910 920 930 940 950

+24 CV137762_Ptaeda ..............................

+41 DT626269_Ptaeda ....................

+7 CF477528_Ptaeda ..................................................

CONSENSUS TTACGGGCTTTGCAGATCCCTTACGCTCCAAGGTGCAAGGTAAGGATAAT

+ Frame 1 F T G F A D P L R S K V Q G K D N

960 970 980 990 1000

+7 CF477528_Ptaeda ..................................................

CONSENSUS TTTTAGACCGTGCTTTCAAAGTTTTCGTAATCAGATTCCTGCTAGCTGCA

+ Frame 1 F * T V L S K F S * S D S C * L H

1010 1020 1030 1040 1050

+7 CF477528_Ptaeda ..................................................

CONSENSUS CCACCAGGCCATCACGACACAATAATTAAAAAAGCTCGCGGTGTCTGGGT

+ Frame 1 H Q A I T T Q * L K K L A V S G

1060 1070 1080 1090 1100

+7 CF477528_Ptaeda ..................................................

CONSENSUS AGTGGAATTTGCTGATCTGATTATGAAGAGCCCCAGATCCGATGACAGTA

+ Frame 1 * W N L L I * L * R A P D P M T V

1110 1120 1130 1140 1150

+7 CF477528_Ptaeda ..................................................

CONSENSUS TATGTGCTTCATATATTATGTACATCGGGATTCCGAAACGACATTTCGAG

+ Frame 1 Y V L H I L C T S G F R N D I S R

1160 1170 1180 1190 1200

+7 CF477528_Ptaeda ..................................................

CONSENSUS GAGATGAAAAATCGTGTTTTCTGAAACACTTCTGCATATTAATTTGGATG

+ Frame 1 R * K I V F S E T L L H I N L D

1210 1220 1230 1240

+7 CF477528_Ptaeda ...........................................

CONSENSUS TATCAGCTGTAATAATGATTCTTTAAAATATATTTATGTTTCA

+ Frame 1 V S A V I M I L * N I F M F -

1. ***Picea engelmannii × glauca CLE183***

10 20 30 40 50

-28 DR464239_PengelxPgla ..................................................

CONSENSUS GGAGAAGCGCTGTGTCGGATTGCATACCAAGACTTGCAGACAGACATTAA

+ Frame 1 G E A L C R I A Y Q D L Q T D I N

60 70 80 90 100

-28 DR464239_PengelxPgla ..................................................

CONSENSUS TAGCCATAACTGTGTGGAAACATATACAATTTTCTCTAATCCTCAGAGAT

+ Frame 1 S H N C V E T Y T I F S N P Q R

110 120 130 140 150

-28 DR464239_PengelxPgla ..................................................

-9 CO207045-1_PengelxPg ..............................................

CONSENSUS CTTATTAGTGTCAGTTTTCAGGACAGAAACAGAAACAGCCAGCCATGAGG

+ Frame 1 S Y * C Q F S G Q K Q K Q P A M R

160 170 180 190 200

-28 DR464239_PengelxPgla ..................................................

-9 CO207045-1_PengelxPg ..................................................

CONSENSUS GGATACTGTCTCATTGCTGCCAGGCTTAGTAGAATGATACTTTTCATCTT

+ Frame 1 G Y C L I A A R L S R M I L F I L

210 220 230 240 250

-28 DR464239_PengelxPgla ..................................................

-9 CO207045-1_PengelxPg ..................................................

CONSENSUS GCTCTTAGTAGTGTTATTATCTCTTAACAATGAAAGGGCTCTTGGTATGA

+ Frame 1 L L V V L L S L N N E R A L G M

260 270 280 290 300

-28 DR464239_PengelxPgla ..................................................

-9 CO207045-1_PengelxPg ..................................................

CONSENSUS GAAATATAGACAGGATAAAAGGAATTAGGCATGAGCTGTATACAGTAGGA

+ Frame 1 R N I D R I K G I R H E L Y T V G

310 320 330 340 350

-28 DR464239_PengelxPgla ......................................T...........

-9 CO207045-1_PengelxPg ......................................C...........

CONSENSUS GCTGCTGCTACTGAGGTTTCTCATGTGGATGCAAAAAGYAGAGGAAAAGA

+ Frame 1 A A A T E V S H V D A K S R G K E

360 370 380 390 400

-28 DR464239_PengelxPgla ..................................................

-9 CO207045-1_PengelxPg ..................................................

CONSENSUS ACCAGATTTCGATCTGAATTATGGGAATGTTAAGCGTAGAGTGCCGAATG

+ Frame 1 P D F D L N Y G N V K R R V P N

410 420 430 440 450

-28 DR464239_PengelxPgla ..................................................

-9 CO207045-1_PengelxPg ..................................................

CONSENSUS GATCCGACCCAATTCATAATAGAGCGGGGAAGTCAGGAGAGCCTCCAGCA

+ Frame 1 G S D P I H N R A G K S G E P P A

460 470 480 490 500

-28 DR464239_PengelxPgla ..................................................

-9 CO207045-1_PengelxPg ..................................................

CONSENSUS GTATAGAAGGAATGATGCTGTAGCGTTAAAATAGGAAGCAGAATTCAGAG

+ Frame 1 V * K E * C C S V K I G S R I Q S

510 520 530 540 550

-28 DR464239_PengelxPgla ..................................................

-9 CO207045-1_PengelxPg ..................................................

CONSENSUS TAGCGTGCTTTTACATGAAAATACAGCATTGTAATCCATAATGGGAACTA

+ Frame 1 S V L L H E N T A L * S I M G T

560 570 580 590 600

-28 DR464239_PengelxPgla ..................................................

-9 CO207045-1_PengelxPg ..................................................

CONSENSUS CCAATGCTAAGATGGTGGAGTGAATATTGTGATCTTTTCTCAGTTTGGCA

+ Frame 1 T N A K M V E * I L * S F L S L A

610 620 630 640 650

-28 DR464239_PengelxPgla ..................................................

-9 CO207045-1_PengelxPg ..................................................

CONSENSUS ACGATAAGGCGAGCTTATAATTACTTCTAGTGGTTTGGACATTGCCATGT

+ Frame 1 T I R R A Y N Y F * W F G H C H V

660 670 680 690 700

-28 DR464239_PengelxPgla ..................................................

-9 CO207045-1_PengelxPg ..................................................

CONSENSUS GCAGAGACAAAAATAGAAGAAGTAACAAATTTAGTTGCGACTGTAGAAGT

+ Frame 1 Q R Q K * K K * Q I * L R L * K

710 720 730 740 750

-28 DR464239_PengelxPgla ..................................................

-9 CO207045-1_PengelxPg ..................................................

CONSENSUS TCTTGTTTTTCATTTTACTATTCAATTAGTGTACAGAGAACAGATTGTTT

+ Frame 1 F L F F I L L F N * C T E N R L F

760 770 780 790 800

-28 DR464239_PengelxPgla .........................................*........

-9 CO207045-1_PengelxPg .........................................A........

CONSENSUS GCTTGATTGCTTGGTAGACATTTCCTACCTTACAAAAAAAA-TAAATAAA

+ Frame 1 A * L L G R H F L P Y K K - * I K

810 820 830 84

-28 DR464239_PengelxPgla ...................CC..................

-9 CO207045-1_PengelxPg ...................G*..................

CONSENSUS GTTGTATAATTATCGGCCC--AAAAAAAAAAAAAAAAAA

+ Frame 1 L Y N Y R P - K K K K K -

1. ***Picea sitchensis CLE184***

10 20 30 40 50

-10 CO223972_Psitchensis ..................................................

CONSENSUS GCTCCTCGTTTTGCTGCTAGTGTTTCTCTTCTCTTTCAGCCCGTGGAGCC

+ Frame 3 S S F C C * C F S S L S A R G A

60 70 80 90 100

-10 CO223972_Psitchensis ..................................................

CONSENSUS TGCGCGTACAATCAACGCATGTCGAAAAAACAAAGAGACTGCACGGAGAC

No predicted signal peptide. Sequence likely not full-length

+ Frame 3 C A Y N Q R M S K K Q R D C T E T

110 120 130 140 150

-10 CO223972_Psitchensis ..................................................

CONSENSUS GATGCATCGGCCTCATCATGTCCAGGTCACGAGATCGAATCCCCGGCGTC

+ Frame 3 M H R P H H V Q V T R S N P R R

160 170 180 190 200

-10 CO223972_Psitchensis ..................................................

CONSENSUS TTTCATCCGATCAGAACCAATTCAATGTTCCAGCTGCTGCGTCTCGTGGG

+ Frame 3 L S S D Q N Q F N V P A A A S R G

210 220 230 240 250

-10 CO223972_Psitchensis ..................................................

CONSENSUS CGTCATGCCCGCCGAAGCCCACCTTCGCCTTGGGCTAACTCTCAGCGTTA

+ Frame 3 R H A R R S P P S P W A N S Q R Y

260 270 280 290 300

-10 CO223972_Psitchensis ..................................................

CONSENSUS TAACGCCAGCGCCCATGAAGTTCCCAGCGGCCCAAATCCAATATCGAATT

+ Frame 3 N A S A H E V P S G P N P I S N

310 320 330 340 350

-10 CO223972_Psitchensis ..................................................

CONSENSUS GATAGATCATCAAGGTAATTAGGTTACTCATAAAACCCTGATTCATTTGC

+ Frame 3 * * I I K V I R L L I K P * F I C

360 370 380 390 400

-10 CO223972_Psitchensis ..................................................

CONSENSUS ACCAGCTGCAGTAACGTACTAGTCTTGCCAATATACCAGTTGCCATATGA

+ Frame 3 T S C S N V L V L P I Y Q L P Y E

410 420 430 440 450

-10 CO223972_Psitchensis .......................................A..........

-36 CO220733_Psitchensis G..........

CONSENSUS AATCAGGCAGCTCTATAATTGTATCGGGTGTACAGTACC-AATCTTCGGC

+ Frame 3 I R Q L Y N C I G C T V P N L R

460 470 480 490 500

-10 CO223972_Psitchensis ..................................................

-36 CO220733_Psitchensis ..................................................

CONSENSUS GGAGTATATAGTGATGTCTACTTATTTCTTTAGATATATATATGATGTGC

+ Frame 3 R S I * * C L L I S L D I Y M M C

510 520 530 540 550

-10 CO223972_Psitchensis ..................................................

-36 CO220733_Psitchensis ..................................................

CONSENSUS TGCTCAGTGCTGCTAAATTTGTTAAAAACTGTTGAGCTATTATATTGTTG

+ Frame 3 C S V L L N L L K T V E L L Y C C

560 570 580 590 600

-10 CO223972_Psitchensis ...............C.....................G........T...

-36 CO220733_Psitchensis ...............*.....................*........*...

CONSENSUS CTGTCCATTGTTTCC-GGTTACTAGTATTGGCCGCCG-ATTAGGCC-AAA

+ Frame 3 C P L F P G Y * Y W P P - * A -

610 620 630 640 650

-10 CO223972_Psitchensis .......................AA..A...A.A

-36 CO220733_Psitchensis .......................GG..T...G.G................

CONSENSUS TTATTTTTATTGAGGGAAAAAAA--AA-AAA-A-TATACAGAGATATTTA

+ Frame 3 I I F I E G K K - - - - I Q R Y L

660 670 680 690 700

-36 CO220733_Psitchensis ..................................................

CONSENSUS TTGAATGAACTGTAAGCCTTGTGCATTGTCATTTTTGAGGCCAATTGTGC

+ Frame 3 L N E L * A L C I V I F E A N C A

710 720 730 740 750

-36 CO220733_Psitchensis ..................................................

CONSENSUS ATGAATATATAAATTTCAGAAGATTGCAAGCCTAAAAAAAAAAAAAAAAA

+ Frame 3 * I Y K F Q K I A S L K K K K K

-36 CO220733_Psitchensis .

CONSENSUS A

+ Frame 3 -

1. ***Picea glauca CLE185***

10 20 30 40 50

-11 CO237812_Pglauca ..................................................

CONSENSUS TCAGCTAATTTTGTGGAAGCGATGAGTCAAGGCAATAAGAATCTCCAGGC

No predicted signal peptide. Sequence likely not full-length

+ Frame 1 S A N F V E A M S Q G N K N L Q A

60 70 80 90 100

-11 CO237812_Pglauca ..................................................

CONSENSUS CTCCCGTGAATCGGATGATACAGGGCAATATTGTTCTCATATATCATGCA

+ Frame 1 S R E S D D T G Q Y C S H I S C

110 120 130 140 150

-11 CO237812_Pglauca ..................................................

CONSENSUS GACGTGCAGGATATGAAGTGGATCCCAGATTTGGAGTTCAAAAGAGACTC

+ Frame 1 R R A G Y E V D P R F G V Q K R L

160 170 180 190 200

-11 CO237812_Pglauca ..................................................

CONSENSUS GTTCCTACGGGTCCTAATCCATTGCATAACTGAAGAAAATCAAATGTGTT

+ Frame 1 V P T G P N P L H N * R K S N V F

210 220 230 240 250

-11 CO237812_Pglauca ..................................................

CONSENSUS CAAAGAGTTGAAGGAGTCCACCATGCATTGAGTTCAGGAGCAGGTAGGCG

+ Frame 1 K E L K E S T M H * V Q E Q V G

260 270 280 290 300

-11 CO237812_Pglauca ..................................................

CONSENSUS GCCATCGCCTCTCAGTGATATATGCGTACATAAAAAAGATAAATGTTAAG

+ Frame 1 G H R L S V I Y A Y I K K I N V K

310 320 330 340 350

-11 CO237812_Pglauca ..................................................

CONSENSUS GATAATAATATATCTATCCGCAGGTTGGAAACAGAAGGTAAGTGCCATAC

+ Frame 1 D N N I S I R R L E T E G K C H T

360 370 380 390 400

-11 CO237812_Pglauca ..................................................

CONSENSUS GCCTGTTTTCTATGTAGTGATGACCATATCTTGTAATTGATTTCGATGCG

+ Frame 1 P V F Y V V M T I S C N * F R C

410 420 430 440 450

-11 CO237812_Pglauca ..................................................

CONSENSUS GTGCGTTGTATTGCTATTATCATAATAAATGAAATGGGATGGATTTACCA

+ Frame 1 G A L Y C Y Y H N K * N G M D L P

460

-11 CO237812_Pglauca .................

CONSENSUS AAAAAAAAAAAAAAAAA

+ Frame 1 K K K K K -

1. ***Picea glauca CLE186***

10 20 30 40 50

+68 EX332833_Pglauca ..................................................

CONSENSUS ATGCATTAAATTCATGTAAGTGGTATATGGTAAGGAGTTCAGGTACTTTG

+ Frame 1 M H * I H V S G I W * G V Q V L C

60 70 80 90 100

+68 EX332833_Pglauca ..................................................

+43 DV974393_Pglauca ............................

+67 GE481437_Pglauca ............................

+22 CO485442_Pglauca ............................

CONSENSUS TGAGTCGAAGATTCATCAGGGAGAAGATACGGGGGATTAATTTCAAAGCA

+ Frame 1 E S K I H Q G E D T G D * F Q S

110 120 130 140 150

+68 EX332833_Pglauca ..................................................

+43 DV974393_Pglauca ..................................................

+67 GE481437_Pglauca ..................................................

+22 CO485442_Pglauca ..................................................

CONSENSUS GAGGTTGAGAAGAAGCAGTGGTGAAGGATTTAATGGAAGAGCATGAAGCC

+ Frame 1 R G * E E A V V K D L M E E H E A

160 170 180 190 200

+68 EX332833_Pglauca ..................................................

+43 DV974393_Pglauca ..................................................

+67 GE481437_Pglauca ..................................................

+22 CO485442_Pglauca ..................................................

CONSENSUS CCGTGAGCTTTGAAGGCTGTTGATTGATCTTTTCCCTTTGCATTAACGAC

+ Frame 1 P * A L K A V D * S F P F A L T T

210 220 230 240 250

+68 EX332833_Pglauca ..................................................

+43 DV974393_Pglauca ..................................................

+67 GE481437_Pglauca ..................................................

+22 CO485442_Pglauca ..................................................

CONSENSUS ATTCATCAGCTTCACGTACCTGTGCAGTAAAGTTTTTATTTTTGGGTTCC

+ Frame 1 F I S F T Y L C S K V F I F G F

260 270 280 290 300

+68 EX332833_Pglauca ..................................................

+43 DV974393_Pglauca ..................................................

+67 GE481437_Pglauca ..................................................

+22 CO485442_Pglauca ..................................................

CONSENSUS TCCTTATCTTAAAGATCGGCTGCAATCCATCCTTGCTTTGCTTCATCTCA

+ Frame 1 L L I L K I G C N P S L L C F I S

310 320 330 340 350

+68 EX332833_Pglauca ..................................................

+43 DV974393_Pglauca ..................................................

+67 GE481437_Pglauca ..................................................

+22 CO485442_Pglauca ..................................................

CONSENSUS CCACCGTCCATCTCTCTGTCTCTGCTCTCGTGAAACTGTTTAATTTCTAG

+ Frame 1 P P S I S L S L L S * N C L I S R

360 370 380 390 400

+68 EX332833_Pglauca ..................................................

+43 DV974393_Pglauca ..................................................

+67 GE481437_Pglauca ..................................................

+22 CO485442_Pglauca ..................................................

CONSENSUS ACTGTTCAGCTTTCTATATAATTTACTGCACTCGCCTCTGAGATTTTGGC

+ Frame 1 L F S F L Y N L L H S P L R F W

410 420 430 440 450

+68 EX332833_Pglauca ..................................................

+43 DV974393_Pglauca ..................................................

+67 GE481437_Pglauca ..................................................

+22 CO485442_Pglauca ..................................................

CONSENSUS TGTCCGGGATTTCTGTGCTGGAATTCGTTTTATATTCAGATCGATTCCTT

+ Frame 1 L S G I S V L E F V L Y S D R F L

460 470 480 490 500

+68 EX332833_Pglauca ..................................................

+43 DV974393_Pglauca ..................................................

+67 GE481437_Pglauca ..................................................

+22 CO485442_Pglauca ..................................................

-21 CO484210_Pglauca ......

CONSENSUS CTTTTGTTTGGTTTTCTGATATAGCACAGTACTGTATATGATATATTTTT

+ Frame 1 L L F G F L I * H S T V Y D I F F

510 520 530 540 550

+68 EX332833_Pglauca ..................................................

+43 DV974393_Pglauca ..................................................

+67 GE481437_Pglauca ..................................................

+22 CO485442_Pglauca ..................................................

-21 CO484210_Pglauca ...............GGA................................

CONSENSUS TAGACCTATTATCGG-TGAAATTGCTGGGACGATGATTGAGAGGAGAAGG

+ Frame 1 R P I I G E I A G T M I E R R R

560 570 580 590 600

+68 EX332833_Pglauca ................................................C.

+43 DV974393_Pglauca ..................................................

+67 GE481437_Pglauca ..................................................

+22 CO485442_Pglauca ..................................................

-21 CO484210_Pglauca ..................................................

-12 CO239410_Pglauca .................................................

CONSENSUS CCTGAGAAATTGAATAGGATGATGAATCTTGCTGCGGTGGTTAGCGTATT

+ Frame 1 P E K L N R M M N L A A V V S V L

610 620 630 640 650

+68 EX332833_Pglauca ..................................................

+43 DV974393_Pglauca ..................................................

+67 GE481437_Pglauca ..................................................

+22 CO485442_Pglauca ..................................................

-21 CO484210_Pglauca ..................................................

-12 CO239410_Pglauca ..................................................

CONSENSUS GGTAGTAATGATCCTGATTATACTCTCCAGTTTAATATGTTTTGCATCTG

+ Frame 1 V V M I L I I L S S L I C F A S

660 670 680 690 700

+68 EX332833_Pglauca ..................................................

+43 DV974393_Pglauca ..................................................

+67 GE481437_Pglauca ..................................................

+22 CO485442_Pglauca ..................................................

-21 CO484210_Pglauca ..................................................

-12 CO239410_Pglauca ..................................................

CONSENSUS CAGCAAGGCAGTCCGCGTTTTTCCATGCAGAGATGAAGGATAAAGATCAT

+ Frame **1** A A R Q S A F F H A E M K D K D H

710 720 730 740 750

+68 EX332833_Pglauca ..................................................

+43 DV974393_Pglauca ..................................................

+67 GE481437_Pglauca ..................................................

+22 CO485442_Pglauca ..................................................

-21 CO484210_Pglauca ..................................................

-12 CO239410_Pglauca ..................................................

CONSENSUS AAAGCTGCCTCCGGTTTGTTTAAACCCTCTGGCAAGGATTGCCATTCAGG

+ Frame 1 K A A S G L F K P S G K D C H S G

760 770 780 790 800

+68 EX332833_Pglauca ..................................................

+43 DV974393_Pglauca .....................................N............

+67 GE481437_Pglauca ..................................................

+22 CO485442_Pglauca ..................................................

-21 CO484210_Pglauca ..................................................

-12 CO239410_Pglauca ..................................................

+73 GR953968_Pglauca .................

CONSENSUS GAAATCTCTCAGCCACTGCAGCCCTATTTCGAAGCAGATGGGCAATAGCA

+ Frame 1 K S L S H C S P I S K Q M G N S

810 820 830 840 850

+68 EX332833_Pglauca ...........................................

+43 DV974393_Pglauca ..................................................

+67 GE481437_Pglauca ..................................................

+22 CO485442_Pglauca ..................................................

-21 CO484210_Pglauca ..................................................

-12 CO239410_Pglauca ..................................................

+73 GR953968_Pglauca ..................................................

CONSENSUS ATATGACTGGAGCAGATAAACGCGTAGTGCCCACTGGCCCAAATCCCTTG

+ Frame 1 N M T G A D K R V V P T G P N P L

860 870 880 890 900

+43 DV974393_Pglauca ........................NNNN......................

+67 GE481437_Pglauca ..................................................

+22 CO485442_Pglauca ..................................................

-21 CO484210_Pglauca ..................................................

-12 CO239410_Pglauca .............C....................................

+73 GR953968_Pglauca .............C....................................

-33 EX333178_Pglauca .....................

CONSENSUS CACAACAGGTGAATCATATCAGTCCAAGGATTGTATGATATTAATCATCA

+ Frame 1 H N R * I I S V Q G L Y D I N H H

910 920 930 940 950

+43 DV974393_Pglauca ....................

+67 GE481437_Pglauca ..................................................

+22 CO485442_Pglauca ...........NC................................*....

-21 CO484210_Pglauca ..................................................

-12 CO239410_Pglauca ..................................................

+73 GR953968_Pglauca ......................................A...........

-33 EX333178_Pglauca ......................................A...........

CONSENSUS TGGAGGCTGATCAACCTAGGGTTGGGATTTCAGTGCAGGAAAAACCAACT

+ Frame 1 G G * S T * G W D F S A G K T N

960 970 980 990 1000

+67 GE481437_Pglauca ..................................................

+22 CO485442_Pglauca ..................................................

-21 CO484210_Pglauca ..................................................

-12 CO239410_Pglauca ......................----........................

+73 GR953968_Pglauca ....................AT....TA............A.........

-33 EX333178_Pglauca ....................AT....TA............A.........

CONSENSUS ATATCTATCTATCTATCTAT--CTATATCTATTACTATTACTATATCTTA

+ Frame 1 Y I Y L S I Y L Y L L L L L Y L

1010 1020 1030 1040 1050

+67 GE481437_Pglauca ...........

+22 CO485442_Pglauca ...................

-21 CO484210_Pglauca ..................................................

-12 CO239410_Pglauca ..........................................T.......

+73 GR953968_Pglauca ...............................................T..

-33 EX333178_Pglauca ...............................................T..

CONSENSUS TCTATGCAAAAACTGAGCTGTGTTAATGTTTTAGGCTGCCTGAGAAACTC

+ Frame 1 I Y A K T E L C * C F R L P E K L

1060 1070 1080 1090 1100

-21 CO484210_Pglauca ..................................................

-12 CO239410_Pglauca ..................................................

+73 GR953968_Pglauca ..................................................

-33 EX333178_Pglauca ..................................................

CONSENSUS TGTTGAAGATGTTCATGAGAGCCATGAACAGGACCAGCAAAAGGAGTATT

+ Frame 1 C * R C S * E P * T G P A K G V L

1110 1120 1130 1140 1150

-21 CO484210_Pglauca ..................................................

-12 CO239410_Pglauca ..................................................

+73 GR953968_Pglauca ..................................................

-33 EX333178_Pglauca ..................................................

CONSENSUS GTAACATAGAGACGATGATGCTATTTGATCATAGCTGCAATCTTCTGGTG

+ Frame 1 * H R D D D A I * S * L Q S S G

1160 1170 1180 1190 1200

-21 CO484210_Pglauca ..................................................

-12 CO239410_Pglauca ..................................................

+73 GR953968_Pglauca ..................................................

-33 EX333178_Pglauca ..................................................

CONSENSUS TATTAAGTTAAATTGTGGTAGGTAGAGTATTGGCTCAACGGTTTTCCATG

+ Frame 1 V L S * I V V G R V L A Q R F S M

1210 1220 1230 1240 1250

-21 CO484210_Pglauca ..................................................

-12 CO239410_Pglauca .................G................................

+73 GR953968_Pglauca ..................................................

-33 EX333178_Pglauca ..................................................

CONSENSUS TTGGACCGAATTTTGTCTCAGTATATATTATTTTATACATATGTTGATAT

+ Frame 1 L D R I L S Q Y I L F Y T Y V D I

1260 1270 1280 1290 1300

-21 CO484210_Pglauca ..................................................

-12 CO239410_Pglauca ..................................................

+73 GR953968_Pglauca ..................................................

-33 EX333178_Pglauca ..................................................

CONSENSUS ATGCCACTTTTAGCTTCTGGAGCATTGGTTCCATGTCCCAGAAGCTAAAA

+ Frame 1 C H F * L L E H W F H V P E A K

1310 1320 1330 1340 1350

-21 CO484210_Pglauca ........................A.........................

-12 CO239410_Pglauca .......A.........T................................

+73 GR953968_Pglauca ..................................................

-33 EX333178_Pglauca ..................................................

CONSENSUS GTGGGGTGAAATCATTCCATGTTCGCAGCGAACAGCAGCAAATTTGTTCT

+ Frame 1 S G V K S F H V R S E Q Q Q I C S

1360 1370 1380 1390 1400

-21 CO484210_Pglauca ........................

-12 CO239410_Pglauca ..................................................

+73 GR953968_Pglauca ..................................................

-33 EX333178_Pglauca ..................................................

CONSENSUS TGTCCAATGGCTAATTGGAATCTATTTTATACATTTGGTATCTATTTACT

+ Frame 1 C P M A N W N L F Y T F G I Y L L

1410 1420 1430 1440 1450

-12 CO239410_Pglauca .A.AA.AA.AAAA.AAA.

+73 GR953968_Pglauca ..................................................

-33 EX333178_Pglauca ..................................................

CONSENSUS AGATCATGAGTTCAGGCATGCCGATCTGCAGAAGATCTTCAAATGTATTG

+ Frame 1 D H E F R H A D L Q K I F K C I

1460 1470 1480 1490 1500

+73 GR953968_Pglauca ..................................................

-33 EX333178_Pglauca ..................................................

CONSENSUS ATGATTATACCTATTGTCAAGTTTTTGCAGTTGCTTGTATCTCTCAGCTT

+ Frame 1 D D Y T Y C Q V F A V A C I S Q L

1510 1520 1530 1540 1550

+73 GR953968_Pglauca ..................................................

-33 EX333178_Pglauca ..................................................

CONSENSUS TTATAACACAAAAGAGAAGAAAAAGCATGAAGATAACTTGCGAAAGTCTG

+ Frame 1 L * H K R E E K A * R * L A K V *

1560 1570 1580 1590 1600

+73 GR953968_Pglauca ..................................................

-33 EX333178_Pglauca ..................................................

CONSENSUS AATCGAGCTATTATAAGCTGTTCACCGAGAATTTGGTATTTTACTATCTG

+ Frame 1 I E L L * A V H R E F G I L L S

1610 1620 1630 1640 1650

+73 GR953968_Pglauca ................................TAT.....T.........

-33 EX333178_Pglauca ................................ATA.....A.........

CONSENSUS TTTTTATGATTTGTATCTGAATGTACAGGATT---ACACA-TAGATTAAA

+ Frame 1 V F M I C I * M Y R I - T - * I K

+73 GR953968_Pglauca ...

-33 EX333178_Pglauca ...

CONSENSUS AAA

+ Frame 1 K

1. ***Picea glauca CLE187***

10 20 30 40 50

-13 CO256846_Pglauca ..................................................

CONSENSUS CTTTCGCCGGCAGAGATGGATAATAAGTTCGAAGGCGACAAACGTCTAAT

+ Frame 1 L S P A E M D N K F E G D K R L I

No predicted signal peptide. Sequence likely not full-length

60 70 80 90 100

-13 CO256846_Pglauca ..................................................

CONSENSUS TCCAACCGGCCCGAATCCGTTGCATAACAGATAGTGGAGTTCGAAATAGC

+ Frame 1 P T G P N P L H N R * W S S K *

110 120 130 140 150

-13 CO256846_Pglauca ..................................................

CONSENSUS TTAAACGCAGGTTGGTTAATGTCAATGCAGGTGGAAATTTTCAAGCTATC

+ Frame 1 L K R R L V N V N A G G N F Q A I

160 170 180 190 200

-13 CO256846_Pglauca ..................................................

CONSENSUS CTCCAAATTTTGCTCATTAGGTTTCGAAGATAAGGCTGGTGCAAGATAAT

+ Frame 1 L Q I L L I R F R R * G W C K I M

210 220 230 240 250

-13 CO256846_Pglauca ..................................................

CONSENSUS GTCGATTGAGAATCGACAAAGCTACAGGAACTGGTGTTACAAGGAAATGG

+ Frame 1 S I E N R Q S Y R N W C Y K E M

260 270 280 290 300

-13 CO256846_Pglauca ..................................................

CONSENSUS TGTTATGCTTGTTTGCTCGGCGTTCCTTTCGTTTTTTCGGCTTTCCCTTC

+ Frame 1 V L C L F A R R S F R F F G F P F

310 320 330 340 350

-13 CO256846_Pglauca ..................................................

CONSENSUS TTCTTTTATAGGATATGAGATATGTGTGAAACAATAGTTAGAACGGCTTT

+ Frame 1 F F Y R I * D M C E T I V R T A F

360 370 380 390 400

-13 CO256846_Pglauca ..................................................

CONSENSUS CCCTTCTTCTTTTATAGGATATGAGATATGTGTGAAACAATAGTTAGAAC

+ Frame 1 P S S F I G Y E I C V K Q * L E

410 420 430 440 450

-13 CO256846_Pglauca ..................................................

CONSENSUS CCATTTATCTTGTAATTTCTTGGAATACTTCTAAATGTATAAAATCTCCT

+ Frame 1 P I Y L V I S W N T S K C I K S P

460 470 480 490 500

-13 CO256846_Pglauca ..................................................

CONSENSUS CTCTGCATCATGTACAAGCGATTTTTATTCAGCTCCGGCATTATAGTCAG

+ Frame 1 L C I M Y K R F L F S S G I I V R

510 520 530 540 550

-13 CO256846_Pglauca ..................................................

CONSENSUS AATTCAAACTCAAATGTAAAATAAAAAAAAAAAAAAAAAAAAAAAAAAAA

+ Frame 1 I Q T Q M * N K K K K K K K K K

560

-13 CO256846_Pglauca .................

CONSENSUS GAAAAAAAAAAAAAAAA

+ Frame 1 R K K K K K

1. ***Pinus taeda CLE188***

10 20 30 40 50

+15 CO366019_Ptaeda ..................................................

CONSENSUS TAGGAATTCAGCTCAGGAGCCTCCGAATATATTAATCCTTTCGCGCTAAG

+ Frame 3 G I Q L R S L R I Y * S F R A K

60 70 80 90 100

+15 CO366019_Ptaeda ..................................................

CONSENSUS CAGTTTGGAGTTTAAAGATTTTTGAACTTTGATTGAAATCGCCTGTTACA

+ Frame 3 Q F G V * R F L N F D * N R L L Q

110 120 130 140 150

+15 CO366019_Ptaeda ..................................................

+14 CO365940_Ptaeda .................................................

CONSENSUS GGGCCAGGTGTGACAAAGAGTTAATGGAAATTGAGCTCACTGGTTCGTTT

+ Frame 3 G Q V * Q R V N G N * A H W F V

160 170 180 190 200

+15 CO366019_Ptaeda ..................................................

+14 CO365940_Ptaeda ..................................................

CONSENSUS GGGGTATAAGAAGCAAAAAGTGCAGCTGAATGGCAGATGCTTTAGTGGAT

+ Frame 3 W G I R S K K C S * M A D A L V D

210 220 230 240 250

+15 CO366019_Ptaeda ..................................................

+14 CO365940_Ptaeda ..................................................

CONSENSUS CCAAGGTGCCATTGCACGCTGAGACAATGGAAGAGCACAAGCAAAGATGA

+ Frame 3 P R C H C T L R Q W K S T S K D D

260 270 280 290 300

+15 CO366019_Ptaeda ..................................................

+14 CO365940_Ptaeda ..................................................

CONSENSUS TAACAGGTCAAGTAGGCGTATAAGTTTTTCAAGATTATTTAATCTGTTCC

+ Frame 3 N R S S R R I S F S R L F N L F

310 320 330 340 350

+15 CO366019_Ptaeda ..................................................

+14 CO365940_Ptaeda ..................................................

CONSENSUS TCGTTTTGCTGCTGGTGTTTCCTCTTCTCTCTCAGCCCGTTGAGCCTGCG

+ Frame 3 L V L L L V F P L L S Q P V E P A

360 370 380 390 400

+15 CO366019_Ptaeda ..................................................

+14 CO365940_Ptaeda ..................................................

CONSENSUS CGTACAATCAACGCATGCCAGAAAAAGCAAAGAAACTGCACGCAGGAGAT

+ Frame 3 R T I N A C Q K K Q R N C T Q E M

410 420 430 440 450

+15 CO366019_Ptaeda ..................................................

+14 CO365940_Ptaeda ..................................................

CONSENSUS GCATCGGCCTCAAGATGTCAAGGTCACGAGTTCGAATCCCCGGCGTCTTT

+ Frame 3 H R P Q D V K V T S S N P R R L

460 470 480 490 500

+15 CO366019_Ptaeda ..................................................

+14 CO365940_Ptaeda ..................................................

CONSENSUS CATCCGATCAGAACCAATTCAATGTTCCAGCTGCTCCTTCACGTGGCCGT

+ Frame 3 S S D Q N Q F N V P A A P S R G R

510 520 530 540 550

+15 CO366019_Ptaeda ..................................................

+14 CO365940_Ptaeda ..................................................

CONSENSUS CATGGCCGCCGAAGCCCTCCTTCACCTTGGGCTAATTCTAGGCGTTATAA

+ Frame 3 H G R R S P P S P W A N S R R Y N

560 570 580 590 600

+15 CO366019_Ptaeda ..................................................

+14 CO365940_Ptaeda ..................................................

CONSENSUS CGCTAGCGATCATGAAGTTCCCAGCGGTCCAAATCCAATATCAAATTGAT

+ Frame 3 A S D H E V P S G P N P I S N *

610 620 630 640 650

+15 CO366019_Ptaeda ..................................................

+14 CO365940_Ptaeda ..................................................

CONSENSUS AGATCCTCAAGGTAATTAACATAGTCATAAAACCCTGATTCATTTGCACC

+ Frame 3 * I L K V I N I V I K P * F I C T

660 670 680 690 700

+15 CO366019_Ptaeda ..................................................

+14 CO365940_Ptaeda ..................................................

CONSENSUS AACTGCAGTAACTTGCTAGTCTTTCCAGTATACCAGTTGCTTCATATGAG

+ Frame 3 N C S N L L V F P V Y Q L L H M R

710 720 730 740 750

+15 CO366019_Ptaeda ..................................................

+14 CO365940_Ptaeda ..................................................

CONSENSUS ATCAGGAAGCTCTTTAATTGTATCGGGTGTACAGTACCAAAGCTTCGGCG

+ Frame 3 S G S S L I V S G V Q Y Q S F G

760 770 780 790 800

+15 CO366019_Ptaeda ..................................................

+14 CO365940_Ptaeda ..................................................

CONSENSUS GAGTATATAGTGGTGTCTACTTATTTCTTTAGATATTTATATATCATGTG

+ Frame 3 G V Y S G V Y L F L * I F I Y H V

810 820 830 840 850

+15 CO366019_Ptaeda ..................................................

+14 CO365940_Ptaeda ..................................................

CONSENSUS CTGCTCAGTGCTGGTAAATTTGTTAAAGACTGTTAAAGCTATTAGAATGT

+ Frame 3 L L S A G K F V K D C * S Y * N V

860 870 880 890 900

+15 CO366019_Ptaeda .......

+14 CO365940_Ptaeda ..................................................

CONSENSUS TGCCATTGTTTCCGGTGACTAGTATTGGTCGCCGATTAGGCCAAATTATT

+ Frame 3 A I V S G D * Y W S P I R P N Y

910 920 930 940

+14 CO365940_Ptaeda ............................................

CONSENSUS TTTATTGAGGAGAAAAAAGGGAATAAAGAGTATACAGAGATATT

+ Frame 3 F Y * G E K R E * R V Y R D I

1. ***Pinus taeda CLE189***

10 20 30 40 50

-40 DR744109_Ptaeda ..................................................

CONSENSUS CTTCGAGAAATTCAGGAAACGGCGTGTCAGAATTAGATCCAATGATAAGG

+ Frame 1 L R E I Q E T A C Q N * I Q * * G

60 70 80 90 100

-40 DR744109_Ptaeda ..................................................

CONSENSUS CAATGAAGAAATAATTATGACAGTTTGAATATAATTTCATGGTTTTGTTT

+ Frame 1 N E E I I M T V * I * F H G F V

110 120 130 140 150

-40 DR744109_Ptaeda ..................................................

CONSENSUS GTTTTTGTAGAGGGAAGCAGGGGATTTTGAACTTGATTTTCTTTGGCTTA

+ Frame 1 C F C R G K Q G I L N L I F F G L

160 170 180 190 200

-40 DR744109_Ptaeda ..................................................

CONSENSUS CATAATACTTTATGGGATGGCCAATTGAGCTTTCATCGTTTTGCATAAGG

+ Frame 1 H N T L W D G Q L S F H R F A * G

210 220 230 240 250

-40 DR744109_Ptaeda ..................................................

CONSENSUS TTGGTTCGAAATAAGAGATTTTAAATTTGAATTTCTTGGTTTACATAATA

+ Frame 1 W F E I R D F K F E F L G L H N

260 270 280 290 300

-40 DR744109_Ptaeda ..................................................

CONSENSUS CTTGATAGCATGGCGATCACCAACTGACCTGCCCGGATAGACTTGAGAAA

+ Frame 1 T * * H G D H Q L T C P D R L E K

310 320 330 340 350

-40 DR744109_Ptaeda ..................................................

CONSENSUS CTCATAACATTAACCGTGGGGTATTGGTATTCTTTGCTTCAATAAATTGT

+ Frame 1 L I T L T V G Y W Y S L L Q * I V

360 370 380 390 400

-40 DR744109_Ptaeda ..................................................

CONSENSUS TGCATTCCAGGCTTTGAGACTTGGATGGATTTGGGTTTCGCTAGGCATCT

+ Frame 1 A F Q A L R L G W I W V S L G I

410 420 430 440 450

-40 DR744109_Ptaeda ..................................................

CONSENSUS TACAGGGGGCAGTCTGTATGGCGGATGGTTTTGTTAGAAGGCTTAAAAGG

+ Frame 1 L Q G A V C M A D G F V R R L K R

460 470 480 490 500

-40 DR744109_Ptaeda ..................................................

CONSENSUS GCAGATAAAAAGTTGCTGCTGATAATTTTTCTGTTGTTGGGTCTCTTCTT

+ Frame 1 A D K K L L L I I F L L L G L F F

510 520 530 540 550

-40 DR744109_Ptaeda ..................................................

CONSENSUS TCAGCGAGTAGATTCAACACCATGCCCAGGAAAAAAGTTGCAGGATTTTG

+ Frame 1 Q R V D S T P C P G K K L Q D F

560 570 580 590 600

-40 DR744109_Ptaeda ..................................................

CONSENSUS GTCATGGAGAATTAGGGGTTACATTGAAGCATTCTCCATGTCGAAGGATC

+ Frame 1 G H G E L G V T L K H S P C R R I

610 620 630 640 650

-40 DR744109_Ptaeda ..................................................

+86 DR089394_Ptaeda .............................

CONSENSUS TTGGCTTCCAGGGAATTCTTTGTTCCCAAGCACTCTAAGGGCTCCAGAAC

+ Frame 1 L A S R E F F V P K H S K G S R T

660 670 680 690 700

-40 DR744109_Ptaeda ..................................................

+86 DR089394_Ptaeda ..................................................

CONSENSUS ACATTATGAGGAAAGCGATCATGAAGTTCCCAGCGGTCCAAATCCAATTA

+ Frame 1 H Y E E S D H E V P S G P N P I

710 720 730 740 750

-40 DR744109_Ptaeda ....................*.............................

+86 DR089394_Ptaeda ....................C.............................

CONSENSUS GTAATAGGTAAGGATTTTAG-ATGTAGATTTTCACAGTTTATATAAGACT

+ Frame 1 S N R * G F * - V D F H S L Y K T

760 770 780 790 800

-40 DR744109_Ptaeda ..................................................

+86 DR089394_Ptaeda ..................................................

CONSENSUS GTGCTATGTAGTTGTGGTGTCCAATGTTTTTGGTGAACCCTTCATTTATC

+ Frame 1 V L C S C G V Q C F W * T L H L S

810 820 830 840 850

-40 DR744109_Ptaeda ..................................................

+86 DR089394_Ptaeda ..................................................

CONSENSUS TTCGAAGACAGAAAGAAATGAATGAATAGAGAGTAATCAGCCAAGAAATC

+ Frame 1 S K T E R N E * I E S N Q P R N

860 870 880 890 900

-40 DR744109_Ptaeda ............................

+86 DR089394_Ptaeda ..................................................

CONSENSUS TGACAAGTAATTGGTAAGGATTTAATGCAGATGGAGGGTGATGATCTTGA

+ Frame 1 L T S N W * G F N A D G G * * S *

910 920 930 940 950

+86 DR089394_Ptaeda ..................................................

CONSENSUS GTTCATGGTCAGTCAGGCATTGATTATTAAATTTCCAATTTCAGATTCTG

+ Frame 1 V H G Q S G I D Y * I S N F R F *

960 970 980 990 1000

+86 DR089394_Ptaeda ..................................................

CONSENSUS ATTCAGATGGGTTCTTGTTATGGTTAATTTTAGATTAATTGCAGAGTTGG

+ Frame 1 F R W V L V M V N F R L I A E L

1010 1020 1030 1040 1050

+86 DR089394_Ptaeda ..................................................

CONSENSUS GTGTTAATAACTTTTTCTATGGCAATGCCACCATCGTCGAATATGTCAGA

+ Frame 1 G V N N F F Y G N A T I V E Y V R

1060 1070 1080 1090 1100

+86 DR089394_Ptaeda ..................................................

CONSENSUS TCGGATCAGCTTTCTCTGGTGTGTTTTGATAAAGAGCTCAGAATATCGGG

+ Frame 1 S D Q L S L V C F D K E L R I S G

1110 1120 1130 1140 1150

+86 DR089394_Ptaeda ..................................................

CONSENSUS GTTCGAAGCTTCATCGAATACTGTAAACTAATATTTCTCAGTGTCCATGC

+ Frame 1 F E A S S N T V N * Y F S V S M

1160 1170 1180 1190 1200

+86 DR089394_Ptaeda ..................................................

CONSENSUS CAGAGCTCTTTCGGCACTTGCAATACGGTATAATATCAGATAAAGTTATA

+ Frame 1 P E L F R H L Q Y G I I S D K V I

1210 1220 1230 1240 1250

+86 DR089394_Ptaeda ..................................................

CONSENSUS GGGAAATATTTTAAAGAGATAGCCAGTACTTCTCTTTAAATTCACCATTG

+ Frame 1 G K Y F K E I A S T S L * I H H C

1260 1270 1280 1290 1300

+86 DR089394_Ptaeda ..................................................

CONSENSUS CCAAAAGATCACACTTTTCTTTTTATCAAGCATCTGTATTCTTATGTGGT

+ Frame 1 Q K I T L F F L S S I C I L M W

1310 1320 1330 1340 1350

+86 DR089394_Ptaeda ..................................................

CONSENSUS CTGCTGTAAGAAAAAAGAAAAAGTAGTCAGGGAGGATTGAGAAAAGGGTA

+ Frame 1 S A V R K K K K * S G R I E K R V

1360 1370 1380 1390 1400

+86 DR089394_Ptaeda ..................................................

CONSENSUS TAAGAATATAGAACTGCGCTTGCTGCAGCTATAAGAACTTTTGATATGTA

+ Frame 1 * E Y R T A L A A A I R T F D M Y

1410 1420 1430 14

+86 DR089394_Ptaeda ......................................

CONSENSUS TTTTTTCTTTATTATGAGTGGCCAAATTTAGATCTGAT

+ Frame 1 F F F I M S G Q I * I *

1. ***Picea glauca CLE190***

10 20 30 40 50

+44 DV993702_Pglauca ..................................................

+65 GE474919_Pglauca ..................................................

+16 CO475409_Pglauca ..................................................

CONSENSUS ATTCAGAGGTCTGCTCGGAAGGCAGAAGGTTCAGATGGCCAAAAACCATT

+ Frame 2 F R G L L G R Q K V Q M A K N H

60 70 80 90 100

+44 DV993702_Pglauca ..................................................

+65 GE474919_Pglauca ..................................................

+16 CO475409_Pglauca ..................................................

CONSENSUS TTATGTTTCCAAAGCTCAGTGCTAGTTATGATGTAGTCATGATCTTCTTG

+ Frame 2 F M F P K L S A S Y D V V M I F L

110 120 130 140 150

+44 DV993702_Pglauca ..................................................

+65 GE474919_Pglauca ..................................................

+16 CO475409_Pglauca ..................................................

CONSENSUS CTTGTAGTTTCTTCTCAGTTGATCTCTGCAGCTCTGGGCATTAGAAACTT

+ Frame 2 L V V S S Q L I S A A L G I R N F

160 170 180 190 200

+44 DV993702_Pglauca ..................................................

+65 GE474919_Pglauca ..................................................

+16 CO475409_Pglauca ..................................................

CONSENSUS CAATTCTTCAGAGAATATGCAGAAACAGCGACTGCTGGATGGCTTATCAG

+ Frame 2 N S S E N M Q K Q R L L D G L S

210 220 230 240 250

+44 DV993702_Pglauca ..................................................

+65 GE474919_Pglauca ..................................................

+16 CO475409_Pglauca ..................................................

CONSENSUS CAGCTACAGTCATGTACTCAGCTAATAAAAATGGGCAGCCTGATGGATTC

+ Frame 2 A A T V M Y S A N K N G Q P D G F

260 270 280 290 300

+44 DV993702_Pglauca ..................................................

+65 GE474919_Pglauca ..................................................

+16 CO475409_Pglauca ..................................................

CONSENSUS AAAGCTGATGTTACTGCAACTAATCTGGATCCAAATTTCACCAGCAAGCG

+ Frame 2 K A D V T A T N L D P N F T S K R

310 320 330 340 350

+44 DV993702_Pglauca ..................................................

+65 GE474919_Pglauca ..................................................

+16 CO475409_Pglauca ..................................................

-87 CO475124.2_Pglauca ......................................

CONSENSUS CATGGTTCCTAATGGGTCAGACCCTCTTCATAATCGGTGATCTTACATAT

+ Frame 2 M V P N G S D P L H N R * S Y I

360 370 380 390 400

+44 DV993702_Pglauca ..................................................

+65 GE474919_Pglauca ..................................................

+16 CO475409_Pglauca ..................................................

-87 CO475124.2_Pglauca ..................................................

CONSENSUS ATGTACAGATCGATTCCAAGAGTACTAGTTTAGGGAATATCAATGGCTCC

+ Frame 2 Y V Q I D S K S T S L G N I N G S

410 420 430 440 450

+44 DV993702_Pglauca ..................................................

+65 GE474919_Pglauca ..................................................

+16 CO475409_Pglauca ..................................................

-87 CO475124.2_Pglauca ..................................................

CONSENSUS TACCCTCTTAATAATCGGTGATCTTACATATATGTACAGATCGATTCCAA

+ Frame 2 Y P L N N R * S Y I Y V Q I D S K

460 470 480 490 500

+44 DV993702_Pglauca ..................................................

+65 GE474919_Pglauca ..................................................

+16 CO475409_Pglauca ..................................................

-87 CO475124.2_Pglauca ..................................................

CONSENSUS GAGTACTAGTTTAGGGAAAGGGAATATCAATGTAAGCAGAGGATGAAGGA

+ Frame 2 S T S L G K G N I N V S R G * R

510 520 530 540 550

+44 DV993702_Pglauca ..................................................

+65 GE474919_Pglauca ..................................................

+16 CO475409_Pglauca ..................................................

-87 CO475124.2_Pglauca ..................................................

CONSENSUS GAAGATTAGAATTCTGTCACTGAAGCTATTCCAAGACATCATAATATACA

+ Frame 2 R R L E F C H * S Y S K T S * Y T

560 570 580 590 600

+44 DV993702_Pglauca ..................................................

+65 GE474919_Pglauca ..................................................

+16 CO475409_Pglauca ..................................................

-87 CO475124.2_Pglauca ..................................................

CONSENSUS GTACTAGTCCAGTGAATATCAATACAAGCAGAAGAGGAAAAATAAGATTA

+ Frame 2 V L V Q * I S I Q A E E E K * D Y

610 620 630 640 650

+44 DV993702_Pglauca ..................................................

+65 GE474919_Pglauca ..................................................

+16 CO475409_Pglauca ..................................................

-87 CO475124.2_Pglauca ..................................................

CONSENSUS TAAAGTATTAATCCAGCAGCAAATATCAATGTAAGCAGAAGAGGAGGAAG

+ Frame 2 K V L I Q Q Q I S M * A E E E E

660 670 680 690 700

+44 DV993702_Pglauca ..................................................

+65 GE474919_Pglauca ..................................................

+16 CO475409_Pglauca ..................................................

-87 CO475124.2_Pglauca ..................................................

CONSENSUS AAGAAAATAATTATTCACACAGGCCACATAGAAGAAGAGGAGGAGGAGGA

+ Frame 2 E E N N Y S H R P H R R R G G G G

710 720 730 740 750

+44 DV993702_Pglauca ..................................................

+65 GE474919_Pglauca ..................................................

+16 CO475409_Pglauca ..................................................

-87 CO475124.2_Pglauca ..................................................

CONSENSUS GATCGTCCAGCAAGAAAACATGTAGATATGGATATGAGTAAAAGTGATGT

+ Frame 2 D R P A R K H V D M D M S K S D V

760 770 780 790 800

+44 DV993702_Pglauca ...............N.NNNNNN.......................N.NN

+65 GE474919_Pglauca ..................................................

+16 CO475409_Pglauca ..................................................

-87 CO475124.2_Pglauca ..................................................

CONSENSUS GCTCAATGCTAGAATTAAGAAACTCATACAGAGTTCTTTATGTTTTGGGT

+ Frame 2 L N A R I K K L I Q S S L C F G

810 820 830 840 850

+44 DV993702_Pglauca NNNNN.N.......NNNNNNNNNN.........NNNNN............

+65 GE474919_Pglauca ..................................................

+16 CO475409_Pglauca ..................................................

-87 CO475124.2_Pglauca ..................................................

CONSENSUS TGGTTTTTCCAATCAGAAGAGATACTGAGCTTCAGATTTCACCTATTTCC

+ Frame 2 L V F P I R R D T E L Q I S P I S

860 870 880 890 900

+44 DV993702_Pglauca .............

+65 GE474919_Pglauca ..................................................

+16 CO475409_Pglauca ..................................TC..............

-87 CO475124.2_Pglauca ..................................................

CONSENSUS ATCCTCACTATGGTATTAATATGGCTGGCTGTTTCAATGAAATATCCACT

+ Frame 2 I L T M V L I W L A V S M K Y P L

910 920 930 940 950

+65 GE474919_Pglauca ..........................................

+16 CO475409_Pglauca ..................................TC............

-87 CO475124.2_Pglauca ..................................................

CONSENSUS GCTCTTTTCTAGTCTCTCTCTATGTATCATTATTCAATGATTTCTTTTAT

+ Frame 2 L F S S L S L C I I I Q * F L L

960 970 980 990 1000

-87 CO475124.2_Pglauca ..................................................

CONSENSUS AGATATAGATAGTTTCTTATGCTGCATGCAGTGTAGTTTTTGGCATAAGC

+ Frame 2 * I * I V S Y A A C S V V F G I S

1010 1020 1030 1040 1050

-87 CO475124.2_Pglauca ..................................................

CONSENSUS TTCCATATTGAGCATGTATACTGAACAAGTTCAGAATAAAAAGCTTTGTA

+ Frame 2 F H I E H V Y * T S S E * K A L *

1060 1070 1080 1090 1100

-87 CO475124.2_Pglauca ..................................................

CONSENSUS ATTGATCAGAGTGCTGTTTTAGCCTCTATCAGTTTAGATTCTTCCAAAAC

+ Frame 2 L I R V L F * P L S V * I L P K

1110 1120 1130 1140 1150

-87 CO475124.2_Pglauca ..................................................

CONSENSUS GTAGCTCAGGTGCTTTTTTAAGGGGGCTTATGTAAGTAGTTGAGTCATTA

+ Frame 2 R S S G A F L R G L M * V V E S L

1160 1170 1180 1190 1200

-87 CO475124.2_Pglauca ..................................................

CONSENSUS TGGTTGTGAAATCAAGGCAGATGATCACTCATCACAATATATTGTCTGTC

+ Frame 2 W L * N Q G R * S L I T I Y C L S

1210 1220 1230 1240 1250

-87 CO475124.2_Pglauca ..................................................

CONSENSUS ATCTTAATAAGTGGTGATACTTTATACTTAATTATATAAGAAACATCTTC

+ Frame 2 S * * V V I L Y T * L Y K K H L

1260 1

-87 CO475124.2_Pglauca .................

CONSENSUS ATACTGTAATTGATCAG

+ Frame 2 H T V I D Q

1. ***Picea glauca CLE191***

10 20 30 40 50

+45 DV997954_Pglauca ..NNN.............................................

+66 GE475180_Pglauca ..................................................

+18 CO476765_Pglauca ..................................................

-17 CO475586_Pglauca ...........................................

No predicted signal peptide with either in-frame Met residue. Sequence likely not full-length

CONSENSUS GCTGCTGCAGGATTAACGTTCAGAGTACATTTGGTTTTCATGGCAGTAAT

+ Frame 1 A A A G L T F R V H L V F M A V M

60 70 80 90 100

+45 DV997954_Pglauca ..................................................

+66 GE475180_Pglauca ..................................................

+18 CO476765_Pglauca ..................................................

-17 CO475586_Pglauca ..............T...................................

CONSENSUS GGTGCTCTTTCCTGCTAATTCATTCGTGCAAGGATTGAGGCCCAGCTTCA

+ Frame 1 V L F P A N S F V Q G L R P S F

110 120 130 140 150

+45 DV997954_Pglauca ..................................................

+66 GE475180_Pglauca ..................................................

+18 CO476765_Pglauca ..................................................

-17 CO475586_Pglauca ..................................................

CONSENSUS TAACAAGAGATGTCCAAAACGTAGTAGGGCCCATGAAGAGTACAGAGTTG

+ Frame 1 I T R D V Q N V V G P M K S T E L

160 170 180 190 200

+45 DV997954_Pglauca ..................................................

+66 GE475180_Pglauca ..................................................

+18 CO476765_Pglauca ..................................................

-17 CO475586_Pglauca ..................................................

CONSENSUS CAGCAGCTGTCTGTAGTAAGTCCACTGAGGAATCTGATGGGGCAGAGGAC

+ Frame 1 Q Q L S V V S P L R N L M G Q R T

210 220 230 240 250

+45 DV997954_Pglauca ..................................................

+66 GE475180_Pglauca ..................................................

+18 CO476765_Pglauca ..................................................

-17 CO475586_Pglauca ..................................................

CONSENSUS CAATCTGCGGGAAGTCCTCCTGGGAAATCAGAAGGTCAACGCTGATCTTC

+ Frame 1 N L R E V L L G N Q K V N A D L

260 270 280 290 300

+45 DV997954_Pglauca ..................................................

+66 GE475180_Pglauca ..................................................

+18 CO476765_Pglauca ..................................................

-17 CO475586_Pglauca ..................................................

CONSENSUS AGGCTTCGAGTTTGAGAAGTGAAGATGGGGGAATGACAACAGCTTCTGCA

+ Frame 1 Q A S S L R S E D G G M T T A S A

310 320 330 340 350

+45 DV997954_Pglauca ..................................................

+66 GE475180_Pglauca ..................................................

+18 CO476765_Pglauca ..................................................

-17 CO475586_Pglauca ..................................................

CONSENSUS AGACAGGTCCCAACAGGCCCTGATCCTCTTCATCACAATGGCACCCCTGC

+ Frame 1 R Q V P T G P D P L H H N G T P A

360 370 380 390 400

+45 DV997954_Pglauca ..................................................

+66 GE475180_Pglauca ..................................................

+18 CO476765_Pglauca ..................................................

-17 CO475586_Pglauca ..................................................

CONSENSUS CAAGCCTGCATTTCCTCAACTTCCTTGAGTAGCCCAGCCAGGTATTATTG

+ Frame 1 K P A F P Q L P * V A Q P G I I

410 420 430 440 450

+45 DV997954_Pglauca ..................................................

+66 GE475180_Pglauca ..................................................

+18 CO476765_Pglauca ..................................................

-17 CO475586_Pglauca ..................................................

CONSENSUS ATTACAATATATGTTTGTAACAGCTATCTTCTGGCCAATATATTCCAATG

+ Frame 1 D Y N I C L * Q L S S G Q Y I P M

460 470 480 490 500

+45 DV997954_Pglauca ..................................................

+66 GE475180_Pglauca ..................................................

+18 CO476765_Pglauca ..................................................

-17 CO475586_Pglauca ..................................................

CONSENSUS TCTGTTTGTCTGTAAGCTGATCAAGAACAGGGGATTAATCAATGGCAAAA

+ Frame 1 S V C L * A D Q E Q G I N Q W Q K

510 520 530 540 550

+45 DV997954_Pglauca ..................................................

+66 GE475180_Pglauca ..................................................

+18 CO476765_Pglauca ..................................................

-17 CO475586_Pglauca ..................................................

CONSENSUS ATGATTGTAAGGTTGATATGTTTTGGAGCAGAATTCTAGTTACTTATTAA

+ Frame 1 * L * G * Y V L E Q N S S Y L L

560 570 580 590 600

+45 DV997954_Pglauca ..................................................

+66 GE475180_Pglauca ..................................................

+18 CO476765_Pglauca ..................................................

-17 CO475586_Pglauca ..................................................

CONSENSUS TAAAGTCTATGAGTCTGTGGCTTTCATGGCTGCTGCTTTCAGTTTTTGGG

+ Frame 1 I K S M S L W L S W L L L S V F G

610 620 630 640 650

+45 DV997954_Pglauca ..................................................

+66 GE475180_Pglauca ..................................................

+18 CO476765_Pglauca ..................................................

-17 CO475586_Pglauca ..................................................

CONSENSUS GATTGAAACCAGTTTTGTTTTTGGAGGTGGGACGCCATGAAGGACCTTAG

+ Frame 1 D * N Q F C F W R W D A M K D L S

660 670 680 690 700

+45 DV997954_Pglauca ..................................................

+66 GE475180_Pglauca ..................................................

+18 CO476765_Pglauca ..................................................

-17 CO475586_Pglauca ..................................................

CONSENSUS TACCAACATATGCTGTAATCTATTCTAGAAAGATATTTACATTTGTATAT

+ Frame 1 T N I C C N L F * K D I Y I C I

710 720 730 740 750

+45 DV997954_Pglauca ..................................................

+66 GE475180_Pglauca ..................................................

+18 CO476765_Pglauca ..................................................

-17 CO475586_Pglauca ..................................................

CONSENSUS ATATGGCGGACCTTTCCAGTCAATGAATTCTTTGTTTTGGTACTTGTCAT

+ Frame 1 Y M A D L S S Q * I L C F G T C H

760 770 780 790 800

+45 DV997954_Pglauca ...............NNN....N.NNNNNNN..NNNN...........NN

+66 GE475180_Pglauca ..................................................

+18 CO476765_Pglauca ..................................................

-17 CO475586_Pglauca .................................................G

CONSENSUS GTACACATATATACTGTTGTGCCACCTGGGAATGAATATTTCGATCATAA

+ Frame 1 V H I Y T V V P P G N E Y F D H N

810 820 830 840 850

+45 DV997954_Pglauca N.........NNNNNN.........NN....

+66 GE475180_Pglauca .................................

+18 CO476765_Pglauca .....................................T............

-17 CO475586_Pglauca .....................................*............

CONSENSUS TTTGCATCTGGGTCGATGCCTGAACAATCTATATTTT-CCATTCGCCACA

+ Frame 1 L H L G R C L N N L Y F - I R H

860

+18 CO476765_Pglauca ...............

-17 CO475586_Pglauca .

CONSENSUS TTTATACATACATTC

+ Frame 1 I Y T Y I

1. ***Picea glauca CLE192***

10 20 30 40 50

+19 CO481049_Pglauca .TT...............................................

-20 CO481288_Pglauca AG...............................................

CONSENSUS T--CATCTGTTCGTCTGTGTCCTGTTCGTGCTGTCGATTGTACTTTGCTC

+ Frame 1 - H L F V C V L F V L S I V L C S

60 70 80 90 100

+19 CO481049_Pglauca ..................................................

-20 CO481288_Pglauca ..................................................

-37 DR590195_Pglauca ..................

CONSENSUS CGCCGCAAGAGCACCGCCCTCGACTTTGTTCGACACATATGAGAGTAAGA

+ Frame 1 A A R A P P S T L F D T Y E S K

110 120 130 140 150

+19 CO481049_Pglauca ..................................................

-20 CO481288_Pglauca ..................................................

-37 DR590195_Pglauca ..................................................

CONSENSUS GCCGGAGCAGGTCGTCGGCTAATTTTCAGCCCTCTTTCAAGCTGCCCCAT

+ Frame 1 S R S R S S A N F Q P S F K L P H

160 170 180 190 200

+19 CO481049_Pglauca ..................................................

-20 CO481288_Pglauca ..................................................

-37 DR590195_Pglauca .....G............................................

CONSENSUS CCTCCCACTGAACAGGTAGATGCAGGCAGATTTGGAGTGGATAAACGACG

+ Frame 1 P P T E Q V D A G R F G V D K R R

210 220 230 240 250

+19 CO481049_Pglauca ..................................................

-20 CO481288_Pglauca ..................................................

-37 DR590195_Pglauca ..................................................

-87 CO240805_Pglauca .

CONSENSUS AGTCCCCACGGGCTCCAACCCCTTGCACAACAGGTAGCTGGGACGCCCTG

+ Frame 1 V P T G S N P L H N R * L G R P

260 270 280 290 300

+19 CO481049_Pglauca ..................................................

-20 CO481288_Pglauca ..................................................

-37 DR590195_Pglauca ..................................................

-87 CO240805_Pglauca ..................................................

CONSENSUS ATTGCCATTAATATGCCTTCTTGTGCCTGCAACTTCAATCGCAAACTTTC

+ Frame 1 D C H * Y A F L C L Q L Q S Q T F

310 320 330 340 350

+19 CO481049_Pglauca ..................................................

-20 CO481288_Pglauca ..................................................

-37 DR590195_Pglauca ..................................................

-87 CO240805_Pglauca ..................................................

CONSENSUS ACGGCAAGAGAATTATAGCTGTTTTTAATTCCTGATCTGCTAAATTAATA

+ Frame 1 T A R E L * L F L I P D L L N * Y

360 370 380 390 400

+19 CO481049_Pglauca ..................................................

-20 CO481288_Pglauca ..................................................

-37 DR590195_Pglauca ..................................................

-87 CO240805_Pglauca ..................................................

CONSENSUS CTTGGATATAGACAACTTAGGAAGAAATTAGAGTTGAATGAGAAGAAATT

+ Frame 1 L D I D N L G R N * S * M R R N

410 420 430 440 450

+19 CO481049_Pglauca ..................................................

-20 CO481288_Pglauca ..................................................

-37 DR590195_Pglauca ..................................................

-87 CO240805_Pglauca ..................................................

+88 EX355265_Pglauca ..........................................

CONSENSUS GCGCACAGTCGGCAATAGAAATGTAACATAGAGAGGATGTAATACATGGC

+ Frame 1 C A Q S A I E M * H R E D V I H G

460 470 480 490 500

+19 CO481049_Pglauca ..................................................

-20 CO481288_Pglauca ..................................................

-37 DR590195_Pglauca ..................................................

-87 CO240805_Pglauca ..................................................

+88 EX355265_Pglauca ..................................................

CONSENSUS TTGAAGAAAGTCCTGCTTGCGTTGACTCCACAACGTAACGCTGTCCGGCA

+ Frame 1 L K K V L L A L T P Q R N A V R Q

510 520 530 540 550

+19 CO481049_Pglauca ............................T.....................

-20 CO481288_Pglauca ............................T.....................

-37 DR590195_Pglauca ....................................C.............

-87 CO240805_Pglauca ....................................C.............

+88 EX355265_Pglauca ..................................................

CONSENSUS GATCCATGTTATTTGCAGCATCGTTTTTCATATTTATGGGCTTTGCAGAT

+ Frame 1 I H V I C S I V F H I Y G L C R

560 570 580 590 600

+19 CO481049_Pglauca ..................................................

-20 CO481288_Pglauca ..................................................

-37 DR590195_Pglauca ..................................................

-87 CO240805_Pglauca ..................................................

+88 EX355265_Pglauca ..................................................

CONSENSUS CCCATGCGCACCAAGGTGCAAGGTAAGGCTTTCGTTTTTAGACTGTGCTT

+ Frame 1 S H A H Q G A R * G F R F * T V L

610 620 630 640 650

+19 CO481049_Pglauca ..................................................

-20 CO481288_Pglauca ..................................................

-37 DR590195_Pglauca ..................................................

-87 CO240805_Pglauca ..................................................

+88 EX355265_Pglauca ..................................................

CONSENSUS TCAAGTTTTCGTAATCAGATTCCTGTTAGCTGCAGCAGGCCATCACGACA

+ Frame 1 S S F R N Q I P V S C S R P S R H

660 670 680 690 700

+19 CO481049_Pglauca ..................................................

-20 CO481288_Pglauca ..................................................

-37 DR590195_Pglauca ........................................T.........

-87 CO240805_Pglauca ..........................................G.......

+88 EX355265_Pglauca ..................................................

CONSENSUS CAATAATAACAACCTCGCGGTGACTGGGTAGTGGAATTTGCTCATCTCTG

+ Frame 1 N N N N L A V T G * W N L L I S

710 720 730 740 750

+19 CO481049_Pglauca ..................................................

-20 CO481288_Pglauca ..................................................

-37 DR590195_Pglauca ..................................................

-87 CO240805_Pglauca ...................................*..............

+88 EX355265_Pglauca ..................................................

CONSENSUS ATTATGAAGCCCCAGATCCGACGACAGTATATATGCCTTCATATATTATG

+ Frame 1 D Y E A P D P T T V Y M P S Y I M

760 770 780 790 800

+19 CO481049_Pglauca ..................................................

-20 CO481288_Pglauca ..................................................

-37 DR590195_Pglauca ..................................................

-87 CO240805_Pglauca ..................................................

+88 EX355265_Pglauca ..................................................

CONSENSUS TATATCGGGATTCCGAAACGACATTTCGAGGAGATGAAATCGTGCTTTCT

+ Frame 1 Y I G I P K R H F E E M K S C F L

810 820 830 840 850

+19 CO481049_Pglauca ..................................................

-20 CO481288_Pglauca ......................................A.G.........

-37 DR590195_Pglauca ..................................................

-87 CO240805_Pglauca ..................................................

+88 EX355265_Pglauca ..................................................

CONSENSUS GAACACTTCTGCATATTTATTGGATGTATCAGCTGTAATAATGATTCTTT

+ Frame 1 N T S A Y L L D V S A V I M I L

860 870 880 890 900

+19 CO481049_Pglauca .................

-20 CO481288_Pglauca .........................AAA

-37 DR590195_Pglauca ..................................................

-87 CO240805_Pglauca ..................................................

+88 EX355265_Pglauca .........................................

CONSENSUS CAAATATATTCATGTTTCAAATAAATCTTAAAACCAAAAAAAAAAAAAAA

+ Frame 1 S N I F M F Q I N L K T K K K K K

-37 DR590195_Pglauca ...

-87 CO240805_Pglauca .

CONSENSUS AAA

+ Frame 1 K

1. ***Picea glauca CLE193***

10 20 30 40 50

+92 DV973646_Pglauca ..................................................

+91 GE480021_Pglauca ..................................................

CONSENSUS CTGCTCCGCCTGATCAGATTTGAGCACGTCCTTCAATTGGGCAGAGGCCA

+ Frame 1 L L R L I R F E H V L Q L G R G H

60 70 80 90 100

+92 DV973646_Pglauca ..................................................

+91 GE480021_Pglauca ..................................................

CONSENSUS TGGAGAATGTGGGCATAACTGGCACACGGTTTCCAGATTAATTAATGAAT

+ Frame 1 G E C G H N W H T V S R L I N E

110 120 130 140 150

+92 DV973646_Pglauca ..................................................

+91 GE480021_Pglauca ..................................................

CONSENSUS ATCAGACCTTCTATTTTTATTATTATTCCATGGATGTGTGCAGTCCAAGC

+ Frame 1 Y Q T F Y F Y Y Y S M D V C S P S

160 170 180 190 200

+92 DV973646_Pglauca ..................................................

+91 GE480021_Pglauca ..................................................

CONSENSUS AACTTCGGAGGTGAGGCCAGTTGTTGAAGTCTTTAACAGTGCTAAATAAG

+ Frame 1 N F G G E A S C * S L * Q C * I R

210 220 230 240 250

+92 DV973646_Pglauca ..................................................

+91 GE480021_Pglauca ..................................................

CONSENSUS GAAGGCCTGAGCCTTGGATTGTTGGAGTTCAGAGGTGGCTTTCATTACAT

+ Frame 1 K A * A L D C W S S E V A F I T

260 270 280 290 300

+92 DV973646_Pglauca ..................................................

+91 GE480021_Pglauca ..................................................

CONSENSUS TGGGTTTTCATACCTGTCGGCGCTATACTTATTAGAAAGGTTTGAGCTTT

+ Frame 1 L G F H T C R R Y T Y * K G L S F

310 320 330 340 350

+92 DV973646_Pglauca ..................................................

+91 GE480021_Pglauca ..................................................

CONSENSUS GGATTCAAAAGGAGTTTTGATGCCTCTAGACTCTACACTGATCTGAAATT

+ Frame 1 G F K R S F D A S R L Y T D L K F

360 370 380 390 400

+92 DV973646_Pglauca ..................................................

+91 GE480021_Pglauca ..................................................

CONSENSUS TGATTAAAAAAAAGATTGAGTTTTGGCTTGCTGAAGAAGTTTTCATACCT

+ Frame 1 D * K K D * V L A C * R S F H T

410 420 430 440 450

+92 DV973646_Pglauca ..................................................

+91 GE480021_Pglauca ..................................................

CONSENSUS CTGGGACTGCATTATCAAGAATTTGATAAGAAGGCTAGAGGAGTTTTGAT

+ Frame 1 S G T A L S R I * * E G * R S F D

460 470 480 490 500

+92 DV973646_Pglauca ..................................................

+91 GE480021_Pglauca ..................................................

CONSENSUS ACCTCTCGACGCTACACTGATTAGAAATTTGATAAGAAGGCTGGAGCCTT

+ Frame 1 T S R R Y T D * K F D K K A G A L

510 520 530 540 550

+92 DV973646_Pglauca ..................................................

+91 GE480021_Pglauca ..................................................

CONSENSUS GGATTGCTCGAGGAGTTCAGAGGTGAATTTTAATACCTCTCAGTGCTACA

+ Frame 1 D C S R S S E V N F N T S Q C Y

560 570 580 590 600

+92 DV973646_Pglauca ..................................................

+91 GE480021_Pglauca ..................................................

CONSENSUS CTGATCAGAAATTTGGATCAGAAAAGCTTGAGCGTTGGATTGCTAGAAGA

+ Frame 1 T D Q K F G S E K L E R W I A R R

610 620 630 640 650

+92 DV973646_Pglauca ..................................................

+91 GE480021_Pglauca ..................................................

CONSENSUS ATTCAGAGGTGACTTTTATTACATCTGGTTTTAATACCTCTCTGGGCTAA

+ Frame 1 I Q R * L L L H L V L I P L W A K

660 670 680 690 700

+92 DV973646_Pglauca ..................................................

+91 GE480021_Pglauca ..................................................

CONSENSUS ACTGATAAGAAATTTGATCTGTGCTAGTCATATAAAGGATTTGATATTTT

+ Frame 1 L I R N L I C A S H I K D L I F

710 720 730 740 750

+92 DV973646_Pglauca ..................................................

+91 GE480021_Pglauca ..................................................

CONSENSUS TCCTGTACGTGATAGATATTAGAATCTTCATGAAAGTGGAGAGGATGAGA

+ Frame 1 F L Y V I D I R I F M K V E R M R

760 770 780 790 800

+92 DV973646_Pglauca ..................................................

+91 GE480021_Pglauca ..................................................

CONSENSUS ATATTGGGTCTCTTCGTGTCTGCCTTGGTATTGATTCTTCTGGTCTTGTC

+ Frame 1 I L G L F V S A L V L I L L V L S

810 820 830 840 850

+92 DV973646_Pglauca ..................N..N.NNNNN....N.NNN.N........NNN

+91 GE480021_Pglauca ..................................................

-23 CO486065_Pglauca ..............................

CONSENSUS TAATATATGTGGGGCTGAATCGTGGAGAATGCTCAGAAACACCAATGGCC

+ Frame 1 N I C G A E S W R M L R N T N G

860 870 880 890 900

+92 DV973646_Pglauca NNNNNN.......NNNNNNN....NNN.

+91 GE480021_Pglauca ..............................

-23 CO486065_Pglauca ..................................................

CONSENSUS GAGGATATCTTGAGAAGAATTCTGATGACCCACAGAGCCAAACCTTCAAT

+ Frame 1 R G Y L E K N S D D P Q S Q T F N

910 920 930 940 950

-23 CO486065_Pglauca ..................................................

CONSENSUS ATTCCAGCTCCTCACGCCTATGAATCTTATACCTACAGTCCACCAGATGA

+ Frame 1 I P A P H A Y E S Y T Y S P P D D

960 970 980 990 1000

-23 CO486065_Pglauca ..................................................

CONSENSUS TGGTAACGAGATTGATCCCAGGTATGGAGTGGAGAAGAGACTGGTTCCCA

+ Frame 1 G N E I D P R Y G V E K R L V P

1010 1020 1030 1040 1050

-23 CO486065_Pglauca ..................................................

-90 GE480075_Pglauca ............................

CONSENSUS CAGGTCCAAATCCTCTTCACCATTGAATATGGCCATGGAGTCATGGAGAT

+ Frame 1 T G P N P L H H * I W P W S H G D

1060 1070 1080 1090 1100

-23 CO486065_Pglauca ..................................................

-90 GE480075_Pglauca .........................................T........

-89 DR592314_Pglauca ..................................................

CONSENSUS CCAAAGCTCAAGACCAGGACCGTACATTAGTTCAGATCAATCTTCCGAGA

+ Frame 1 P K L K T R T V H * F R S I F R D

1110 1120 1130 1140 1150

-23 CO486065_Pglauca ..................................................

-90 GE480075_Pglauca .T.............................................T..

-89 DR592314_Pglauca ..................................................

CONSENSUS TCTGGGCTTCTATACAGATTCAGGAATATTGGTTCCTTAACATGGGTCTA

+ Frame 1 L G F Y T D S G I L V P * H G S

1160 1170 1180 1190 1200

-23 CO486065_Pglauca ..................................................

-90 GE480075_Pglauca ..................................................

-89 DR592314_Pglauca ..................................................

CONSENSUS CTGGTCATGGTCAATATAGAGCTGTTTGTTACAGGAAGAATTGCTATGTT

+ Frame 1 T G H G Q Y R A V C Y R K N C Y V

1210 1220 1230 1240 1250

-23 CO486065_Pglauca ..................................................

-90 GE480075_Pglauca ......................................T...........

-89 DR592314_Pglauca ..................................................

CONSENSUS TTTAAGGCTTCCAGACACTTTGATGGCAGCTATTGAATCTGAGGTTCAGA

+ Frame 1 F K A S R H F D G S Y * I * G S E

1260 1270 1280 1290 1300

-23 CO486065_Pglauca ..................................................

-90 GE480075_Pglauca ..................................................

-89 DR592314_Pglauca ..................................................

CONSENSUS GGCTTGAAGGCTTGAAGCTTTTGACACTTCAATGGCTGCTGGAGTCCATG

+ Frame 1 A * R L E A F D T S M A A G V H

1310 1320 1330 1340 1350

-23 CO486065_Pglauca ..................................................

-90 GE480075_Pglauca ................T.................................

-89 DR592314_Pglauca ..................................................

CONSENSUS ACTGTTATGACCATATCTTGGGTTGTTTATAATTGCCTTGAGATCAGGCT

+ Frame 1 D C Y D H I L G C L * L P * D Q A

1360 1370 1380 1390 1400

-23 CO486065_Pglauca ..................................................

-90 GE480075_Pglauca ..................................................

-89 DR592314_Pglauca ..................................................

CONSENSUS CAAGGCCTGTAGTTTTGGTTGTTATGCATTAATGCTTCATCAGAGTTCAA

+ Frame 1 Q G L * F W L L C I N A S S E F N

1410 1420 1430 1440 1450

-23 CO486065_Pglauca ..................................................

-90 GE480075_Pglauca ..................T...............................

-89 DR592314_Pglauca ..................................................

CONSENSUS TTACAGATAACAGAGAGTCTTAGATATTGATACTAGTGTTATACATATAT

+ Frame 1 Y R * Q R V L D I D T S V I H I

1460 1470 1480 1490 1500

-23 CO486065_Pglauca ..................................................

-90 GE480075_Pglauca ................T.........................T.....T.

-89 DR592314_Pglauca ..................................................

CONSENSUS TAGGTAATATACTGGTCTGATTTAGGAGCAGATGAGTCCAATCTCAATCT

+ Frame 1 L G N I L V * F R S R * V Q S Q S

1510 1520 1530 1540 1550

-23 CO486065_Pglauca ..................................................

-90 GE480075_Pglauca ..............T..................................T

-89 DR592314_Pglauca ..................................................

CONSENSUS CAGTTTTATTAATTCTTTGTAAGTAAGGAGAGCTGTACCTTCATTCATTC

+ Frame 1 Q F Y * F F V S K E S C T F I H S

1560 1570 1580 1590 1600

-23 CO486065_Pglauca ..................................................

-90 GE480075_Pglauca ............G........T.....................C......

-89 DR592314_Pglauca ..................................................

CONSENSUS TGAGGCCAAAGGCGAGTACTTCTGGTACTTGGAATAATAGATCACTGATA

+ Frame 1 E A K G E Y F W Y L E * * I T D

1610 1620 1630 1640 1650

-23 CO486065_Pglauca ..................................................

-90 GE480075_Pglauca ........*...TT.......G............................

-89 DR592314_Pglauca ..................................................

CONSENSUS ATTATATTCTTTC*TAATATGTGAATTGAATATAAGCTTTGTAATAACCA

+ Frame 1 N Y I L S N M * I E Y K L C N N Q

1660 1670 1680 1690 1700

-23 CO486065_Pglauca ..................................................

-90 GE480075_Pglauca ........A.................T....A.............

-89 DR592314_Pglauca ..................................................

CONSENSUS GAGGATCAGGGACATAATTTATATATGTATAGAGGATTAATTTCAAGCTT

+ Frame 1 R I R D I I Y I C I E D * F Q A

1710 1720 1730 1740 1750

-23 CO486065_Pglauca ......

-89 DR592314_Pglauca ..................................................

CONSENSUS GAATGCTGGTTTTCATGGCTGTTTTCATGAATAAAAAGAAAAAAAAAAAA

+ Frame 1 * M L V F M A V F M N K K K K K K

-89 DR592314_Pglauca ......

CONSENSUS AAAAAA

+ Frame 1 K K

1. ***Pinus taeda CLE194***

10 20 30 40 50

+26 DR160838_Ptaeda ..................................................

CONSENSUS ATTGCATACCAAGACTGGAAGACAGACAGTAATCACCATAACTGTGTGGA

+ Frame 1 I A Y Q D W K T D S N H H N C V E

60 70 80 90 100

+26 DR160838_Ptaeda ..................................................

CONSENSUS AACATATATAATTTTCTCTAATCAGAGATCATATTATTATAGGTTTTCAG

+ Frame 1 T Y I I F S N Q R S Y Y Y R F S

110 120 130 140 150

+26 DR160838_Ptaeda ..................................................

CONSENSUS GATAGTAACAGAAACAGAAACAGCCAGCCATGAGGGGATACTGTTTCATT

+ Frame 1 G * * Q K Q K Q P A M R G Y C F I

160 170 180 190 200

+26 DR160838_Ptaeda ..................................................

CONSENSUS GCGACCAGGCTTAGTAGAATGATTCTTTTCATCTTGCTCCTAGGAGTGTC

+ Frame 1 A T R L S R M I L F I L L L G V S

210 220 230 240 250

+26 DR160838_Ptaeda ..................................................

CONSENSUS AATGTCATTTAAGAATGAAAGGGCTCTTGGTATGAGAAATATAGAGAGGA

+ Frame 1 M S F K N E R A L G M R N I E R

260 270 280 290 300

+26 DR160838_Ptaeda ..................................................

CONSENSUS TAAAAGGAATTAGGCATGAGCTGTATGCAGTAGAAGCTGCTGCTACTGCT

+ Frame 1 I K G I R H E L Y A V E A A A T A

310 320 330 340 350

+26 DR160838_Ptaeda ..................................................

CONSENSUS ACTGAGGTTTCTCATGTGGATGCAAAAAACAGAGGAGGAGAAGCAGATTT

+ Frame 1 T E V S H V D A K N R G G E A D F

360 370 380 390 400

+26 DR160838_Ptaeda ..................................................

CONSENSUS CGATCTGAATTATGGGGATGTTAAGCGTAGAGTGCCCAATGGATCCGACC

+ Frame 1 D L N Y G D V K R R V P N G S D

410 420 430 440 450

+26 DR160838_Ptaeda ..................................................

CONSENSUS CAATTCATAATAGAAGAGCGGGGAAGTCAGGAGAGCCTCCAGCAGTATAA

+ Frame 1 P I H N R R A G K S G E P P A V *

460 470 480 490 500

+26 DR160838_Ptaeda ..................................................

CONSENSUS AGGATGAGATGATGCTGTGGCCTTCAACTATGAAGCAGAAATCAGAATGG

+ Frame 1 R M R * C C G L Q L * S R N Q N G

510 520 530 540 550

+26 DR160838_Ptaeda ..................................................

CONSENSUS AGTGCTTTTACATGAAAATACAGCATTGTAATCCATAAACGGAACTACCA

+ Frame 1 V L L H E N T A L * S I N G T T

560 570 580 590 600

+26 DR160838_Ptaeda ..................................................

CONSENSUS ATGCTAAGATGGTGGAGTGAATATTGTGATCTTTTCTCATTTTGGCATAA

+ Frame 1 N A K M V E * I L * S F L I L A *

610 620 630 640 650

+26 DR160838_Ptaeda ..................................................

CONSENSUS GGCGAGCTTATAATTACTTCTAGTGGTTTGGACATTGCCATGTGCAGAGA

+ Frame 1 G E L I I T S S G L D I A M C R D

660 670 680 690 700

+26 DR160838_Ptaeda ..................................................

CONSENSUS CAAAAAAAGAATAGAAGGAACAATTTTAGTTGTTACTGTAGATGTTCTTG

+ Frame 1 K K R I E G T I L V V T V D V L

710 720 730 740 750

+26 DR160838_Ptaeda ..................................................

CONSENSUS TTTTTCTTTTTACTATTCAATTAGTGTACAGAGAACAGATTGTTTGCTTG

+ Frame 1 V F L F T I Q L V Y R E Q I V C L

760 770 780 790 800

+26 DR160838_Ptaeda ..................................................

CONSENSUS ATTGCTTGGTAGACATTTCCTCCCTTACAAAAAAATAAATAAAGTTGTAT

+ Frame 1 I A W * T F P P L Q K N K * S C I

810

+26 DR160838_Ptaeda ...........

CONSENSUS AATTATTGGCC

+ Frame 1 I I G

1. ***Picea glauca CLE195***

10 20 30 40 50

+58 EX379750_Pglauca ..................................................

-59 EX380121_Pglauca ..................................................

CONSENSUS AGTAATTATCAGATTGAGGAGGGTTAGATTTTGGTCCAATCTTATGCAAG

+ Frame 3 * L S D * G G L D F G P I L C K

60 70 80 90 100

+58 EX379750_Pglauca ..................................................

-59 EX380121_Pglauca ..................................................

-61 EX386078_Pglauca .

+60 EX385719_Pglauca .

CONSENSUS CTATGCACAATGTTACTTACTTCCAGCGCCTCGTTTATGCTTCCTCGTGT

+ Frame 3 L C T M L L T S S A S F M L P R V

110 120 130 140 150

+58 EX379750_Pglauca ..................................................

-59 EX380121_Pglauca ..................................................

-61 EX386078_Pglauca ..................................................

+60 EX385719_Pglauca ..............-...................................

CONSENSUS GACAATTGTTCTTCAAGTCCTCTTGATCGTTACTGTTGTATGTGCCCATG

+ Frame 3 T I V L Q V L L I V T V V C A H

160 170 180 190 200

+58 EX379750_Pglauca ..................................................

-59 EX380121_Pglauca ..................................................

-61 EX386078_Pglauca ..................................................

+60 EX385719_Pglauca ..................................................

CONSENSUS AAATATCAGGATCGGTATTGGGTAAAGGAGGAAGGAATTCACTTCGTGCA

+ Frame 3 E I S G S V L G K G G R N S L R A

210 220 230 240 250

+58 EX379750_Pglauca ..................................................

-59 EX380121_Pglauca ..................................................

-61 EX386078_Pglauca ...............................T..................

+60 EX385719_Pglauca ...............................T..................

CONSENSUS AGATCGAGCTCTCAGGAATTTATAGCTCCTGCCAATGATAACAACACATA

+ Frame 3 R S S S Q E F I A P V/A N D N N T Y

260 270 280 290 300

+58 EX379750_Pglauca ..................................................

-59 EX380121_Pglauca ..................................................

-61 EX386078_Pglauca ..................................................

+60 EX385719_Pglauca ..................................................

CONSENSUS CAGAACTCTGCACCGGAAAATTACAAATGTGATGAGAGTAAGGAAAGTGG

+ Frame 3 R T L H R K I T N V M R V R K V

310 320 330 340 350

+58 EX379750_Pglauca ..................................................

-59 EX380121_Pglauca ..................................................

-61 EX386078_Pglauca ..................................................

+60 EX385719_Pglauca ..................................................

CONSENSUS ATTTTGATGTCAAAGATGGAGGATTCCAAAACCCTAGAGAAAATAATAAG

+ Frame 3 D F D V K D G G F Q N P R E N N K

360 370 380 390 400

+58 EX379750_Pglauca ..................................................

-59 EX380121_Pglauca ..................................................

-61 EX386078_Pglauca ..................................................

+60 EX385719_Pglauca ..................................................

CONSENSUS GGCAATACTGATCGACCATACGTCCAAAAGCTGCACAACGTCCCATCTGG

+ Frame 3 G N T D R P Y V Q K L H N V P S G

410 420 430 440 450

+58 EX379750_Pglauca ..................................................

-59 EX380121_Pglauca ..................................................

-61 EX386078_Pglauca ............T.....................................

+60 EX385719_Pglauca ............T.....................................

CONSENSUS GCCGAATCCGATAGGCAACTTTGATCCACCAGCACAGATTGACGGCCTCA

+ Frame 3 P N P I G N F D P P A Q I D G L

460 470 480 490 500

+58 EX379750_Pglauca ..................................................

-59 EX380121_Pglauca ..................................................

-61 EX386078_Pglauca ..................................................

+60 EX385719_Pglauca ..................................................

CONSENSUS GAGCTCCTTCTAGGCATTAATCTCAGGAGGTACAGTGTTCAAATGTACAG

+ Frame 3 R A P S R H * S Q E V Q C S N V Q

510 520 530 540 550

+58 EX379750_Pglauca ...............................T..................

-59 EX380121_Pglauca ...............................T..................

-61 EX386078_Pglauca ..................................................

+60 EX385719_Pglauca ..................................................

CONSENSUS CAACGTCTCTTGGGAATCGAATCGGATTTTACACACGAGAAAAAAACCCT

+ Frame 3 Q R L L G I E S D F T H E K K T L

560 570 580 590 600

+58 EX379750_Pglauca ..................................................

-59 EX380121_Pglauca ..................................................

-61 EX386078_Pglauca ..................................................

+60 EX385719_Pglauca ..................................................

CONSENSUS AAAAACCGGCAGAAAATATGAGGTAATAGTTCGTCAAAATGACATTGTCC

+ Frame 3 K T G R K Y E V I V R Q N D I V

610 620 630 640 650

+58 EX379750_Pglauca ..................................................

-59 EX380121_Pglauca ..................................................

-61 EX386078_Pglauca ..................................................

+60 EX385719_Pglauca ..................................................

CONSENSUS AGGCAGATACATGCACTGAATCGACGGTATGAGTCATAGTTAGCCAGATG

+ Frame 3 Q A D T C T E S T V * V I V S Q M

660 670 680 690 700

+58 EX379750_Pglauca ..................................................

-59 EX380121_Pglauca ..................................................

-61 EX386078_Pglauca ..................................................

+60 EX385719_Pglauca ..................................................

CONSENSUS ATTTTCTATAAATCAGCGACTGTTTTGTATTATGCGACTACGAATCTTAC

+ Frame 3 I F Y K S A T V L Y Y A T T N L T

710 720 730 740 750

+58 EX379750_Pglauca ..................................................

-59 EX380121_Pglauca ..................................................

-61 EX386078_Pglauca ..................................................

+60 EX385719_Pglauca ..................................................

CONSENSUS TTATTTCATGTACTTACAGATTACCTAAAATAAAGTTTGGTGTACGTCTA

+ Frame 3 Y F M Y L Q I T * N K V W C T S

760 770 780 790 800

+58 EX379750_Pglauca ..................................................

-59 EX380121_Pglauca ..................................................

-61 EX386078_Pglauca ..................................................

+60 EX385719_Pglauca ..................................................

CONSENSUS TATCAATCATTTAGTAACAAAATGCTAAATGTCAATTAATGATGTGACAC

+ Frame 3 I S I I * * Q N A K C Q L M M * H

810 820 830

+58 EX379750_Pglauca ....................................

-59 EX380121_Pglauca ....................................

-61 EX386078_Pglauca ....................................

+60 EX385719_Pglauca ....................................

CONSENSUS TTTAAATTATTAAATCACTTTTAATTTTTCAAAAAA

+ Frame 3 F K L L N H F * F F K K

1. ***Picea engelmannii × glauca CLE196***

10 20 30 40 50

-29 DR464285_PengelxPgla ..................................................

CONSENSUS GGACAGGTATTTATGTTTGCAGTAAAAATAATTCTATAACATATATGCTA

+ Frame 1 G Q V F M F A V K I I L * H I C *

60 70 80 90 100

-29 DR464285_PengelxPgla ..................................................

CONSENSUS GCATATCCGGCTCTGTAAAATCTATATCATAAAGTTTGGTAATAAAATAA

+ Frame 1 H I R L C K I Y I I K F G N K I

110 120 130 140 150

-29 DR464285_PengelxPgla ..................................................

CONSENSUS GAGAAGAGCTATTCTCAGTCTCTGTCAATGCAGGGATTTATTATCTAAAA

+ Frame 1 R E E L F S V S V N A G I Y Y L K

160 170 180 190 200

-29 DR464285_PengelxPgla ..................................................

CONSENSUS CATTGGGATTTTAAGTGTATTCAGGAAGCTTGATTCGATTTGGATTTCTC

+ Frame 1 H W D F K C I Q E A * F D L D F S

210 220 230 240 250

-29 DR464285_PengelxPgla ..................................................

CONSENSUS TCTAATGGCGAAAGGATGTAATTACTGCAAGATGGGGGCAGTACTTTCTT

+ Frame 1 L M A K G C N Y C K M G A V L S

260 270 280 290 300

-29 DR464285_PengelxPgla ..................................................

CONSENSUS TCATTACTGTTCTGTATATTTTGGTGGCCTTATCGGCTAACTCTGTGGAA

+ Frame 1 F I T V L Y I L V A L S A N S V E

310 320 330 340 350

-29 DR464285_PengelxPgla ..................................................

CONSENSUS GCAATGAGTAGCAATAAAGCCTATCCTAACATTCAGCCCCCTTGTGCATC

+ Frame 1 A M S S N K A Y P N I Q P P C A S

360 370 380 390 400

-29 DR464285_PengelxPgla ..................................................

CONSENSUS TGCTGGTACAAAGGAATATTGTTCTCATGTGCCATGCAGACATGCACCAG

+ Frame 1 A G T K E Y C S H V P C R H A P

410 420 430 440 450

-29 DR464285_PengelxPgla ..................................................

CONSENSUS ATGACGTGTCTGGCAGATTTGGAGCTCAAAAGAGAAGGGTTCCTACGGGT

+ Frame 1 D D V S G R F G A Q K R R V P T G

460 470 480 490 500

-29 DR464285_PengelxPgla ..................................................

CONSENSUS CCTAATCCGTTACATAACTGACCCCAACAAATGGGTTCAAAGAGTTACAG

+ Frame 1 P N P L H N * P Q Q M G S K S Y R

510 520 530 540 550

-29 DR464285_PengelxPgla ..................................................

CONSENSUS GAGCCAACCATGTCTTGCAATCCTGGGCAGGCAGGCCATGGACTCTCCCA

+ Frame 1 S Q P C L A I L G R Q A M D S P

560 570 580 590 600

-29 DR464285_PengelxPgla ..................................................

CONSENSUS TCAAAATATATGTATGGATAGATAGCGAATAACCTGATAAATACAGCAGA

+ Frame 1 I K I Y V W I D S E * P D K Y S R

610 620 630 640 650

-29 DR464285_PengelxPgla ..................................................

CONSENSUS ATCTCTCTGCAATAGGAAATGTATGTATGGATAGATAGCGAATAACCTGA

+ Frame 1 I S L Q * E M Y V W I D S E * P D

660 670 680 690 700

-29 DR464285_PengelxPgla ..................................................

CONSENSUS TAAATACAGCAGAATCTCTCTGCAATAGGATGCTAATCTATCTATCTCTG

+ Frame 1 K Y S R I S L Q * D A N L S I S

710 720 730 740 750

-29 DR464285_PengelxPgla ..................................................

CONSENSUS CAGATTGGAAACAGAAAGTAGGTGTCGTACGCCTCTCTTTTATAATTTAG

+ Frame 1 A D W K Q K V G V V R L S F I I *

760 770 780 790 800

-29 DR464285_PengelxPgla ..................................................

CONSENSUS TGATGGGTATTTTGTAATTTGATGCAGTGTCGAATGTAGCTATAAAAAAA

+ Frame 1 * W V F C N L M Q C R M * L * K K

810

-29 DR464285_PengelxPgla ...........

CONSENSUS AAAAAAAAAAA

+ Frame 1 K K K

1. ***Picea sitchensis CLE197***

10 20 30 40 50

+32 DR483224_Psitchensis ..................................................

CONSENSUS GAATGATATGAGGAACGGTTGAATTGGGATTATAGGGCAGGATATGACTA

+ Frame 3 M I * G T V E L G L * G R I * L

60 70 80 90 100

+32 DR483224_Psitchensis ..................................................

CONSENSUS CTTGGGCCTCAATATCAGCCTTCAGAGTTTCAAGCTGTGATTTATCATCT

+ Frame 3 L G P Q Y Q P S E F Q A V I Y H L

110 120 130 140 150

+32 DR483224_Psitchensis ..................................................

CONSENSUS CAAGCATTGGAGTGTATTCAGAAAGCTTGATTTGATTTGGGTGAAGTCTC

+ Frame 3 K H W S V F R K L D L I W V K S

160 170 180 190 200

+32 DR483224_Psitchensis ..................................................

CONSENSUS TAATGGGGAAAAGATGGAATTGCTACAAGGTAGGAGCAGTGGTTTCTTTG

+ Frame 3 L M G K R W N C Y K V G A V V S L

210 220 230 240 250

+32 DR483224_Psitchensis ..................................................

-31 DR477572_Psitchensis ..............................................

CONSENSUS ATTACTGCCATGTATGTTTTGGCGGTCTTATCAGCTGATTGTGTAGAAGC

+ Frame 3 I T A M Y V L A V L S A D C V E A

260 270 280 290 300

+32 DR483224_Psitchensis ..................................................

-31 DR477572_Psitchensis ..................................................

CONSENSUS AATGAGGACCAATACAGCCTATAATTACATTCAGGGACCTCCTGCATCAG

+ Frame 3 M R T N T A Y N Y I Q G P P A S

310 320 330 340 350

+32 DR483224_Psitchensis ..................................................

-31 DR477572_Psitchensis ..................................................

CONSENSUS ATGCAACAACGCAGTATTGTTCTCATGTTTCATGCAGAGATGCAGCAGCT

+ Frame 3 D A T T Q Y C S H V S C R D A A A

360 370 380 390 400

+32 DR483224_Psitchensis ..................................................

-31 DR477572_Psitchensis ..................................................

CONSENSUS GAAATGGAGCCCGGATTTGGAGATCAAAAGCGAACGGTTCCTACTGGTTC

+ Frame 3 E M E P G F G D Q K R T V P T G S

410 420 430 440 450

+32 DR483224_Psitchensis ..................................................

-31 DR477572_Psitchensis ..................................................

CONSENSUS TAATCCGTTGCATAACTGACAGAAAACAAATGTATTCAAAGAGTTACAGG

+ Frame 3 N P L H N * Q K T N V F K E L Q

460 470 480 490 500

+32 DR483224_Psitchensis ..................................................

-31 DR477572_Psitchensis ..................................................

CONSENSUS AGCCAACAATGCTGTACATTCATGGTGTCTTCTCTTTTTCTCTCTATCTC

+ Frame 3 E P T M L Y I H G V F S F S L Y L

510 520 530 540 550

+32 DR483224_Psitchensis ..................................................

-31 DR477572_Psitchensis ..................................................

CONSENSUS TCTGTATAGGATTATCAGATTATTCTTTCAGTATTTGTACGAAGAAACTG

+ Frame 3 S V * D Y Q I I L S V F V R R N *

560 570 580 590 600

+32 DR483224_Psitchensis ..................................................

-31 DR477572_Psitchensis ..................................................

CONSENSUS ATAAATACAGCCATGGCAGGCAGTTAGGTGCCCATGTTCCTTTTTAGATA

+ Frame 3 * I Q P W Q A V R C P C S F L D

610 620 630 640 650

+32 DR483224_Psitchensis ..................................................

-31 DR477572_Psitchensis ..................................................

CONSENSUS ATATTTGATTGTCAGGTTATTCTTGATGTATTTATAGGAATACCGTGATA

+ Frame 3 N I * L S G Y S * C I Y R N T V I

660 670 680 690 700

+32 DR483224_Psitchensis .................................A.............

-31 DR477572_Psitchensis .................................T................

CONSENSUS AATACAGCAAGAATCTCTGTAATAGGATAATGG-ATATTTCTGTTTATAA

+ Frame 3 N T A R I S V I G * W - I S V Y N

710 720 730 740 750

-31 DR477572_Psitchensis ..................................................

CONSENSUS TCTATTTTTAAGGTCACAGCTAAGCCAGATCAAATATGAGACCTCTTCGT

+ Frame 3 L F L R S Q L S Q I K Y E T S S

760 770 780 790 800

-31 DR477572_Psitchensis ..................................................

CONSENSUS TCAGCTATTTTGAATGATAAAGTATAAGAAGGTATATATATCCTATCCTC

+ Frame 3 F S Y F E * * S I R R Y I Y P I L

810 820 830 840 850

-31 DR477572_Psitchensis ..................................................

CONSENSUS TCTAGATTTGAACTTGTAAACTCTGACGAGGAAAAATTGGAAACAGAAAA

+ Frame 3 S R F E L V N S D E E K L E T E N

860 870 880 890 900

-31 DR477572_Psitchensis ..................................................

CONSENSUS TAGTTCTGATACACAGGTTTGGCCATCTTGTAATTGATTTCCATATGGTG

+ Frame 3 S S D T Q V W P S C N * F P Y G

910 920 930 940 950

-31 DR477572_Psitchensis ..................................................

CONSENSUS AATTGTATTAAAAATCTTTATTTGATTTCCATATGGTGCATTGTATTAAA

+ Frame 3 E L Y * K S L F D F H M V H C I K

960 970 980 990 1000

-31 DR477572_Psitchensis ..................................................

CONSENSUS AATCTTTATTGTAACAGAATAAACGAGTGGAGGGACAGATTTGAGCCCTT

+ Frame 3 N L Y C N R I N E W R D R F E P L

1010 1020

-31 DR477572_Psitchensis .........................

CONSENSUS GTATTGCGTAAAAAAAAAAAAAAAA

+ Frame 3 Y C V K K K K K

1. ***Pinus contorta CLE198***

10 20 30 40 50

+83 GT246831_Pcontorta ..........................................TT....

+76 GT239220_Pcontorta ....

CONSENSUS GGGGGTTGCTTCTTGTCTCCCATTTTCTTCGCCCGCAGGACGAGTTGAAC

+ Frame 2 G V A S C L P F S S P A G R - E

60 70 80 90 100

+83 GT246831_Pcontorta ..................................................

+76 GT239220_Pcontorta ..................................................

CONSENSUS ACAGAAGGATTTGGGAGAAAATTCAGGAAACGGCGTGTCAGAATTAGATC

+ Frame 2 H R R I W E K I Q E T A C Q N * I

110 120 130 140 150

+83 GT246831_Pcontorta ..................................................

+76 GT239220_Pcontorta ..................................................

CONSENSUS CAATGATAAGGCAATGAAGAAATAATTATGACAGTTTGAATATAATTTCA

+ Frame 2 Q * * G N E E I I M T V * I * F H

160 170 180 190 200

+83 GT246831_Pcontorta ..................................................

+76 GT239220_Pcontorta ..................................................

CONSENSUS TGGTTTTGTTTGTTTTTGTAGAGGGAAGCAGGGGATTTTGAACTTGATTT

+ Frame 2 G F V C F C R G K Q G I L N L I

210 220 230 240 250

+83 GT246831_Pcontorta ..................................................

+76 GT239220_Pcontorta ..................................................

CONSENSUS TCTTTGGCTTACATAATACTTTATGGGATGGCCAATTGAGCTTTCATGGT

+ Frame 2 F F G L H N T L W D G Q L S F H G

260 270 280 290 300

+83 GT246831_Pcontorta ..................................................

+76 GT239220_Pcontorta ..................................................

CONSENSUS TTTGCATAAGGTTGGTTCGAAATAAGAGATTTTAAATTTGAATTTCTTGG

+ Frame 2 F A * G W F E I R D F K F E F L G

310 320 330 340 350

+83 GT246831_Pcontorta ..........G.......................................

+76 GT239220_Pcontorta ..........A.......................................

CONSENSUS TTTACATAAT-CTTGATAGCATGGCGATCACCAACTGACCTGCCCGGATA

+ Frame 2 L H N - * * H G D H Q L T C P D

360 370 380 390 400

+83 GT246831_Pcontorta .G...................................G............

+76 GT239220_Pcontorta .A...................................A............

CONSENSUS G-CTTGAGAAACTCATAACATTAACCGTGGGGTATTG-TATTCTTTGCTT

+ Frame 2 - L E K L I T L T V G Y - Y S L L

410 420 430 440 450

+83 GT246831_Pcontorta ..................................................

+76 GT239220_Pcontorta ..................................................

CONSENSUS CAATAAATTGTTGCATTCCAGGCTTTGAGACTTGGATGGATTTGGGTTTC

+ Frame 2 Q * I V A F Q A L R L G W I W V S

460 470 480 490 500

+83 GT246831_Pcontorta ..................................................

+76 GT239220_Pcontorta ..................................................

CONSENSUS GCTAGGCATCTTACAGGGGGCAGTCTGTATGGCGGATGGTTTTGTTAGAA

+ Frame 2 L G I L Q G A V C M A D G F V R

510 520 530 540 550

+83 GT246831_Pcontorta ...................................G..............

+76 GT239220_Pcontorta ...................................A..............

CONSENSUS GGCTTAAAAGGGCAGATAAAAAGTTGCTGCTCATA-TTTTTCTGTTGTTG

+ Frame 2 R L K R A D K K L L L I V/I F L L L

560 570 580 590 600

+83 GT246831_Pcontorta ..................................................

+76 GT239220_Pcontorta ..................................................

CONSENSUS GGTCTCTTCTTTCAGCGAGTAGATTCAACACCATGCCCAGGAAAAAAGTT

+ Frame 2 G L F F Q R V D S T P C P G K K L

610 620 630 640 650

+83 GT246831_Pcontorta ................................................

+76 GT239220_Pcontorta ..................................................

CONSENSUS GCAGGATTTTGGTCATGGAGAATTAGGGGTTACATTGAAGCATTCTCCAT

+ Frame 2 Q D F G H G E L G V T L K H S P

660 670 680 690 700

+76 GT239220_Pcontorta ..................................................

CONSENSUS GTCGAAGGATCTTGGCTTCCAGGGAATTCTTTGTTCCCAAGCACTCTAAG

+ Frame 2 C R R I L A S R E F F V P K H S K

710 720 730 740 750

+76 GT239220_Pcontorta ..................................................

CONSENSUS GGCTCCAGAACACATTATGAGGAAAGCGATCATGAAGTTCCCAGCGGTCC

+ Frame 2 G S R T H Y E E S D H E V P S G P

760 770

+76 GT239220_Pcontorta ....................

CONSENSUS AAATCCCATTAGTAATAGGT

+ Frame 2 N P I S N R

1. ***Picea glauca CLE199***

10 20 30 40 50

+50 EX317283_Pglauca ..................................................

CONSENSUS AACCGGAACGATTTGGGAGAAAATTCAGGAAACTGCCCGTCAGAATTCGA

+ Frame 3 P E R F G R K F R K L P V R I R

60 70 80 90 100

+50 EX317283_Pglauca ..................................................

CONSENSUS TCCAATGATAAGGAAATGAAGAGATAATTATGACAGTTTGAAATAATTTC

+ Frame 3 S N D K E M K R * L * Q F E I I S

110 120 130 140 150

+50 EX317283_Pglauca ..................................................

CONSENSUS ATGGTTTTATCCGGTTTTGCAGAGGTTGAAGTGCGGGGGGTATTCTTTGC

+ Frame 3 W F Y P V L Q R L K C G G Y S L

160 170 180 190 200

+50 EX317283_Pglauca ..................................................

CONSENSUS TTCAATAAATAGTTATATTCCAGGCTTCGAAAGTTGAATGGATTTGGGTT

+ Frame 3 L Q * I V I F Q A S K V E W I W V

210 220 230 240 250

+50 EX317283_Pglauca ..................................................

CONSENSUS TCGTTAGGCATCTTACAGGGGGCAGTCTGTATGGCGGATGGTTTTGTTAG

+ Frame 3 S L G I L Q G A V C M A D G F V R

260 270 280 290 300

+50 EX317283_Pglauca ..................................................

CONSENSUS AAGGCTAAAAAAGGCAGACAAAAACTTGCTGGTGGTAGTTTTTCTCTTCT

+ Frame 3 R L K K A D K N L L V V V F L F

310 320 330 340 350

+50 EX317283_Pglauca ..................................................

-51 EX317636_Pglauca ...................

CONSENSUS TGGTTCTCTTCTTTCAGCGCGTGGATCCAACGCCATGCCCAGAAAAAGAG

+ Frame 3 L V L F F Q R V D P T P C P E K E

360 370 380 390 400

+50 EX317283_Pglauca .............G....................................

-51 EX317636_Pglauca .............T....................................

CONSENSUS TTACAGAATATTGKTCCTGGAGGATTAAGGGTTATATTGAAGCATCCCCC

+ Frame 3 L Q N I G/V P G G L R V I L K H P P

410 420 430 440 450

+50 EX317283_Pglauca .............TC...................................

-51 EX317636_Pglauca .............CG...................................

CONSENSUS ATGTAGAAGGCTTYSGGCCTCTCGGGAGTTCTTCGTTCCCAAGCACAATA

+ Frame 3 C R R L S/R A S R E F F V P K H N

460 470 480 490 500

+50 EX317283_Pglauca ..................................................

-51 EX317636_Pglauca ..................................................

CONSENSUS AGGGCTCCAGAAGAACAAGTTATGGTGCAAGCGATCATGAAGTTCCCAGC

+ Frame 3 K G S R R T S Y G A S D H E V P S

510 520 530 540 550

+50 EX317283_Pglauca ..................................................

-51 EX317636_Pglauca ..................................................

CONSENSUS GGTCCAAATCCAATAAGTAATCGGTAAGGAATTAATGCAGGTGGGAGCGA

+ Frame 3 G P N P I S N R * G I N A G G S D

560 570 580 590 600

+50 EX317283_Pglauca ..................................................

-51 EX317636_Pglauca ..................................................

CONSENSUS TGCTCATGAGTTCATGATCGGGCATTGATTATTAAATTTCCATTTCGGAT

+ Frame 3 A H E F M I G H * L L N F H F G

610 620 630 640 650

+50 EX317283_Pglauca ..................................................

-51 EX317636_Pglauca ..................................................

CONSENSUS TCTGATTCAGATGGTTCTTCTTATGGTTAATTTAGATTAAATGCAAAATT

+ Frame 3 F * F R W F F L W L I * I K C K I

660 670 680 690 700

+50 EX317283_Pglauca ..................................................

-51 EX317636_Pglauca ..................................................

CONSENSUS GGGTGTTAATAACTTTTCTACGGCAATGGCATAGTCGAATATGTCAGATC

+ Frame 3 G C * * L F Y G N G I V E Y V R S

710 720 730 740 750

+50 EX317283_Pglauca ..................................................

-51 EX317636_Pglauca ..................................................

CONSENSUS GGATCGGCTTTCTGTGCTGTGTTTTGATAAAGAGCTCAGAGTTTCGGGCT

+ Frame 3 D R L S V L C F D K E L R V S G

760 770 780 790 800

+50 EX317283_Pglauca ................A......*..........................

-51 EX317636_Pglauca ................*......A..........................

CONSENSUS TCGAAGTTTCGTCGGA-TAGAGT-AACTAATATTTCTCAATGTCCATGGC

+ Frame 3 F E V S S - * S - L I F L N V H G

810 820 830 840 850

+50 EX317283_Pglauca ..................................................

-51 EX317636_Pglauca ..................................................

CONSENSUS CTGGCCATGGCAGAGCTCTTTCGGCACTTGCGATACGGTATATTATCAGT

+ Frame 3 L A M A E L F R H L R Y G I L S V

860 870 880 890 900

+50 EX317283_Pglauca .............G....................................

-51 EX317636_Pglauca .............A....................................

CONSENSUS TAAAGATATAGGG-AATATTTTAAAGAGATAGCCAGTACTTCTCTTTAAA

+ Frame 3 K D I G - Y F K E I A S T S L *

910 920 930 940 950

+50 EX317283_Pglauca .

-51 EX317636_Pglauca ..................................................

CONSENSUS TTAGCCATTGCCAGAAGATCATACTATTCTTATTATCTTCATTTGTTTGT

+ Frame 3 I S H C Q K I I L F L L S S F V C

960 970 980 990 1000

-51 EX317636_Pglauca ..................................................

CONSENSUS CAGGCATCTGTATTCTTAGGTTTCCTGTAGGAAAAAAGAAAAAGAAAAAG

+ Frame 3 Q A S V F L G F L * E K R K R K S

1010 1020 1030 1040 1050

-51 EX317636_Pglauca ..................................................

CONSENSUS TAGTCAGGGAGGATTGAGAAAAGGGTATGAGAATATTGAGCTGTGCTTGC

+ Frame 3 S Q G G L R K G Y E N I E L C L

1060 1070 1080 1090 1100

-51 EX317636_Pglauca ..................................................

CONSENSUS TGCAGCTATAAGAACTTTTGTATGTATTTTTCTTTATGAATGGCCAAATT

+ Frame 3 L Q L * E L L Y V F F F M N G Q I

1110 1120 1130 1140 1150

-51 EX317636_Pglauca ..................................................

CONSENSUS ATAGACTGATCTATTAACTTTCTACAGCAGTATAGATTGATGGAATTGGC

+ Frame 3 I D * S I N F L Q Q Y R L M E L A

1160 11

-51 EX317636_Pglauca ..................

CONSENSUS CCAGTGTTTGCCAAAAAA

+ Frame 3 Q C L P K -

1. ***Picea glauca CLE200***

10 20 30 40 50

+56 EX368883_Pglauca ..................................................

+63 EX402265_Pglauca ...................

CONSENSUS ATGAGAGGCTGGATGCCCAATTAATTGAAGCAATGATATGAGGAACGGTT

+ Frame 3 E R L D A Q L I E A M I * G T V

60 70 80 90 100

+56 EX368883_Pglauca ..................................................

+63 EX402265_Pglauca ..................................................

CONSENSUS GAATTGGGATTATAGCCCAGGATATGACTACTTGGGCCTCAATATCAGCC

+ Frame 3 E L G L * P R I * L L G P Q Y Q P

110 120 130 140 150

+56 EX368883_Pglauca ..................................................

+63 EX402265_Pglauca ..................................................

+55 EX358925_Pglauca ...........................................

CONSENSUS TTCAGAGTTTCAAGCTGTGATTTATCATCTCAAGCATTGGAGTGTATTCA

+ Frame 3 S E F Q A V I Y H L K H W S V F

160 170 180 190 200

+56 EX368883_Pglauca ..................................................

+63 EX402265_Pglauca ..................................................

+55 EX358925_Pglauca ...............................................G..

CONSENSUS GAAAGCTTGATTTGATTTGGGTGAAGTCTCTAATGGGGAAAAGATGGAAT

+ Frame 3 R K L D L I W V K S L M G K R W N

210 220 230 240 250

+56 EX368883_Pglauca ..................................................

+63 EX402265_Pglauca ..................................................

+55 EX358925_Pglauca ..........................T.......................

CONSENSUS TGCTACAAGGTAGGAGCAGTGGTTTCCTTGATTACTGCCATGTATGTTTT

+ Frame 3 C Y K V G A V V S L I T A M Y V L

260 270 280 290 300

+56 EX368883_Pglauca ...........................G......................

+63 EX402265_Pglauca ..................................................

+55 EX358925_Pglauca ..................................................

CONSENSUS GGCGGTCTTATCAGCTGATTGTGTGGAAGCAATGAGGACCAATACAGCCT

+ Frame 3 A V L S A D C V E A M R T N T A

310 320 330 340 350

+56 EX368883_Pglauca ...............................C..................

+63 EX402265_Pglauca ..................................................

+55 EX358925_Pglauca ..................................................

CONSENSUS ATAATTACATTCAGGGACCTCCTGCATCAGATGCAACAACGCAGTATTGT

+ Frame 3 Y N Y I Q G P P A S D A T T Q Y C

360 370 380 390 400

+56 EX368883_Pglauca ..................................................

+63 EX402265_Pglauca ..................................................

+55 EX358925_Pglauca ......................................G...........

CONSENSUS TCTCATGTTTCATGCAGAGATGCAGCAGCTGAAATGGAACCCGGATTTGG

+ Frame 3 S H V S C R D A A A E M E P G F G

410 420 430 440 450

+56 EX368883_Pglauca ..................................................

+63 EX402265_Pglauca ..................................................

+55 EX358925_Pglauca ..................................................

CONSENSUS AGATCAAAAGCGAACGGTTCCTACTGGTTCTAATCCGTTGCATAACTGAC

+ Frame 3 D Q K R T V P T G S N P L H N *

460 470 480 490 500

+56 EX368883_Pglauca ........-----------------------------.............

+63 EX402265_Pglauca ..................................................

+55 EX358925_Pglauca ..................................................

CONSENSUS AGAAAACAAATGTATTCAAAGAGTTACAGGAGCCAACAATGCTGTACATT

+ Frame 3 Q K T N V F K E L Q E P T M L Y I

510 520 530 540 550

+56 EX368883_Pglauca ..................................................

+63 EX402265_Pglauca ..................................................

+55 EX358925_Pglauca ..................................................

CONSENSUS CATGGTGTCTTCTCTTTTTCTCTCTATCTCTCTGTATAGGATTATCAGAT

+ Frame 3 H G V F S F S L Y L S V * D Y Q I

560 570 580 590 600

+56 EX368883_Pglauca ..................................................

+63 EX402265_Pglauca ..................................................

+55 EX358925_Pglauca ..................................................

CONSENSUS TATTCTTTCAGTATTTGTACGAAGAAACTGATAAATACAGCCATGGCAGG

+ Frame 3 I L S V F V R R N * * I Q P W Q

610 620 630 640 650

+56 EX368883_Pglauca ..................................................

+63 EX402265_Pglauca .........................G..A..A.

+55 EX358925_Pglauca ............................................N.....

CONSENSUS CAGGTAGGTGCCCATGTTCCTTTTTAGATAATATTTGATTATCAGGTTAT

+ Frame 3 A G R C P C S F L D N I * L S G Y

660 670 680 690 700

+56 EX368883_Pglauca ..................................................

+55 EX358925_Pglauca ..................................................

CONSENSUS TCTTGATGTATTTATAGGAATACCGTGATAAATACAGCAAGAATCTCTGT

+ Frame 3 S * C I Y R N T V I N T A R I S V

710

+56 EX368883_Pglauca ............

CONSENSUS AATAGGAAAAAA

+ Frame 3 I G K -

1. ***Pinus contorta CLE201***

10 20 30 40 50

+80 GT230706_Pcontorta ..................................................

CONSENSUS GGGGGGTGCATTAAATTCATGTAAATAAGGAGTTCAGGTACTTTGTGAGT

+ Frame 3 G V H * I H V N K E F R Y F V S

60 70 80 90 100

+80 GT230706_Pcontorta ..................................................

CONSENSUS AGAAGATTCACCAGTAAGAAGATACGGGGAAAGGGATTAATTTCAAAGCA

+ Frame 3 R R F T S K K I R G K G L I S K Q

110 120 130 140 150

+80 GT230706_Pcontorta ..................................................

CONSENSUS GAGGCTGAGATGCAGCAGTGGTGGAAGATTTAATGGAAGAGCATGAAGCC

+ Frame 3 R L R C S S G G R F N G R A * S

160 170 180 190 200

+80 GT230706_Pcontorta ..................................................

+53 GT258781_Pcontorta --......................................

CONSENSUS CCGTGAGCTTTCAAGGCTGTAAATTCATCTTTTGCCTTTGCATTAACGAC

+ Frame 3 P V S F Q G C K F I F C L C I N D

210 220 230 240 250

+80 GT230706_Pcontorta ..................................................

+53 GT258781_Pcontorta ..................................................

CONSENSUS ATTCATAAGCTTCACGTACCTGTGCAGTAAAGTTTTGATTTTTGGGTTTC

+ Frame 3 I H K L H V P V Q * S F D F W V S

260 270 280 290 300

+80 GT230706_Pcontorta ..................................................

+53 GT258781_Pcontorta ..................................................

CONSENSUS TGATCGGCTGCAATCCATCCTTTGCTTTGCTTGATCTCACCACCGTCCCT

+ Frame 3 D R L Q S I L C F A * S H H R P

310 320 330 340 350

+80 GT230706_Pcontorta ..................................................

+53 GT258781_Pcontorta ..................................................

CONSENSUS CTCTCTCTCTCTGCCTGCTCTCGTGAAACTGTTTAATTTCTGGACCGTTC

+ Frame 3 S L S L C L L S * N C L I S G P F

360 370 380 390 400

+80 GT230706_Pcontorta ..................................................

+53 GT258781_Pcontorta ..................................................

CONSENSUS ATGTCTGTTCACTTTTTCTATATAATTTACTCCACTTACCTCTGAGATTT

+ Frame 3 M S V H F F Y I I Y S T Y L * D F

410 420 430 440 450

+80 GT230706_Pcontorta ..................................................

+53 GT258781_Pcontorta ..................................................

CONSENSUS TGGCAGTCAATGATTTCTGTGCTGGAATTCGTTTTAATTTCAGATTGATT

+ Frame 3 G S Q * F L C W N S F * F Q I D

460 470 480 490 500

+80 GT230706_Pcontorta ..................................................

+53 GT258781_Pcontorta ..................................................

CONSENSUS CCTTCTTTTTCTGATACAAGCACAGTGCTGCTGTATATGCTATACTTCAT

+ Frame 3 S F F F * Y K H S A A V Y A I L H

510 520 530 540 550

+80 GT230706_Pcontorta ..................................................

+53 GT258781_Pcontorta ..................................................

CONSENSUS AGGCCTGTTATTGGTGAAATTGCTGGGACGATGATTGAGAGGAGAAGGCC

+ Frame 3 R P V I G E I A G T M I E R R R P

560 570 580 590 600

+80 GT230706_Pcontorta ..................................................

+53 GT258781_Pcontorta ..................................................

+47 GT259122_Pcontorta ..............................................

CONSENSUS TGCGAAATTGGATAAGAAGATGAATCTTGCCGCAGTGGTTAGTGTATTGG

+ Frame 3 A K L D K K M N L A A V V S V L

610 620 630 640 650

+80 GT230706_Pcontorta ..................................................

+53 GT258781_Pcontorta ..................................................

+47 GT259122_Pcontorta ..................................................

CONSENSUS TGATAATGATCTTGATTATGCTCTCCAGTTTAATATGTTTTGCATCAGCA

+ Frame 3 V I M I L I M L S S L I C F A S A

660 670 680 690 700

+80 GT230706_Pcontorta ........

+53 GT258781_Pcontorta ..................................................

+47 GT259122_Pcontorta ..................................................

+75 GT231018_Pcontorta ................................

CONSENSUS GCAAGGCAGTCTGCCTTTTTCCATGCAGAAATGAAGAATAAAGATCATAA

+ Frame 3 A R Q S A F F H A E M K N K D H K

710 720 730 740 750

+53 GT258781_Pcontorta ..................................................

+47 GT259122_Pcontorta ..................................................

+75 GT231018_Pcontorta ..................................................

CONSENSUS AGCTGCCTCTGGTTTCTTTAAACCCTCTGGCAAGGATTGCAGTTCAGGGA

+ Frame 3 A A S G F F K P S G K D C S S G

760 770 780 790 800

+53 GT258781_Pcontorta ..................................................

+47 GT259122_Pcontorta ..................................................

+75 GT231018_Pcontorta ..................................................

CONSENSUS AGTCTAGTCTCAGCCACTGCAGCCCTCTTCCCAAGCAGATGGGCAATAGG

+ Frame 3 K S S L S H C S P L P K Q M G N R

810 820 830 840 850

+53 GT258781_Pcontorta ..................................................

+47 GT259122_Pcontorta ..................................................

+75 GT231018_Pcontorta ..................................................

CONSENSUS AATACAACTGGAGCAGATAAACGTGTAGTTCCTACTGGCCCAAATCCCTT

+ Frame 3 N T T G A D K R V V P T G P N P L

860 870 880 890 900

+53 GT258781_Pcontorta ..................................................

+47 GT259122_Pcontorta ..................................................

+75 GT231018_Pcontorta ..................................................

CONSENSUS GCACAACAGGTGAAGCAGAAGGATTGTATGATATTATTTCATCATGGAGG

+ Frame 3 H N R * S R R I V * Y Y F I M E

910 920 930 940 950

+53 GT258781_Pcontorta ..................................................

+47 GT259122_Pcontorta ..................................................

+75 GT231018_Pcontorta ..................................................

+78 GT260201_Pcontorta -.........................................

+79 GT260539_Pcontorta -.........................................

CONSENSUS CTGATGATCAGCCTAGGGTTGAGATTTCAGTGCAGGAAAATCCAACTATT

+ Frame 3 A D D Q P R V E I S V Q E N P T I

960 970 980 990 1000

+53 GT258781_Pcontorta ................

+47 GT259122_Pcontorta ..................................................

+75 GT231018_Pcontorta ..................................................

+78 GT260201_Pcontorta ..................................................

+79 GT260539_Pcontorta ..................................................

CONSENSUS ATCTATCTTTCTATATATGTTTATTACTATTAGTATATCTATATACCTAT

+ Frame 3 I Y L S I Y V Y Y Y * Y I Y I P I

1010 1020 1030 1040 1050

+47 GT259122_Pcontorta ..................................................

+75 GT231018_Pcontorta ..................................................

+78 GT260201_Pcontorta ..................................................

+79 GT260539_Pcontorta ..................................................

CONSENSUS ATCTATGCAAAAGCTGAGCTGTGTTAGAGTTTTAGGCTGCCTGAGAAACT

+ Frame 3 S M Q K L S C V R V L G C L R N

1060 1070 1080 1090 1100

+47 GT259122_Pcontorta ..................................................

+75 GT231018_Pcontorta ..................................................

+78 GT260201_Pcontorta ..................................................

+79 GT260539_Pcontorta ..................................................

CONSENSUS GTGTTGAAGATGTTCATGACAGCCATGAACAGGACCAGCAAAAAGAGTAT

+ Frame 3 C V E D V H D S H E Q D Q Q K E Y

1110 1120 1130 1140 1150

+47 GT259122_Pcontorta ..................................................

+75 GT231018_Pcontorta ..................................................

+78 GT260201_Pcontorta ..................................................

+79 GT260539_Pcontorta ..................................................

CONSENSUS TGTAACATAGAGCTGATCATGCTATTTGATCATAGCTGCAATCTTGTGGT

+ Frame 3 C N I E L I M L F D H S C N L V V

1160 1170 1180 1190 1200

+47 GT259122_Pcontorta ..................................................

+75 GT231018_Pcontorta ..................................................

+78 GT260201_Pcontorta ..................................................

+79 GT260539_Pcontorta ..................................................

CONSENSUS GTAGTAATTTGAATTGTGGTAGGTAGAGTATTGGCTCATCGGTTTTTCAT

+ Frame 3 * * F E L W * V E Y W L I G F S

1210 1220 1230 1240 1250

+47 GT259122_Pcontorta ..................................................

+75 GT231018_Pcontorta ..................................................

+78 GT260201_Pcontorta ..................................................

+79 GT260539_Pcontorta ..................................................

CONSENSUS GGACCGAATTTTGTCTCAGTATATATTATTTTTATACATATGTTGATATA

+ Frame 3 W T E F C L S I Y Y F Y T Y V D I

1260 1270 1280 1290 1300

+47 GT259122_Pcontorta ..................................................

+75 GT231018_Pcontorta ..................................................

+78 GT260201_Pcontorta ..................................................

+79 GT260539_Pcontorta ..................................................

CONSENSUS TGCCACTTTTAGCTTCTGGAGCATTGGTTCCATGTCCCAGAAGCTCTAAG

+ Frame 3 C H F * L L E H W F H V P E A L S

1310 1320 1330 1340 1350

+47 GT259122_Pcontorta ..................................................

+75 GT231018_Pcontorta ..................................................

+78 GT260201_Pcontorta ..................................................

+79 GT260539_Pcontorta ..................................................

CONSENSUS TGGGGTGAAATCATTCTATGTTTGCAGCGAACAGCAGCAAAATTTGTTCA

+ Frame 3 G V K S F Y V C S E Q Q Q N L F

1360 1370 1380 1390 1400

+47 GT259122_Pcontorta ..................................................

+75 GT231018_Pcontorta ..................................................

+78 GT260201_Pcontorta ..................................................

+79 GT260539_Pcontorta ..................................................

CONSENSUS TGTCCAATGGCTAATTGGAATCTATTTATACATTTGGTGTCTTTTTACTA

+ Frame 3 M S N G * L E S I Y T F G V F L L

1410 1

+47 GT259122_Pcontorta ................

+75 GT231018_Pcontorta ...............

+78 GT260201_Pcontorta .................

+79 GT260539_Pcontorta .................

CONSENSUS AAAAAAAAAAAAAAAAA

+ Frame 3 K K K K K -

1. ***Pinus contorta CLE202***

10 20 30 40 50

+77 GT250045_Pcontorta ..................................................

+64 GT253464_Pcontorta ...............................................

CONSENSUS CCCGGGGAAGAAAAACGCTACCCATTTCATTTGTGTTGTTATTCCAACCT

+ Frame 1 P G E E K R Y P F H L C C Y S N L

60 70 80 90 100

+77 GT250045_Pcontorta ..................................................

+64 GT253464_Pcontorta ..................................................

CONSENSUS AGAGTCTACACAGGCATTAGTCATTAATTCCAGTTGAATCATGTGGTGGT

+ Frame 1 E S T Q A L V I N S S * I M W W

110 120 130 140 150

+77 GT250045_Pcontorta ..................................................

+64 GT253464_Pcontorta ..................................................

CONSENSUS GGTGTTTTATTATTCTGAGAATGGAGAAATTTAGGGTTGGTTATTATATA

+ Frame 1 W C F I I L R M E K F R V G Y Y I

160 170 180 190 200

+77 GT250045_Pcontorta ..................................................

+64 GT253464_Pcontorta ..................................................

CONSENSUS AGGATTCATGGCAACCATATGATGATGATCATAATGCTGTGTCTTCTGGC

+ Frame 1 R I H G N H M M M I I M L C L L A

210 220 230 240 250

+77 GT250045_Pcontorta ..................................................

+64 GT253464_Pcontorta ..................................................

CONSENSUS AGCTGCTGTTCATTGCAGGGCAGGACGTTCAAGGGTCTTAATTGTGGGAG

+ Frame 1 A A V H C R A G R S R V L I V G

260 270 280 290 300

+77 GT250045_Pcontorta ..................................................

+64 GT253464_Pcontorta ..................................................

CONSENSUS TGGGAGAAACAAATGGGAGACCTCAGCATCTACTTAGCCATCAGCTGGAG

+ Frame 1 V G E T N G R P Q H L L S H Q L E

310 320 330 340 350

+77 GT250045_Pcontorta ..................................................

+64 GT253464_Pcontorta ..................................................

CONSENSUS GAGGATCAGTACGTTGAAGGAGATTATGCTTCAACTTCTTCATCAAATGA

+ Frame 1 E D Q Y V E G D Y A S T S S S N D

360 370 380 390 400

+77 GT250045_Pcontorta ..................................................

+64 GT253464_Pcontorta ..................................................

CONSENSUS TGATCAGTTCAGAAGAAGCAGAGACTGTTGCTACTCTCACTATTGTGGAC

+ Frame 1 D Q F R R S R D C C Y S H Y C G

410 420 430 440 450

+77 GT250045_Pcontorta ..................................................

+64 GT253464_Pcontorta ..................................................

CONSENSUS ATTGCAGCAGGAGGAGCAGGAGCAGGAGCAGCAGGTCTTCTGCAGCAGCA

+ Frame 1 H C S R R S R S R S S R S S A A A

460 470 480 490 500

+77 GT250045_Pcontorta ..................................................

+64 GT253464_Pcontorta ..................................................

CONSENSUS GAAAAAGGATCCTTGGATCCATTGGATCCAGTTAATTATAATGTGGATGA

+ Frame 1 E K G S L D P L D P V N Y N V D E

510 520 530 540 550

+77 GT250045_Pcontorta ..................................................

+64 GT253464_Pcontorta ..................................................

CONSENSUS AAGATTGGTTCCCACAGGCCCTGATCCATTACATAATTGATTATGGAAAA

+ Frame 1 R L V P T G P D P L H N * L W K

560 570 580 590 600

+77 GT250045_Pcontorta ..................................................

+64 GT253464_Pcontorta ..................................................

CONSENSUS TGTTGGATGCATGGTTATTGGAGAGTGTTTGGAGTCTTGTTAAGAACTAA

+ Frame 1 M L D A W L L E S V W S L V K N *

610 620 630 640 650

+77 GT250045_Pcontorta .....................................*............

+64 GT253464_Pcontorta .....................................T............

CONSENSUS GTATTATAGCCCATCATCAGAAGAATCCTGGGCATTT-CCAAGATCTTAT

+ Frame 1 V L * P I I R R I L G I - Q D L I

660 670 680 690 700

+77 GT250045_Pcontorta .....*............................................

+64 GT253464_Pcontorta .....T............................................

CONSENSUS AATAC-TGTGATTTATATGGTTGTGTTTTGTAACTCCACATTTTTTCTTT

+ Frame 1 I - V I Y M V V F C N S T F F L

710 720 730 740 750

+77 GT250045_Pcontorta ..................................................

+64 GT253464_Pcontorta .........

CONSENSUS GGGCCCAGTGGGATTGATCTCCTGGAACGTTTCAGTTTCAGTATAGGGAT

+ Frame 1 W A Q W D * S P G T F Q F Q Y R D

760 770 780 790 800

+77 GT250045_Pcontorta ..................................................

CONSENSUS TGTTAGGAGACATGGTGCCAGTATCGAGATTCGAACCTGAGTCAATGGAG

+ Frame 1 C * E T W C Q Y R D S N L S Q W R

810 820 830 840 850

+77 GT250045_Pcontorta ..................................................

CONSENSUS GTAAACTCCCAAGCACGGCTCAACTCATTGTTATATTGTATTTTTGTGAA

+ Frame 1 * T P K H G S T H C Y I V F L *

860 870 880 8

+77 GT250045_Pcontorta ......................................

CONSENSUS ATGTGTGAGAATTATTGGGTAAAAAAAAAAAAAAAAAA

+ Frame 1 N V * E L L G K K K K K K

1. ***Pinus banksiana CLE203***

10 20 30 40 50

+48 GW740769_Pbanksiana ..................................................

CONSENSUS GGGGGAATATAATTTCATGGTTTTGTTTGTTTTTGTAGAGGGAAGCAGGG

+ Frame 3 G N I I S W F C L F L * R E A G

60 70 80 90 100

+48 GW740769_Pbanksiana ..................................................

CONSENSUS GATTTTGAACTTGATTTTCTTTGGCTTACATAATACTTTATGGGATGGCC

+ Frame 3 D F E L D F L W L T * Y F M G W P

110 120 130 140 150

+48 GW740769_Pbanksiana ..................................................

CONSENSUS AATTGAGCTTTCATGGTTTTGCACAAGGTTGGTTCGAAATAAGAGATTTT

+ Frame 3 I E L S W F C T R L V R N K R F

160 170 180 190 200

+48 GW740769_Pbanksiana ..................................................

CONSENSUS AAATTTGAATTTCTTGGTTTACATGATACTTATAGCATGGCGATCACCAA

+ Frame 3 * I * I S W F T * Y L * H G D H Q

210 220 230 240 250

+48 GW740769_Pbanksiana ..................................................

CONSENSUS CTGACCTGCCCTGATAGACTTGAGAAACTCATAACATTAACAGTGGGGTA

+ Frame 3 L T C P D R L E K L I T L T V G Y

260 270 280 290 300

+48 GW740769_Pbanksiana ..................................................

-54 GW745618_Pbanksiana .....................................

CONSENSUS TTGGTACTCTTTGCTTCAATAAATTGTTGCATTCCAGGCTTTGAGACTTG

+ Frame 3 W Y S L L Q * I V A F Q A L R L

310 320 330 340 350

+48 GW740769_Pbanksiana ..................................................

-54 GW745618_Pbanksiana ..................................................

CONSENSUS GATGGATTTGGGTTTCGCTAGGCATCTTACAGGGGGCAGTCTGTATGGCG

+ Frame 3 G W I W V S L G I L Q G A V C M A

360 370 380 390 400

+48 GW740769_Pbanksiana ..................................................

-54 GW745618_Pbanksiana ..................................................

CONSENSUS GATGGTTTTGTTAGAAGTCTTAAAAGGGCAGATAAAAAGTTGCTGCTTAT

+ Frame 3 D G F V R S L K R A D K K L L L I

410 420 430 440 450

+48 GW740769_Pbanksiana ..................................................

-54 GW745618_Pbanksiana ..................................................

CONSENSUS AATTTTTCTGTTGTTGGGTCTCTTCTTTCAGCAAGTAGATTCAACACCAT

+ Frame 3 I F L L L G L F F Q Q V D S T P

460 470 480 490 500

+48 GW740769_Pbanksiana ..................................................

-54 GW745618_Pbanksiana ..................................................

CONSENSUS GCCCAGGAAAAAAGTTGCAGGATTTTGGTCATGGAGAATTAGGGGTTACA

+ Frame 3 C P G K K L Q D F G H G E L G V T

510 520 530 540 550

+48 GW740769_Pbanksiana ..................................................

-54 GW745618_Pbanksiana ..................................................

CONSENSUS TTGAAGCATTCTCCATGTCGAAGGATCTTGGCTTCCAGGGAATTCTTTGT

+ Frame 3 L K H S P C R R I L A S R E F F V

560 570 580 590 600

+48 GW740769_Pbanksiana ..................................................

-54 GW745618_Pbanksiana ..................................................

CONSENSUS TCCCAAGCACTCTAAGGGCTCCAGAACACGTTATGAGGAAAGCGATCATG

+ Frame 3 P K H S K G S R T R Y E E S D H

610 620 630 640 650

+48 GW740769_Pbanksiana ..................................................

-54 GW745618_Pbanksiana ..................................................

CONSENSUS AAGTTCCCAGCGGTCCAAATCCAATTAGTAATAGATCGGATCAGCTTTCT

+ Frame 3 E V P S G P N P I S N R S D Q L S

660 670 680 690 700

+48 GW740769_Pbanksiana ..................................................

-54 GW745618_Pbanksiana ..................................................

CONSENSUS CTGGTGTGTTTTGATAAAGAGCTCAGAATATCGGGGTTCGAAGCTTCATC

+ Frame 3 L V C F D K E L R I S G F E A S S

710 720 730 740 750

+48 GW740769_Pbanksiana .G..................................

-54 GW745618_Pbanksiana .A................................................

CONSENSUS GRATACAGTAAACTAATATTTCTCAGTGTCCATGCCAGAGCTCTTTCGGC

+ Frame 3 D/N T V N * Y F S V S M P E L F R

760 770 780 790 800

-54 GW745618_Pbanksiana ..................................................

CONSENSUS ACTTGCAATACGGTATAATATCAGATAAAGTTATAGGGAAATATTTTAAA

+ Frame 3 H L Q Y G I I S D K V I G K Y F K

810 820 830 840 850

-54 GW745618_Pbanksiana ..................................................

CONSENSUS GAGATAGCCAGTACTTCTCTTTAAATTCACCATTGCCAAAAGATCACACT

+ Frame 3 E I A S T S L * I H H C Q K I T L

860 870 880 890 900

-54 GW745618_Pbanksiana ..................................................

CONSENSUS TTTCTTTTTATCAAGCATCTGTATTCTTATGTGGTCTGCTGTAAGAAAAA

+ Frame 3 F F L S S I C I L M W S A V R K

910 920 930 940 950

-54 GW745618_Pbanksiana ..................................................

CONSENSUS GGAAAAGTAGTCAGGGAGGATTGAGAAAAGGGTATAAGAATATAGAACTG

+ Frame 3 R K S S Q G G L R K G Y K N I E L

960 970 980 990 1000

-54 GW745618_Pbanksiana ..................................................

CONSENSUS CGCTTGCTGCAGCTATAAGAACTTTTGATATGTATTTTTTCTTTATTATG

+ Frame 3 R L L Q L * E L L I C I F S L L *

1010 1020 1030 1040 1050

-54 GW745618_Pbanksiana ..................................................

CONSENSUS AGTGGCCAAATTTTAGACTGATTTTATTAAGTTTCTATAGCACTATAAAT

+ Frame 3 V A K F * T D F I K F L * H Y K

1060 1070

-54 GW745618_Pbanksiana ......................

CONSENSUS TGATGGAAAAAAAAAAAAAAAA

+ Frame 3 L M E K K K K -

1. ***Pinus banksiana CLE204***

10 20 30 40 50

-39 GW772842_Pbanksiana ..................................................

CONSENSUS ATACTGATAAGAAATTTGATCTGTGCTAGTTATATCTTTGCTAGACAGAT

+ Frame 1 I L I R N L I C A S Y I F A R Q I

60 70 80 90 100

-39 GW772842_Pbanksiana ..................................................

CONSENSUS AAGGGATTTGATATCTTGCCTGTACATAATAGATATTAGAATCTCCATGA

+ Frame 1 R D L I S C L Y I I D I R I S M

110 120 130 140 150

-39 GW772842_Pbanksiana ..................................................

CONSENSUS AAGTTGAGAGGATGGGAATATTGGGTCTCTGTGTCTCTGCCTTGATATTA

+ Frame 1 K V E R M G I L G L C V S A L I L

160 170 180 190 200

-39 GW772842_Pbanksiana ..................................................

CONSENSUS ATTCTTCTGGTCTTGTCCAGTATATGTGGGGCTGAATCCCGGAGAATGCT

+ Frame 1 I L L V L S S I C G A E S R R M L

210 220 230 240 250

-39 GW772842_Pbanksiana ..................................................

CONSENSUS CACAGACAGCAATGGCCGAAGATATCTTGAGAAGAATTCTGATGACCCAG

+ Frame 1 T D S N G R R Y L E K N S D D P

260 270 280 290 300

-39 GW772842_Pbanksiana ..................................................

CONSENSUS GGAACCAAGCCCTCAATATTCCAGCCCCTCGGACCCCTGTCTATGAATCA

+ Frame 1 G N Q A L N I P A P R T P V Y E S

310 320 330 340 350

-39 GW772842_Pbanksiana ..................................................

CONSENSUS GATGATGGTAATGAGATTGATCCCAGGTATGGAGTGGAGAAGAGATTGGT

+ Frame 1 D D G N E I D P R Y G V E K R L V

360 370 380 390 400

-39 GW772842_Pbanksiana ..................................................

CONSENSUS TCCCACAGGTCCAAATCCTCTTCACCATTGAACATGGCCATGGAGTCATG

+ Frame 1 P T G P N P L H H * T W P W S H

410 420 430 440 450

-39 GW772842_Pbanksiana ..................................................

CONSENSUS GAGGTCCAAAGCTCAAGGCCAGGACTGTACATTAGTTCAGATCAATCTTC

+ Frame 1 G G P K L K A R T V H * F R S I F

460 470 480 490 500

-39 GW772842_Pbanksiana ..................................................

CONSENSUS CAAGATCTGGGCTTCTATATGGATTCAAGAGTATTGGTTCCTTAACATGG

+ Frame 1 Q D L G F Y M D S R V L V P * H G

510 520 530 540 550

-39 GW772842_Pbanksiana ..................................................

CONSENSUS GTTTACTGGTCATGATCAATATAGAGCTGTTTGTTACAGGAAGAATTTGT

+ Frame 1 F T G H D Q Y R A V C Y R K N L

560 570 580 590 600

-39 GW772842_Pbanksiana ..................................................

CONSENSUS ATGTTTTTAAGGCATCCAGGCACTTTGATGGCAACTATTGAATTTCAGGC

+ Frame 1 Y V F K A S R H F D G N Y * I S G

610 620 630 640 650

-39 GW772842_Pbanksiana ..................................................

CONSENSUS TCAGAAGCTTGAAGGCTAGAAGCTTTTGACACTTCAGTGGCTGCTGGAGT

+ Frame 1 S E A * R L E A F D T S V A A G V

660 670 680 690 700

-39 GW772842_Pbanksiana ..................................................

CONSENSUS CCATGGCTGCTATAACCATATATTGGGTTGTTTATAATTGCCTTGAGATC

+ Frame 1 H G C Y N H I L G C L * L P * D

710 720 730 740 750

-39 GW772842_Pbanksiana ..................................................

CONSENSUS ATTCTCAAGCCCTGTAGTTTTGGTTGTTATGCATTAATGCTTCCTCAGAG

+ Frame 1 H S Q A L * F W L L C I N A S S E

760 770

-39 GW772842_Pbanksiana .........................

CONSENSUS TTCAATTACAAAAAAAAAAAAAAAA

+ Frame 1 F N Y K K K K K

1. ***Picea sitchensis CLE205***

10 20 30 40 50

+69 GH287489_Psitchensis ..................................................

+70 GH287857_Psitchensis ..................................

CONSENSUS GGGGGTCTGCTCGGAAGGCAGAAGGTTCAGATGGCCAAAAACCATTTTAT

+ Frame 1 G G L L G R Q K V Q M A K N H F M

60 70 80 90 100

+69 GH287489_Psitchensis G.................................................

+70 GH287857_Psitchensis -.................................................

CONSENSUS GTTTCCAAAGCTCAATGCTAGTTATGATGTAGTCATGATCTTCTTGCTTG

+ Frame 1 F P K L N A S Y D V V M I F L L

110 120 130 140 150

+69 GH287489_Psitchensis ..................................................

+70 GH287857_Psitchensis ..................................................

CONSENSUS TAGTTTCTTCTCAGTTGATCTCTGCAGCTCTGGGCATTAGAAACTTCAAT

+ Frame 1 V V S S Q L I S A A L G I R N F N

160 170 180 190 200

+69 GH287489_Psitchensis ..................................................

+70 GH287857_Psitchensis ..................................................

CONSENSUS TCTTCAGACAATATGCAGAAACAGCGACTGCTGGATGGCTTATCAGCAGC

+ Frame 1 S S D N M Q K Q R L L D G L S A A

210 220 230 240 250

+69 GH287489_Psitchensis ..................................................

+70 GH287857_Psitchensis ..................................................

CONSENSUS TACAGTCATGTACTCAGCTAATAAAAATGGGCAGCCTGATGGATTCAAAG

+ Frame 1 T V M Y S A N K N G Q P D G F K

260 270 280 290 300

+69 GH287489_Psitchensis ..................................................

+70 GH287857_Psitchensis ..................................................

CONSENSUS CTGATGTTACTGCAACTAATCTGGATCCAAATTTCACCAGCAAGCGCATG

+ Frame 1 A D V T A T N L D P N F T S K R M

310 320 330 340 350

+69 GH287489_Psitchensis ..................................................

+70 GH287857_Psitchensis ..................................................

CONSENSUS GTTCCTAATGGGTCCGACCCTCTTCATAATCGGTGATCTTACATGTATGT

+ Frame 1 V P N G S D P L H N R * S Y M Y V

360 370 380 390 400

+69 GH287489_Psitchensis ..................................................

+70 GH287857_Psitchensis ..................................................

CONSENSUS ACAGATCGATTCCAAGAGTACTAGTTTAGGGAATATCAATGGCTCCGACC

+ Frame 1 Q I D S K S T S L G N I N G S D

410 420 430 440 450

+69 GH287489_Psitchensis ..................................................

+70 GH287857_Psitchensis ..................................................

CONSENSUS CTCTTAATAATCGGTGATCTTACATATATGTACAGATCGATTCCAAGAGT

+ Frame 1 P L N N R * S Y I Y V Q I D S K S

460 470 480 490 500

+69 GH287489_Psitchensis ..................................................

+70 GH287857_Psitchensis ..................................................

CONSENSUS ACTAGTTTAGGGAAAGGGAATATCAATGTAAGCAGAGGATGAAGGAGAAG

+ Frame 1 T S L G K G N I N V S R G * R R R

510 520 530 540 550

+69 GH287489_Psitchensis ..................................................

+70 GH287857_Psitchensis ..................................................

CONSENSUS ATTAGAATTCTGTCACTGAAGCTATTCCAAGACATCATAATATACAGTAC

+ Frame 1 L E F C H * S Y S K T S * Y T V

560 570 580 590 600

+69 GH287489_Psitchensis ..................................................

+70 GH287857_Psitchensis ..................................................

CONSENSUS TAGTCCAGTGAATATCAATACAAGCAGAAGAGGAAAAATAAGATTATAAA

+ Frame 1 L V Q * I S I Q A E E E K * D Y K

610 620 630 640 650

+69 GH287489_Psitchensis ..................................................

+70 GH287857_Psitchensis ..................................................

CONSENSUS GTATTAATCCAGCAGCAAATATCAATGTAAGCAGAAGAGGAGGAAGAAGA

+ Frame 1 V L I Q Q Q I S M * A E E E E E E

660 670 680 690 700

+69 GH287489_Psitchensis ..................................................

+70 GH287857_Psitchensis ..................................................

CONSENSUS AAAGAATTATTCACACAGGCCACATAGAAGAAGAGGAGGAGGAGGAGATC

+ Frame 1 K N Y S H R P H R R R G G G G D

710 720 730 740 750

+69 GH287489_Psitchensis ..................................................

+70 GH287857_Psitchensis ..................................................

CONSENSUS GCCCAGCAAGAAAACATGTAGATATGGATATGAGTAAAAGTGATGTGCTC

+ Frame 1 R P A R K H V D M D M S K S D V L

760 770 780 790 800

+69 GH287489_Psitchensis ..................................................

+70 GH287857_Psitchensis ..................................................

CONSENSUS AATGCTAGAATTAAGAAACTCATACAGAGTTCTTTATGTTTTGGG**TTG

+ Frame 1 N A R I K K L I Q S S L C F G L

810 820 830 840 850

+69 GH287489_Psitchensis ..........................................

+70 GH287857_Psitchensis ..................................................

CONSENSUS GTTTTTCCAATCAGAAGAGATACTGAGCTTCAGATTTCACCTAAAAAAAA

+ Frame 1 V F P I R R D T E L Q I S P K K K

860

+70 GH287857_Psitchensis ..........

CONSENSUS AAAAAAAAAA

+ Frame 1 K K K

1. ***Picea sitchensis CLE206***

10 20 30 40 50

+81 GH290648_Psitchensis ..................................................

CONSENSUS GGGGGGCAGGCTTATGAGTGGATTCCTGTGGTCAGAGTAAGAATGTAGGG

+ Frame 1 G G Q A Y E W I P V V R V R M * G

60 70 80 90 100

+81 GH290648_Psitchensis ..................................................

CONSENSUS CTTGATATCTGATAGAGACTCGAATTCGGCCTGTTTTTTCTTTACCCTGT

+ Frame 1 L I S D R D S N S A C F F F T L

110 120 130 140 150

+81 GH290648_Psitchensis ..................................................

CONSENSUS ATTTATTGGTATTCAGTGAAGAAATGATGATGCATTAAATTCATGTAAGT

+ Frame 1 Y L L V F S E E M M M H * I H V S

160 170 180 190 200

+81 GH290648_Psitchensis ..................................................

CONSENSUS GGTATATGGTAAGGAGTTCAGGTACTTTGTGAGTCGAAGATTCATCAGGG

+ Frame 1 G I W * G V Q V L C E S K I H Q G

210 220 230 240 250

+81 GH290648_Psitchensis ..................................................

CONSENSUS AGAAGATACGGGGGATTAATTTCAAAGCAGAGGTTGAGAAGAAGCAGTGG

+ Frame 1 E D T G D * F Q S R G * E E A V

260 270 280 290 300

+81 GH290648_Psitchensis ..................................................

CONSENSUS TGAAGGATTTAATGGAAGAGCATGAAGCCCCGTGAGCTTTGAAGGCTGTT

+ Frame 1 V K D L M E E H E A P * A L K A V

310 320 330 340 350

+81 GH290648_Psitchensis ..................................................

CONSENSUS GATTGATCTTTTCCCTTTGCATTAACGACATTCATAAGCTTCACGTACCT

+ Frame 1 D * S F P F A L T T F I S F T Y L

360 370 380 390 400

+81 GH290648_Psitchensis ..................................................

CONSENSUS GTGCAGTAAAGTTTTTATTTTTGGGTTCCTCCTTATCTTAAAGATCGGCT

+ Frame 1 C S K V F I F G F L L I L K I G

410 420 430 440 450

+81 GH290648_Psitchensis ..................................................

CONSENSUS GCAATCCATCCTTGCTTTGCTTCATCTCACCACCGTCCATCTCTCTGTCT

+ Frame 1 C N P S L L C F I S P P S I S L S

460 470 480 490 500

+81 GH290648_Psitchensis ..................................................

CONSENSUS CTGCTCTCGTGAAACTGTTTAATTTCTAGACTGTTCAGCTTTCTATATAA

+ Frame 1 L L S * N C L I S R L F S F L Y N

510 520 530 540 550

+81 GH290648_Psitchensis ..................................................

CONSENSUS TTTACTGCACTCGCCTCTGAGATTTTGGCTGTCCGGGATTTCTGTGCTGG

+ Frame 1 L L H S P L R F W L S G I S V L

560 570 580 590 600

+81 GH290648_Psitchensis ..................................................

CONSENSUS AATTCGTTTTATATTCAGATCGATTCCTTCTTTTGTTTGGTTTTCTGATA

+ Frame 1 E F V L Y S D R F L L L F G F L I

610 620 630 640 650

+81 GH290648_Psitchensis ..................................................

CONSENSUS TAGCACAGTACTGTATATGATATATTTTTTAGACCTATTATCGGTGAAAT

+ Frame 1 * H S T V Y D I F F R P I I G E I

660 670 680 690 700

+81 GH290648_Psitchensis ..................................................

+71 GH291026_Psitchensis ........

CONSENSUS TGCTGGGACGATGATTGAGAGGAGAAGGCCTGAGAAATTGAATAGGATGA

+ Frame 1 A G T M I E R R R P E K L N R M

710 720 730 740 750

+81 GH290648_Psitchensis ..................................................

+71 GH291026_Psitchensis ..................................................

CONSENSUS TGAATCTTGCTGCGGTGGTTAGCGTATTGGTAGTAATGATCCTGATTATA

+ Frame 1 M N L A A V V S V L V V M I L I I

760 770 780 790 800

+81 GH290648_Psitchensis ..................................................

+71 GH291026_Psitchensis ..................................................

CONSENSUS CTCTCCAGTTTAATATGTTTTGCATCTGCAGCAAGGCAGTCCGCGTTTTT

+ Frame 1 L S S L I C F A S A A R Q S A F F

810 820 830 840 850

+81 GH290648_Psitchensis ..................................................

+71 GH291026_Psitchensis ..................................................

CONSENSUS CCATGCAGAGATGAAGGATAAAGATCATAAAGCTGCCTCCGGTTTGTTTA

+ Frame 1 H A E M K D K D H K A A S G L F

860 870 880 890 900

+81 GH290648_Psitchensis .........................

+71 GH291026_Psitchensis ..................................................

CONSENSUS AACCCTCTGGCAAGGATTGCCATTCAGGGAAATCTCTCAGCCACTGCAGC

+ Frame 1 K P S G K D C H S G K S L S H C S

910 920 930 940 950

+71 GH291026_Psitchensis ..................................................

CONSENSUS CCTATTTCGAAGCAGATGGGCAATAGCAATATGACTGGAGCAGATAAACG

+ Frame 1 P I S K Q M G N S N M T G A D K R

960 970 980 990 1000

+71 GH291026_Psitchensis ..................................................

CONSENSUS CGTAGTGCCCACTGGCCCAAATCCCTTGCACAACAGGTGAACCATATCAG

+ Frame 1 V V P T G P N P L H N R * T I S

1010 1020 1030 1040 1050

+71 GH291026_Psitchensis ..................................................

CONSENSUS TCCAAGGATTGTATGATATTAATCATCATGGAGGCTGATCAACCTAGGGT

+ Frame 1 V Q G L Y D I N H H G G * S T * G

1060 1070 1080 1090 1100

+71 GH291026_Psitchensis ..................................................

CONSENSUS TGGGATTTCAGTGCAGGAAAAACCAACTATATCTAT*CTATCTATCTATC

+ Frame 1 W D F S A G K T N Y I Y L S I Y

1110 1120 1130 1140 1150

+71 GH291026_Psitchensis ..................................................

CONSENSUS TATATCTATTACTATTACTATATCTTATCTATGCAAAAACTGAGCTGTGT

+ Frame 1 L Y L L L L L Y L I Y A K T E L C

1160 1170 1180 1190 1200

+71 GH291026_Psitchensis ..................................................

CONSENSUS TAATGTTTTAGGCTGCCTGTGAAACTCTGTTGAAGATGTTCATGAGAGCC

+ Frame 1 * C F R L P V K L C * R C S * E P

1210 1220 1230 1240 1250

+71 GH291026_Psitchensis ..................................................

CONSENSUS ATGAACAGGACCAGCAAAAGGAGTATTGTAACATAGAGACGATGATGCTA

+ Frame 1 * T G P A K G V L * H R D D D A

1260 1270 1280 1290 1300

+71 GH291026_Psitchensis ..................................................

CONSENSUS TTTGATCATAGCTGCAATCTTCTGGTGTATTAAGTTAAATTGTGGTAGGT

+ Frame 1 I * S * L Q S S G V L S * I V V G

1310 1320 1330 1340 1350

+71 GH291026_Psitchensis ..................................................

CONSENSUS AGAGTATTGGCTCAACGGTTTTCCATGTTGGACCGAATTTTGTCGCAGTA

+ Frame 1 R V L A Q R F S M L D R I L S Q Y

1360 1370 1380 1390 1400

+71 GH291026_Psitchensis ..................................................

CONSENSUS TATATTATTTTATACATGTGTTGATATATGCCACTTTTAGCTTCTGGAGC

+ Frame 1 I L F Y T C V D I C H F * L L E

1410 1420 1430 1440 1450

+71 GH291026_Psitchensis ..................................................

CONSENSUS ATTGGTTCCATGTCCCAGAAGCTAAAAGTGGGGTAAAATCATTCTATGTT

+ Frame 1 H W F H V P E A K S G V K S F Y V

1460 1470 1480 1490 1500

+71 GH291026_Psitchensis ..................................................

CONSENSUS CGCAGCGAACAGCAGCAAATTTGTTCTTGTCCAATGGCTAATTGGAATCT

+ Frame 1 R S E Q Q Q I C S C P M A N W N L

1510 1520

+71 GH291026_Psitchensis .........................

CONSENSUS ATTTTATAAAAAAAAAAAAAAAAAA

+ Frame 1 F Y K K K K K K

1. ***Picea sitchensis CLE207***

10 20 30 40 50

+74 GT121713_Psitchensis ..................................................

CONSENSUS TCCCACTGAACAGGTAGATGCAGGCAGATTTGGAGTGGATAAACGACGAG

No predicted signal peptide. Sequence likely not full-length

+ Frame 2 P T E Q V D A G R F G V D K R R

60 70 80 90 100

+74 GT121713_Psitchensis ..................................................

CONSENSUS TCCCCACGGGCTCCAACCCCTTGCACAACAGGTAGCTGGGACGCCCTGAT

+ Frame 2 V P T G S N P L H N R * L G R P D

110 120 130 140 150

+74 GT121713_Psitchensis ..................................................

CONSENSUS TGCCATTAATATGCCTTCTTGTGCCTGCAACTTCAATCGCAAACTTTCAC

+ Frame 2 C H * Y A F L C L Q L Q S Q T F T

160 170 180 190 200

+74 GT121713_Psitchensis ..................................................

CONSENSUS GGCAAGAGAATTATAGCTGTTTTTAATTCCTGATCTGCTAAATTAATACT

+ Frame 2 A R E L * L F L I P D L L N * Y

210 220 230 240 250

+74 GT121713_Psitchensis ..................................................

CONSENSUS TGGATATAGAAAACTTAGGAAGAAATTAGAGTTGAATGAGAAGAAATTGC

+ Frame 2 L D I E N L G R N * S * M R R N C

260 270 280 290 300

+74 GT121713_Psitchensis ..................................................

CONSENSUS GCACAGTCGGCAATAGAAATGTAACATAGAGAGGATGTAATACATGGCTT

+ Frame 2 A Q S A I E M * H R E D V I H G L

310 320 330 340 350

+74 GT121713_Psitchensis ..................................................

CONSENSUS GAAGAAAGTCCTGCTTGCGTTGACTCCACAACGTAACGCTGTCCGGCAGA

+ Frame 2 K K V L L A L T P Q R N A V R Q

360 370 380 390 400

+74 GT121713_Psitchensis ..................................................

CONSENSUS TCCATGTTATTTGCAGCATCGTTTTTCATATTTATGGGCTTTGCACATCC

+ Frame 2 I H V I C S I V F H I Y G L C T S

410 420 430 440 450

+74 GT121713_Psitchensis ..................................................

CONSENSUS CATGCGCACCAAGGTGCAAGGTAAGGCTTTCGTTTTTAGACTGTGCTTTC

+ Frame 2 H A H Q G A R * G F R F * T V L S

460 470 480 490 500

+74 GT121713_Psitchensis ..................................................

CONSENSUS AAGTTTTCGTAATCAGATTCCTGTTAGCTGCAGCAGGCCATCACGACACA

+ Frame 2 S F R N Q I P V S C S R P S R H

510 520 530 540 550

+74 GT121713_Psitchensis ..................................................

CONSENSUS ATAATAACAACCTCGCGGTAACTGGGTAGTGGAATTTGCTGATCTCTGAT

+ Frame 2 N N N N L A V T G * W N L L I S D

560 570 580 590 600

+74 GT121713_Psitchensis ..................................................

CONSENSUS TATGAAGCCCCAGATCCGACGACAGTATATATG*CTTCATATATTATGTA

+ Frame 2 Y E A P D P T T V Y M L H I L C

610 620 630 640 650

+74 GT121713_Psitchensis ..................................................

CONSENSUS TATCGGGATTCCGAAACGACATTTCGAGGAGATCAAATCGTGCTTTCTGA

+ Frame 2 I S G F R N D I S R R S N R A F *

660 670 680 690 700

+74 GT121713_Psitchensis ..................................................

CONSENSUS ACACTTCTGCATATTTATTGGATGTATCAGCTGTAATAATGATTCTTTCA

+ Frame 2 T L L H I Y W M Y Q L * * * F F Q

710 720 730 740 750

+74 GT121713_Psitchensis ..................................................

CONSENSUS AATATATTCATGTTTCAAATAAATCTTAAAACCATGTCTCTGTTCTTCAC

+ Frame 2 I Y S C F K * I L K P C L C S S

760 770 780 790 800

+74 GT121713_Psitchensis ..................................................

CONSENSUS TTTGTTGTGAATATTGTAAATGCTGTCTGAGAGAGAATTCTGTATTATTG

+ Frame 2 L C C E Y C K C C L R E N S V L L

810 820 830 840 850

+74 GT121713_Psitchensis ..................................................

CONSENSUS AACACAAATTAGCGAATTTGAGGTGCCATATTGTGTTTTATAAATTGCAA

+ Frame 2 N T N * R I * G A I L C F I N C K

860 870 880

+74 GT121713_Psitchensis ...............................

CONSENSUS ATCCGTTCAAGCTAAAAAAAAAAAAAAAAAA

+ Frame 2 S V Q A K K K K K K

1. ***Picea engelmannii × glauca CLE208***

10 20 30 40 50

-30 DR470910_PengelxPgla ..................................................

+27 DR450120-1_PengelxPg ..................................................

CONSENSUS GATTTTGGTCCAATCTTATGCAAGCTATGCACAATGTTACTTACTTCCAG

+ Frame 1 D F G P I L C K L C T M L L T S S

60 70 80 90 100

-30 DR470910_PengelxPgla ..................................................

+27 DR450120-1_PengelxPg ..................................................

CONSENSUS CCCCTCGTTTATGCTTCCTCGTGTGATAATTGTTCTTCAAGTCCTCTTGA

+ Frame 1 P S F M L P R V I I V L Q V L L

110 120 130 140 150

-30 DR470910_PengelxPgla ..................................................

+27 DR450120-1_PengelxPg ..................................................

CONSENSUS TCGTTACTGTTGTATGTGCCCATGAAATATCAGGATCGGTATTGGGTAAA

+ Frame 1 I V T V V C A H E I S G S V L G K

160 170 180 190 200

-30 DR470910_PengelxPgla ..................................................

+27 DR450120-1_PengelxPg ..................................................

CONSENSUS GGAGGAAGGAATTCACTTCGTGCAAGATCGAGCTCTCAGGAATTTATAGC

+ Frame 1 G G R N S L R A R S S S Q E F I A

210 220 230 240 250

-30 DR470910_PengelxPgla ..................................................

+27 DR450120-1_PengelxPg ..................................................

CONSENSUS TCCTGTCAATGATAACAACACATACAGAACTCTGCACCGGAAAATTACAA

+ Frame 1 P V N D N N T Y R T L H R K I T

260 270 280 290 300

-30 DR470910_PengelxPgla ..................................................

+27 DR450120-1_PengelxPg ..................................................

CONSENSUS ATGTGATGAGAGTAAGGAAAGTGGATTTTGATGTCAAAGATGAGCGCCAG

+ Frame 1 N V M R V R K V D F D V K D E R Q

310 320 330 340 350

-30 DR470910_PengelxPgla ..................................................

+27 DR450120-1_PengelxPg ..................................................

CONSENSUS GGAGGATTCCAAAACCCTAGAGAAAATAATAAGGGCAATACTGATCGACC

+ Frame 1 G G F Q N P R E N N K G N T D R P

360 370 380 390 400

-30 DR470910_PengelxPgla ..................................................

+27 DR450120-1_PengelxPg ..................................................

CONSENSUS ATACGTCCAAAAGCTGCACAACGTCCCATCTGGGCCGAATCCGATAGGCA

+ Frame 1 Y V Q K L H N V P S G P N P I G

410 420 430 440 450

-30 DR470910_PengelxPgla ..................................................

+27 DR450120-1_PengelxPg ..................................................

CONSENSUS ACTTTGATCCACCAGCACAGATTGACGGCCTCAGAGCTCCTTCTAGGCAT

+ Frame 1 N F D P P A Q I D G L R A P S R H

460 470 480 490 500

-30 DR470910_PengelxPgla ..................................................

+27 DR450120-1_PengelxPg ..................................................

CONSENSUS TAATCTCAGGAGGTACAGTGTTCAAATGTACAGCAACGTCTCTTGGGAAT

+ Frame 1 * S Q E V Q C S N V Q Q R L L G I

510 520 530

-30 DR470910_PengelxPgla .....................................

+27 DR450120-1_PengelxPg .....................................

CONSENSUS CGAATCGGATTTTACACACAAAAAAAAAAAAAAAAAA

+ Frame 1 E S D F T H K K K K K K
