# Supplementary material for: Bioinformatic and phylogenetic analysis of the CLAVATA3/EMBRYO-SURROUNDING REGION (CLE) and the CLE-LIKE signal peptide genes in the Pinophyta
Source: BMC Plant Biol. 2014 Feb 14;14:47. doi: 10.1186/1471-2229-14-47 (PMC4016512; doi:10.1186/1471-2229-14-47)
Supplement: Additional file 2: Figure S2 — Contig analysis of putative conifer CLEL gene ESTs. Putative CLEL gene ESTs were identified, and contig alignments and assignments were performed as described in Methods. Putative signal peptide analysis was conducted as described in Figure S1 Predicted open reading frames are highlighted in turquoise, except for the putative CLE peptide sequences, which are highlighted in yellow. Putative signal peptide cleavage sites are denoted by arrowheads. Potential in-frame ribosome initiation codons consistent with a signal peptide are highlighted in teal. [file 1471-2229-14-47-S2.docx]

**Fig. S2. Contig analysis of putative conifer *CLEL* gene ESTs.** Putative CLEL gene ESTs were identified, and contig alignments and assignments were performed as described in *Methods*. Putative signal peptide analysis was conducted as described in Fig. S1 Predicted open reading frames are highlighted in *turquoise*, except for the putative CLE peptide sequences, which are highlighted in *yellow*. Putative signal peptide cleavage sites are denoted by *arrowheads*. Potential in-frame ribosome initiation codons consistent with a signal peptide are highlighted in *teal*.

1. ***Picea engelmannii × glauca CLEL13***

10 20 30 40 50

-1 CO211752.1_PengelXgl ..................................................

CONSENSUS GTTACCACTGGAGTATCCAGTTGCTTCTAGAGTTGATGAATTGCTCGGGC

+ Frame 2 L P L E Y P V A S R V D E L L G

60 70 80 90 100

-1 CO211752.1_PengelXgl ..................................................

CONSENSUS AATCCTCTGGAGAGAATAATGAAATAGTGAGCAGGAAATTACTGAGAGGA

+ Frame 2 Q S S G E N N E I V S R K L L R G

110 120 130 140 150

-1 CO211752.1_PengelXgl ..................................................

CONSENSUS TCAGTGGGGAGAGGATTGACAGAGTCGGAGAAAGCGGAGCATAGCAGATT

+ Frame 2 S V G R G L T E S E K A E H S R L

160 170 180 190 200

-1 CO211752.1_PengelXgl ..................................................

CONSENSUS GCAAGCAAAACGTAGTGAATCAGATGACAGTCTGCATGTGCCTGACAGAG

+ Frame 2 Q A K R S E S D D S L H V P D R

210 220 230 240 250

-1 CO211752.1_PengelXgl ..................................................

CONSENSUS ATTTGGCCTCTAAGAATAGAGCAGGGATTGCAAATGATCATCATGAGTTG

+ Frame 2 D L A S K N R A G I A N D H H E L

260 270 280 290 300

-1 CO211752.1_PengelXgl ..................................................

CONSENSUS TTGTTGTCAGGTGCCAGCTTGAACCATATATCTACACAAAATGAACGGAA

+ Frame 2 L L S G A S L N H I S T Q N E R K

310 320 330 340 350

-1 CO211752.1_PengelXgl ..................................................

CONSENSUS AAGCATGACTGCAAATGAGCCGAATAACTTACTTGCAGCAAGTATGCAAG

+ Frame 2 S M T A N E P N N L L A A S M Q

360 370 380 390 400

-1 CO211752.1_PengelXgl ..................................................

CONSENSUS TAACACCTCGTCAGATATACGAGAAACCTAGTTTTCACGTTGATTACAGT

+ Frame 2 V T P R Q I Y E K P S F H V D Y S

410 420 430 440 450

-1 CO211752.1_PengelXgl ..................................................

CONSENSUS GGACCTAAAACTCACAACCCTAAGCACCACTGAGCCTTCAGGTCCAAGTC

+ Frame 2 G P K T H N P K H H * A F R S K S

460 470 480 490 500

-1 CO211752.1_PengelXgl ..................................................

CONSENSUS CAGGTCCAGTACGGAATGGAAGATTAGTTGTCCATCACTACCTACTTACT

+ Frame 2 R S S T E W K I S C P S L P T Y

510 520 530 540 550

-1 CO211752.1_PengelXgl ..................................................

CONSENSUS GTTACAGATGGTCAGGGACGAGATGGGAGAAATATATTTATATACATAAA

+ Frame 2 C Y R W S G T R W E K Y I Y I H K

560 570 580 590 600

-1 CO211752.1_PengelXgl ..................................................

CONSENSUS ATGACTACTTATGAGAAGTATATATCTGTAAATGCTAGCTAGAGCTAAGC

+ Frame 2 M T T Y E K Y I S V N A S * S * A

610 620 630 640 650

-1 CO211752.1_PengelXgl ..................................................

CONSENSUS AAGAAGGAATCGGGAGTTTTCCTGTACACATTTTTAAGAGAAAAATTTTA

+ Frame 2 R R N R E F S C T H F * E K N F

660 670 680 690 700

-1 CO211752.1_PengelXgl ..................................................

CONSENSUS TTGAATGCATTTACATATAATTGTTGCCCCATTCGGGAGGGCAAAATGTA

+ Frame 2 I E C I Y I * L L P H S G G Q N V

710 720 730 740 750

-1 CO211752.1_PengelXgl ..................................................

CONSENSUS AGAACAACGGTGATAGTTGTAATATTAGATTCGTTATTGATATCATAACA

+ Frame 2 R T T V I V V I L D S L L I S * H

760 770 780 790 800

-1 CO211752.1_PengelXgl ..................................................

CONSENSUS CTAACTCAATATGCTATGAGTTGAGAAAACTACACACTATAATCAATATT

+ Frame 2 * L N M L * V E K T T H Y N Q Y

810 820 830 840

-1 CO211752.1_PengelXgl ..............................................

CONSENSUS TTTCGTTTAAAAAAAAAAAAAAAAAAAGAAAAAAAAAAAAAAAAAA

+ Frame 2 F S F K K K K K K R K K K K K -

1. ***Picea glauca CLEL14***

10 20 30 40 50

+2 CO477062.2_Pglauca .....................T.......................A....

+9 GE477216.1 ......A.......................T....

CONSENSUS GTTCCTGACTCGTTTAACCGG-CTCTTCCTATATCAGAGAATTAT-TATA

+ Frame 3 S * L V * P - S S Y I R E L - I

60 70 80 90 100

+2 CO477062.2_Pglauca .....................A............................

+9 GE477216.1 .....................G............................

CONSENSUS TCTCTTTCGTTTTACATTTCA-ATACATGGTGTTTCCAGAGTTCAGTCTA

+ Frame 3 S L S F Y I S - T W C F Q S S V Y

110 120 130 140 150

+2 CO477062.2_Pglauca ..................................................

+9 GE477216.1 ..................................................

CONSENSUS CTCGACTAAACCCTAAGCTGTGTAACTCATATCATTTTAGGTTTCAAATC

+ Frame 3 S T K P * A V * L I S F * V S N

160 170 180 190 200

+2 CO477062.2_Pglauca ..................................................

+9 GE477216.1 ..................................................

CONSENSUS CGCTTTGGTTTTATCTTTCCCCAGGAGAGGACTATACAAATATGATCATC

+ Frame 3 P L W F Y L S P G E D Y T N M I I

210 220 230 240 250

+2 CO477062.2_Pglauca ..................................................

+9 GE477216.1 ..................................................

CONSENSUS AGGAGAGGTTCTATGAGCACAGTTATTATAGCTTGCAAGGCCATCATGAC

+ Frame 3 R R G S M S T V I I A C K A I M T

260 270 280 290 300

+2 CO477062.2_Pglauca ..................................................

+9 GE477216.1 ..................................................

CONSENSUS TGTTACACAGTTATTGGCCTTGCTTATCACATGCAGTGCCCTTGCAAGCA

+ Frame 3 V T Q L L A L L I T C S A L A S

310 320 330 340 350

+2 CO477062.2_Pglauca ..................................................

+9 GE477216.1 ..................................................

CONSENSUS CTTCTCTCCAGGCACAAGAGCACCATGAAGGTACCAGACTGGTTGTCATG

+ Frame 3 T S L Q A Q E H H E G T R L V V M

360 370 380 390 400

+2 CO477062.2_Pglauca ..................................................

+9 GE477216.1 ..................................................

CONSENSUS CAAACCAACTTCTTACCTGTGGAGCAGTCAGCCTCCCGCACTCAGTACCA

+ Frame 3 Q T N F L P V E Q S A S R T Q Y Q

410 420 430 440 450

+2 CO477062.2_Pglauca ..................................................

+9 GE477216.1 ..................................................

-3 CO477543.2_Pglauca ...............................

CONSENSUS GTCAGACAGTTCTGTTGAAAACAATAATGGAACGGAGCTTGCAAGCTATC

+ Frame 3 S D S S V E N N N G T E L A S Y

460 470 480 490 500

+2 CO477062.2_Pglauca ..................................................

+9 GE477216.1 ..................................................

-3 CO477543.2_Pglauca ..................................................

CONSENSUS ACTCACTACCAAGAAGGCTGAGGGGAATGGGATGGACATTAGCAGACGGT

+ Frame 3 H S L P R R L R G M G W T L A D G

510 520 530 540 550

+2 CO477062.2_Pglauca .....

+9 GE477216.1 ..................................................

-3 CO477543.2_Pglauca ..................................................

CONSENSUS AAATTAACAAGCGAGGAGCGAAGAACAGAGAAGAAACCCGTCGAAACTCG

+ Frame 3 K L T S E E R R T E K K P V E T R

560 570 580 590 600

+9 GE477216.1 ......

-3 CO477543.2_Pglauca ..................................................

CONSENSUS AACCCATGGTGCTAAAGGGCATGTACATCGCAAGCACGGGGTTGTAACAG

+ Frame 3 T H G A K G H V H R K H G V V T

610 620 630 640 650

-3 CO477543.2_Pglauca ..................................................

CONSENSUS GCGCTGCAGTCTCTAGTCTGGCCTCAGAGTCGAAGAGCCTTCGTCCTTCT

+ Frame 3 G A A V S S L A S E S K S L R P S

660 670 680 690 700

-3 CO477543.2_Pglauca ..................................................

CONSENSUS TCAGGTGCCAGTTCAATGCATACACATGAAGAAACCTCCAAAAGTCATGA

+ Frame 3 S G A S S M H T H E E T S K S H E

710 720 730 740 750

-3 CO477543.2_Pglauca ..................................................

CONSENSUS GTGGACTAGTTTGGTTGGAAAGATGATCAATGTTGTTGATTTGAATAAAC

+ Frame 3 W T S L V G K M I N V V D L N K

760 770 780 790 800

-3 CO477543.2_Pglauca ..................................................

CONSENSUS CTCCTGAAAAATATGATCCGCCGACCTTGAATGCTGACTACCATGGGCCC

+ Frame 3 P P E K Y D P P T L N A D Y H G P

810 820 830 840 850

-3 CO477543.2_Pglauca ..................................................

CONSENSUS AAAACCCACCCTCCCAAGCATAACTGAACTGAAAGCTGCATCACCGGTTT

+ Frame 3 K T H P P K H N * T E S C I T G F

860 870 880 890 900

-3 CO477543.2_Pglauca ..................................................

CONSENSUS TAAATCTGTAGGATGCAGTTCAGAAATCGAATCAATTAACAGCAAGCATG

+ Frame 3 K S V G C S S E I E S I N S K H

910 920 930 940 950

-3 CO477543.2_Pglauca ..................................................

CONSENSUS GTGTTGTTATTGGTTTGGATTCAAGTATTACTTTAAAATCCCTGGGATTG

+ Frame 3 G V V I G L D S S I T L K S L G L

960 970 980 990 1000

-3 CO477543.2_Pglauca ..................................................

CONSENSUS TTAAAAAATAAGTTCTTAGTGTTCAGGGACTAGGACCATTCCTTCATAAA

+ Frame 3 L K N K F L V F R D * D H S F I N

1010 1020 1030 1040 1050

-3 CO477543.2_Pglauca ..................................................

CONSENSUS CGAAATATAGGTTAAAAGAGAGACGAGAGGAAATACAAAATATTGTCTGG

+ Frame 3 E I * V K R E T R G N T K Y C L

1060 1070 1080 1090 1100

-3 CO477543.2_Pglauca ..................................................

CONSENSUS GCTAACATGTGGCTAAGTCAGGTTGTGTTCCATGCAAAAGGAGTATCAGA

+ Frame 3 G * H V A K S G C V P C K R S I R

1110 1120 1130

-3 CO477543.2_Pglauca ...................................

CONSENSUS TATATATTTAACTTGCTTTCCTTAAAAACAAAAAA

+ Frame 3 Y I F N L L S L K T K -

1. ***Picea glauca CLEL15***

10 20 30 40 50

+7 EX387983.1_Pglauca ..................................................

CONSENSUS ATTGCAATGGGCAGGAGAAGAGTCACGATGACACTTGAGAGATCTGAGTA

+ Frame 2 L Q W A G E E S R * H L R D L S

60 70 80 90 100

+7 EX387983.1_Pglauca ..................................................

CONSENSUS ACTGGAAGCCTGTAATTAGCATAAGATCGAATCCTTGATCAGTGTAGGGT

+ Frame 2 N W K P V I S I R S N P * S V * G

110 120 130 140 150

+7 EX387983.1_Pglauca ..................................................

CONSENSUS GGAATGTTATTCAAACTTTTTTTATGGTGTTTATCACTACAAGATAGGAG

+ Frame 2 G M L F K L F L W C L S L Q D R S

160 170 180 190 200

+7 EX387983.1_Pglauca ..................................................

+6 EX379031.1_Pglauca ..............................

CONSENSUS TATCGCAGAAAAAGATAGTGATCAGTATTGAAAAATCAAGATCATTATAA

+ Frame 2 I A E K D S D Q Y * K I K I I I

210 220 230 240 250

+7 EX387983.1_Pglauca ..................................................

+6 EX379031.1_Pglauca ..................................................

CONSENSUS TTGACAATCCGAGACGAGAAGATCTTATAACTGTTATTTACAGAAGAAAA

+ Frame 2 I D N P R R E D L I T V I Y R R K

260 270 280 290 300

+7 EX387983.1_Pglauca ............A..........................C..........

+6 EX379031.1_Pglauca ............G..........................T..........

CONSENSUS ATGATGGCCACC-CCTGCAAGATCGTTGTAATTATGCTC-TTGCATTGTT

+ Frame 2 M M A T T/A C K I V V I M L L/F A L F

310 320 330 340 350

+7 EX387983.1_Pglauca ..................................................

+6 EX379031.1_Pglauca ..................................................

CONSENSUS TTTTCTCCCGTCTAATTGCAGACAGATGCCCAATGAAGCAGCTCAACTGA

+ Frame 2 F L P S N C R Q M P N E A A Q L

360 370 380 390 400

+7 EX387983.1_Pglauca ..................................................

+6 EX379031.1_Pglauca ..................................................

CONSENSUS GAAAATGGAATTTACAGCCCGCCTTTTTTGGTCGTAGGGATGGTGGAGTA

+ Frame 2 R K W N L Q P A F F G R R D G G V

410 420 430 440 450

+7 EX387983.1_Pglauca ............................................G.....

+6 EX379031.1_Pglauca ............................................C.....

CONSENSUS TCACAGGACAGTAGCGTCCTTGTAGGCTATTACAAACTGGAAGC-AATGC

+ Frame 2 S Q D S S V L V G Y Y K L E A N A

460 470 480 490 500

+7 EX387983.1_Pglauca ..................................................

+6 EX379031.1_Pglauca ..................................................

CONSENSUS TAAGCTCAGAGGACTGAACGATATTGAAAGAAGATTTATAGAACGCCTGG

+ Frame 2 K L R G L N D I E R R F I E R L

510 520 530 540 550

+7 EX387983.1_Pglauca .......A...............................C..........

+6 EX379031.1_Pglauca .......G...............................T..........

CONSENSUS GAGAGGA-AATACTGCAGGGATGCCTGAGAAGCATTCGT-TTTGAAGCGA

+ Frame 2 G E E N T A G M P E K H S S/F L K R

560 570 580 590 600

+7 EX387983.1_Pglauca ..................................................

+6 EX379031.1_Pglauca .......................................A.....G....

-8 EX388347.1_Pglauca .................

CONSENSUS ATCCTATCAATAGTATCTGGCTCGAGGCCTTCAATGAAGTTGGGCTATCA

+ Frame 2 I L S I V S G S R P S M K L/M G Y/D Q

610 620 630 640 650

+7 EX387983.1_Pglauca ..................................................

+6 EX379031.1_Pglauca ..................................................

-8 EX388347.1_Pglauca ..................................................

CONSENSUS AAATAAGTTCTCAGGAACCCAAGAGGCAAAATCCTCCAGTAGAACGTTAT

+ Frame 2 N K F S G T Q E A K S S S R T L

660 670 680 690 700

+7 EX387983.1_Pglauca ..................................................

+6 EX379031.1_Pglauca ..........................A...............G.......

-8 EX388347.1_Pglauca ..................................................

CONSENSUS GTAACATCCCAGAAACAAGCAAGGCCGGGCCAATGGATGAAGAAGCTGCC

+ Frame 2 C N I P E T S K A G/W P M D E E/G A A

710 720 730 740 750

+7 EX387983.1_Pglauca ..................................................

+6 EX379031.1_Pglauca ..................................................

-8 EX388347.1_Pglauca ..................................................

CONSENSUS ATGATTCCACCTAAACCACAGGGAGACAAAGATGATGGAGACGTTGTCAA

+ Frame 2 M I P P K P Q G D K D D G D V V N

760 770 780 790 800

+7 EX387983.1_Pglauca ..................................................

+6 EX379031.1_Pglauca ..................................................

-8 EX388347.1_Pglauca ..................................................

CONSENSUS CGCAATGGACTATGCTCCTGCGCACAAGAAGCCTCCCATACACAACTAAT

+ Frame 2 A M D Y A P A H K K P P I H N *

810 820 830 840 850

+7 EX387983.1_Pglauca ...............................TCA................

+6 EX379031.1_Pglauca ..................................................

-8 EX388347.1_Pglauca ..................................................

CONSENSUS TATCTGGATAATATTTACAGGAAAATGGTTTCAGGATTCACCTACTTATC

+ Frame 2 L S G * Y L Q E N G F R I H L L I

860 870 880 890 900

+7 EX387983.1_Pglauca ...

+6 EX379031.1_Pglauca ...........................A......................

-8 EX388347.1_Pglauca ...........................G......................

CONSENSUS TACTTTCAGCCCGAGCCTTGTCTTGTC-CCTTAAATTATATTGGAAGCAT

+ Frame 2 Y F Q P E P C L V - L N Y I G S I

910 920 930 940 950

+6 EX379031.1_Pglauca .......................................A..........

-8 EX388347.1_Pglauca .......................................G..........

CONSENSUS TCTGTCCTATTGGTGAAAAATCCTTTACTGTTCGACAAA-GAACATGAAA

+ Frame 2 L S Y W * K I L Y C S T - E H E

960 970 980 990 1000

+6 EX379031.1_Pglauca ..................................................

-8 EX388347.1_Pglauca ..................................................

CONSENSUS TATCCTTTCCTCCCCAGAAAAGAGTGTACGAATTATGGCATTATGCACCC

+ Frame 2 I S F P P Q K R V Y E L W H Y A P

1010 1020 1030 1040 1050

+6 EX379031.1_Pglauca ...............................................C..

-8 EX388347.1_Pglauca ...............................................T..

CONSENSUS ATCAGAAAATAGCATATGAATTATGGAGCATATTGTGCATTCATTCA-GC

+ Frame 2 I R K * H M N Y G A Y C A F I - A

1060 1070 1080 1090 1100

+6 EX379031.1_Pglauca ...................

-8 EX388347.1_Pglauca ..................................................

CONSENSUS AGATATGTAAATGGTAAATCCATCAGAAAATAGCATATGAATTATGGCAG

+ Frame 2 D M * M V N P S E N S I * I M A

1110 1120 1130 1140 1150

-8 EX388347.1_Pglauca ..................................................

CONSENSUS ATATTGTGCATCAGAAAATAGCATATGAACTATGGCAGATATTGTGCATT

+ Frame 2 D I V H Q K I A Y E L W Q I L C I

1160 1170 1180 1190 1200

-8 EX388347.1_Pglauca ..................................................

CONSENSUS CATTCACACAGATATGTAAATGGTAAATCCATCAGAAAATAGCATATGAA

+ Frame 2 H S H R Y V N G K S I R K * H M N

1210 1220 1230 1240 1250

-8 EX388347.1_Pglauca ..................................................

CONSENSUS TTATGGCAGATATTGTGCATTCATTCACACACAGATATGTAAATGGTAAA

+ Frame 2 Y G R Y C A F I H T Q I C K W *

1260 1270 1280 1290 1300

-8 EX388347.1_Pglauca ..................................................

CONSENSUS GAGTTGTTAATGACAAGTACCTTTCTTCAAGTGTTGGGAGGAGGTCTTGA

+ Frame 2 R V V N D K Y L S S S V G R R S *

1310 1320 1330 1340 1350

-8 EX388347.1_Pglauca ..................................................

CONSENSUS GGTCAAATCGGGCTGGGTTTCCGGGACGAATTGTAATGGCTACTTGTGAT

+ Frame 2 G Q I G L G F R D E L * W L L V M

1360 1370 1380 1390 1400

-8 EX388347.1_Pglauca ..................................................

CONSENSUS GTAAATGATTTATATCTCTAATATAAATCGAGTTAATTTCTCGTTGAACC

+ Frame 2 * M I Y I S N I N R V N F S L N

-8 EX388347.1_Pglauca ......

CONSENSUS AAAAAA

+ Frame 2 Q K

1. ***Picea sitchensis CLEL16***

10 20 30 40 50

+10 GT121615.1_Psitchens ..................................................

CONSENSUS TCCCAAGAAGGCTGAGGGGAATGGGATGGACATTAGCAGACGGTAAATTA

+ Frame 3 P R R L R G M G W T L A D G K L

60 70 80 90 100

+10 GT121615.1_Psitchens ..................................................

CONSENSUS ACAAGCGAGGAGCGAAGAACAGAGAAGAAACCCGTCGAAACTCGAACCCA

+ Frame 3 T S E E R R T E K K P V E T R T H

110 120 130 140 150

+10 GT121615.1_Psitchens ..................................................

CONSENSUS TGGTGCTAAAGGGCATGTACATCGCAAGCACGGGGTTGTAACAGGCGCTG

+ Frame 3 G A K G H V H R K H G V V T G A

160 170 180 190 200

+10 GT121615.1_Psitchens ..................................................

CONSENSUS CAGTCTCTAGTCTGGCCTCAGAGTCGAAGAGCCTTCGTCCTTCTTCAGGA

+ Frame 3 A V S S L A S E S K S L R P S S G

210 220 230 240 250

+10 GT121615.1_Psitchens ..................................................

CONSENSUS ATCCAGTCCAATTTAAGCTCTCCACAGGAAAGCAAGGTCATTTCAGAGGA

+ Frame 3 I Q S N L S S P Q E S K V I S E E

260 270 280 290 300

+10 GT121615.1_Psitchens ..................................................

CONSENSUS GAAAATGGATGGACTCGCAATTCAGGAATTGGCTATAACAACTAAGAGAT

+ Frame 3 K M D G L A I Q E L A I T T K R

310 320 330 340 350

+10 GT121615.1_Psitchens ..................................................

CONSENSUS CATTGAAGTCCATTGCTAGCAGCCCTAGCGACGGGCAAGAGAAAGAAGAG

+ Frame 3 S L K S I A S S P S D G Q E K E E

360 370 380 390 400

+10 GT121615.1_Psitchens ..................................................

CONSENSUS ATTTCCAGTGAATCGAACAGTTATAAGCCTGCTCCACCAAGTGCTACTAG

+ Frame 3 I S S E S N S Y K P A P P S A T S

410 420 430 440 450

+10 GT121615.1_Psitchens ..................................................

CONSENSUS TGGACATAGGGCTCAGGCATCTCCGACTCCAGAAGCTCAAACATTCCACG

+ Frame 3 G H R A Q A S P T P E A Q T F H

460 470 480 490 500

+10 GT121615.1_Psitchens ..................................................

CONSENSUS TCTCCGGGCCGATGACATCTGAATCGAACGACGCGTCAGACCTTTCCGAC

+ Frame 3 V S G P M T S E S N D A S D L S D

510 520 530 540 550

+10 GT121615.1_Psitchens ..................................................

CONSENSUS ATCATAGGCATGGACTATGGTCGTGCACGCAGAAATCCACCTATTCACAA

+ Frame 3 I I G M D Y G R A R R N P P I H N

560 570 580 590 600

+10 GT121615.1_Psitchens ..................................................

CONSENSUS CAAAGCTCCTAAACCATAGTTGTGTTTTATATAGGGCTTCAAATAATTTT

+ Frame 3 K A P K P * L C F I * G F K * F

610 620 630 640 650

+10 GT121615.1_Psitchens ..................................................

CONSENSUS TGATACTGAGTATGTATAAGAAAATAATTTATAAAGGCATTAAAACGTAT

+ Frame 3 L I L S M Y K K I I Y K G I K T Y

660 670 680 690 700

+10 GT121615.1_Psitchens ..................................................

CONSENSUS ATAATACAGTATAATCTCGCCTCTGCCAGAGATCGTCCATGGTTTTGAGT

+ Frame 3 I I Q Y N L A S A R D R P W F * V

710 720 730 740 750

+10 GT121615.1_Psitchens ..................................................

CONSENSUS CAGGGTATTAGCTAGGTTGGTTTCCACATACATACGTTTTAGATATCGTA

+ Frame 3 R V L A R L V S T Y I R F R Y R

760 770 780 790 800

+10 GT121615.1_Psitchens ..................................................

CONSENSUS ATTTATTGTGTTCTGTACTGATATCTGCTATACTTTTTTAATACAAATAT

+ Frame 3 N L L C S V L I S A I L F * Y K Y

810 820 83

+10 GT121615.1_Psitchens .............................

CONSENSUS ACTAGTTTGGCAAAAAAAAAAAAAAAAAA

+ Frame 3 T S L A K K K K K -

1. ***Pinus taeda CLEL17***

10 20 30 40 50

+5 DR744288.1_Ptaeda ..................................................

CONSENSUS CTGACTGTTACAGAGTTCTTGGCATTGCTTATCATATGCAGTGCCCTTGC

+ Frame 1 L T V T E F L A L L I I C S A L A

+ Frame 2 * L L Q S S W H C L S Y A V P L

60 70 80 90 100

+5 DR744288.1_Ptaeda ..................................................

CONSENSUS AAGCACTTCTCTGCAGGCACGAGCCCACCATGAAGGTACCGGACTGGTTG

+ Frame 1 S T S L Q A R A H H E G T G L V

+ Frame 2 Q A L L C R H E P T M K V P D W L

110 120 130 140 150

+5 DR744288.1_Ptaeda ..................................................

CONSENSUS GCAGGCGAACCAACTTCTTCAATGTGGAGCAGTCAGCCTCCCACACTCAG

+ Frame 1 G R R T N F F N V E Q S A S H T Q

+ Frame 2 A G E P T S S M W S S Q P P T L R

160 170 180 190 200

+5 DR744288.1_Ptaeda ..................................................

+4 DR744221.1_Ptaeda ...............................

CONSENSUS AACCAATCAGACAGCTCTGTTGAAAACAATAATGGAATGGAGCTTGCAAG

+ Frame 1 N Q S D S S V E N N N G M E L A S

+ Frame 2 T N Q T A L L K T I M E W S L Q

210 220 230 240 250

+5 DR744288.1_Ptaeda .......*..........................................

+4 DR744221.1_Ptaeda .......T..........................................

CONSENSUS CGATCGC-TCAATACCAACAAGGCTGAGGCGAATGGAATCGACACAAGCA

+ Frame 1 D R

+ Frame 2 A I A S I P T R L R R M E S T Q A

260 270 280 290 300

+5 DR744288.1_Ptaeda ..................................................

+4 DR744221.1_Ptaeda ..................................................

CONSENSUS GACCGTAAATTAACAAACAAGGACAGAATAACAGAGAAGAAACCCATACA

+ Frame 2 D R K L T N K D R I T E K K P I Q

310 320 330 340 350

+5 DR744288.1_Ptaeda ..................................................

+4 DR744221.1_Ptaeda ..................................................

CONSENSUS AACTCGAACTGGTGCTGCTAATGGGAATGTACTTCGCAAGCCTGAGGCTG

+ Frame 2 T R T G A A N G N V L R K P E A

360 370 380 390 400

+5 DR744288.1_Ptaeda ..................................................

+4 DR744221.1_Ptaeda ..................................................

CONSENSUS TAAAAGGAGCTGCACACTCTAGTCTGGCCTCAAAGTCTAAGAGACTTCGT

+ Frame 2 V K G A A H S S L A S K S K R L R

410 420 430 440 450

+5 DR744288.1_Ptaeda ..................................................

+4 DR744221.1_Ptaeda ..................................................

CONSENSUS CCTTCCTCAGGTGCCAGTTCAATAAATACACATGAAGAAATCTCCAAAAG

+ Frame 2 P S S G A S S I N T H E E I S K S

460 470 480 490 500

+5 DR744288.1_Ptaeda ..................................................

+4 DR744221.1_Ptaeda ..................................................

CONSENSUS TCATGAATTGACTAGCTTGGTGGGAAAGATGATCAATGTTGTTGATTTGA

+ Frame 2 H E L T S L V G K M I N V V D L

510 520 530 540 550

+5 DR744288.1_Ptaeda ..................................................

+4 DR744221.1_Ptaeda ..................................................

CONSENSUS ATAAACCTCATGAAAAATATGATCAGCCATCCTTGAATGCTGACTACCAT

+ Frame 2 N K P H E K Y D Q P S L N A D Y H

560 570 580 590 600

+5 DR744288.1_Ptaeda ..................................................

+4 DR744221.1_Ptaeda ..................................................

CONSENSUS GGGCCCAAAACCCACCCTCCCAAGCATAACTGAACTGGGAGCTGCATCAC

+ Frame 2 G P K T H P P K H N * T G S C I T

610 620 630 640 650

+5 DR744288.1_Ptaeda ..................................................

+4 DR744221.1_Ptaeda ..................................................

CONSENSUS TGGTTTTAAATATGTAGGATGCAGCTCAGGAATCAAATCAACTGAAAGCA

+ Frame 2 G F K Y V G C S S G I K S T E S

660 670 680 690 700

+5 DR744288.1_Ptaeda ..................................................

+4 DR744221.1_Ptaeda ..................................................

CONSENSUS AGGATGGTGGTGTTATTGGTTTGGGTTCAACTATTACTTTAAAATCTCTG

+ Frame 2 K D G G V I G L G S T I T L K S L

710 720 730 740 750

+5 DR744288.1_Ptaeda ..................................................

+4 DR744221.1_Ptaeda ..................................................

CONSENSUS GGATTGTTGGAAAAATAAGTTCACTGTACAGGGACTAGGACTATTCCTTC

+ Frame 2 G L L E K * V H C T G T R T I P S

760 770 780 790 800

+5 DR744288.1_Ptaeda ..................................................

+4 DR744221.1_Ptaeda ..................................................

CONSENSUS ATAAACGAAATATAGGCTAAAAGAGAGATGAGATGAGATACATAATGTTG

+ Frame 2 * T K Y R L K E R * D E I H N V

810 820 830 840 850

+5 DR744288.1_Ptaeda ...................

+4 DR744221.1_Ptaeda ..................................................

CONSENSUS TGGAGGCTAACCTGTGGCTAAGCCAGGTTGTGACCCATGCAAACGGAGTA

+ Frame 2 V E A N L W L S Q V V T H A N G V

860 870 880 890 900

+4 DR744221.1_Ptaeda ..................................................

CONSENSUS TCCGATATATTTAACTTGTTTTCCTTTCATGTATGCTATAACTATATCCA

+ Frame 2 S D I F N L F S F H V C Y N Y I Q

910 920 930 940 950

+4 DR744221.1_Ptaeda ..................................................

CONSENSUS ATTTTGAATATGATGAAAATTTTCTTGTGAGTGGATAAATGTTGTTATGG

+ Frame 2 F * I * * K F S C E W I N V V M

960 970 980 990 1000

+4 DR744221.1_Ptaeda ..................................................

CONSENSUS TATGCAACCCTGCATGTAACATGAGTTGAAATACTTCAACCACTTTGGTC

+ Frame 2 V C N P A C N M S * N T S T T L V

1010 1020 10

+4 DR744221.1_Ptaeda ............................

CONSENSUS ATATGATAATCTGCATCAGTAAAAAGAA

+ Frame 2 I * * S A S V K R
