# Supplementary material for: Bioinformatic and phylogenetic analysis of the CLAVATA3/EMBRYO-SURROUNDING REGION (CLE) and the CLE-LIKE signal peptide genes in the Pinophyta
Source: BMC Plant Biol. 2014 Feb 14;14:47. doi: 10.1186/1471-2229-14-47 (PMC4016512; doi:10.1186/1471-2229-14-47)
Supplement: Additional file 4: Figure S3 — Multiple alignment of predicted Pinophyta CLEL peptide amino acid sequences. Sequences are arranged as depicted in Figure 4B with conifer sequences grouped with their closest Arabidopsis homologue. Closest matching Arabidopsis-Pinophyta homologues are positioned directly beneath their putative closest Arabidopsis homologue Arabidopsis gene names are signified with grey highlighting. Mismatches in the Pinophyta sequences from their closest Arabidopsis homologues are indicated by black highlighting and inverse lettering. [file 1471-2229-14-47-S4.docx]

**Figure S3: Multiple alignment of predicted Pinophyta CLEL peptide amino acid sequences.** Sequences are arranged as depicted in Fig. 4B with conifer sequences grouped with their closest *Arabidopsis* homologue. Closest matching *Arabidopsis*-Pinophyta homologues are positioned directly beneath their putative closest *Arabidopsis* homologue *Arabidopsis* gene names are signified with *grey highlighting.* Mismatches in the Pinophyta sequences from their closest *Arabidopsis* homologues are indicated by *black highlighting* and *inverse lettering.*

>Athal_CLEL6 D Y - - - P Q P H R K P P I H N

>Pglau_CLEL15 D Y - - - A P A H K K P P I H N

>Pabie CLEL20 D Y - - - A P A H K K P P I H N

>Pglau CLEL25 D Y - - - T G P K T H P P K N N

>Pabie CLEL26 D Y - - - T G P K T H P P K N N

>Athal_CLEL9 D Y - - - N S A N K K R P I H N

>Athal_CLEL4 D Y - - - G Q R K Y K P P V H N

>Athal_CLEL5 D Y - - Q G P K P R S K P L K N

>Athal_CLEL1 D Y - - - W K P R H H P P K N N

>Athal_CLEL3 D Y - - - W R A K H H P P K N N

>Athal_GLV9 E Y P S P V K P R K R T P V H N

>Pglau CLEL27 D Y - - - S G P K T H P P K N N

>Pabie CLEL28 D Y - - - S G P K T H P P K N N

>Athal_CLEL2 D Y - - - R T F R R R R P V H N

>Athal_CLEL7 D Y - - - P K P S T R P P R H N

>Athal_CLEL8 D Y - - - S N P G H H P P R H N

>Athal_CLE18 D Y - - - E S H H A H S P I H N

>Pglau CLEL19 D Y - - - S G P K T H N P K H H

>Pabie CLEL21 D Y - - - S G P K T H N P K H H

>Pexg_CLEL13 D Y - - - S G P K T H N P K H H

>Athal_RGF4 D Y P I Y S K P R R K P P V N N

>Psitc_CLEL16 D Y - - - G R A R R N P P I H N

>Pglau CLEL22 D Y - - - G R A R R N P P I H N

>Pabie CLEL23 D Y - - - G R A R R N P P I H N

>Pabie CLEL24 D Y - - - G R A R R N P P I H N

>Pglau_CLEL14 D Y - - - H G P K T H P P K H N

>Ptaed_CLEL17 D Y - - - H G P K T H P P K H N

>Pabie CLEL18 D Y - - - H G P K T H P P K H N
